# Intermittent auscultation fetal monitoring during labour: A systematic scoping review to identify methods, effects, and accuracy

**DOI:** 10.1371/journal.pone.0219573

**Published:** 2019-07-10

**Authors:** Ellen Blix, Robyn Maude, Elisabeth Hals, Sezer Kisa, Elisabeth Karlsen, Ellen Aagaard Nohr, Ank de Jonge, Helena Lindgren, Soo Downe, Liv Merete Reinar, Maralyn Foureur, Aase Serine Devold Pay, Anne Kaasen

**Affiliations:** 1 Faculty of Health Sciences, OsloMet–Oslo Metropolitan University, Oslo, Norway; 2 Graduate School of Nursing, Midwifery, and Health, Victoria University, Wellington, NZ; 3 Department of Obstetrics and Gynaecology, Innlandet Hospital Trust, Lillehammer, Norway; 4 University Library, OsloMet–Oslo Metropolitan University, Oslo, Norway; 5 Research Unit for Gynaecology and Obstetrics, Institute of Clinical Research, University of Southern Denmark, Odense, Denmark; 6 Department of Midwifery Science, Amsterdam UMC, AVAG, and the Amsterdam Public Health Research Institute, Amsterdam, The Netherlands; 7 Department of Women’s and Children’s Health, Karolinska Institute, Stockholm, Sweden; 8 School of Community Health and Midwifery, University of Central Lancashire, Preston, United Kingdom; 9 Management and Staff for Health Service, Norwegian Institute of Public Health, Oslo, Norway; 10 Faculty of Health, University of Technology, Sydney, Australia; 11 Department of Gynaecology and Obstetrics, Oslo University Hospital, Oslo, Norway; University of Mississippi Medical Center, UNITED STATES

## Abstract

**Background:**

Intermittent auscultation (IA) is the technique of listening to and counting the fetal heart rate (FHR) for short periods during active labour and continuous cardiotocography (CTC) implies FHR monitoring for longer periods. Although the evidence suggests that IA is the best way to monitor healthy women at low risk of complications, there is no scientific evidence for the ideal device, timing, frequency and duration for IA. We aimed to give an overview of the field, identify and describe methods and practices for performing IA, map the evidence and accuracy for different methods of IA, and identify research gaps.

**Methods:**

We conducted a systematic scoping review following the Joanna Briggs methodology. Medline, EMBASE, Cinahl, Maternity & Infant Care, Cochrane Library, SveMed+, Web of Science, Scopus, Lilacs and African Journals Online were searched for publications up to January 2019. We did hand searches in relevant articles and databases. Studies from all countries, international guidelines and national guidelines from Denmark, United Kingdom, United States, New Zealand, Australia, The Netherlands, Sweden, Denmark, and Norway were included. We did quality assessment of the guidelines according to the AGREEMENT tool. We performed a meta-analysis assessing the effects of IA with a Doppler device vs. Pinard device using methods described in *The Cochrane Handbook*, and we performed an overall assessment of the summary of evidence using the GRADE approach.

**Results:**

The searches generated 6408 hits of which 26 studies and 11 guidelines were included in the review. The studies described slightly different techniques for performing IA, and some did not provide detailed descriptions. Few of the studies provided details of normal and abnormal IA findings. All 11 guidelines recommended IA for low risk women, although they had slightly different recommendations on the frequency, timing, and duration for IA, and the FHR characteristics that should be observed. Four of the included studies, comprising 8436 women and their babies, were randomised controlled trials that evaluated the effect of IA with a Doppler device vs. a Pinard device. Abnormal FHRs were detected more often using the Doppler device than in those using the Pinard device (risk ratio 1.77; 95% confidence interval 1.29–2.43). There were no significant differences in any of the other maternal or neonatal outcomes. Four studies assessed the accuracy of IA findings. Normal FHR was easiest to identify correctly, whereas identifying periodic FHR patterns such as decelerations and saltatory patterns were more difficult.

**Conclusion:**

Although IA is the recommended method, no trials have been published that evaluate protocols on how to perform it. Nor has any study assessed interrater agreements regarding interpretations of IA findings, and few have assessed to what degree clinicians can describe FHR patterns detected by IA. We found no evidence to recommend Doppler device instead of the Pinard for IA, or vice versa.

## Introduction

The aim of fetal heart monitoring during labour is to monitor the health of the fetus, identify those at risk of neonatal and long-term injury, reassure labouring women and staff that all is well, and intervene in a timely manner when deviations from normal are observed. Although a range of techniques are available for fetal monitoring during labour, including maternal perception of fetal movements, the current standard is to assess the fetal heart rate (FHR) in conjunction with uterine contractions. There are two main modalities for FHR monitoring–Intermittent auscultation (IA) and continuous electronic monitoring using cardiotocography (CTG). Organizations and stakeholders like the World Health Organization, International Confederation of Midwives, and the International Federation of Gynecology and Obstetrics (FIGO) recommend that IA should be used to monitor uncomplicated (low-risk) pregnancies during labour and birth and for pregnancies in settings where no alternatives are available [1–3]. A large RCT performed during the 1980s comparing IA and CTG showed that CTG was associated with a lower risk of neonatal seizures in babies born following prolonged labour and/or labour augmented with oxytocin (medication to speed up the labour process) [4]. Although CTG monitoring was not associated with long-term damage, such as cerebral palsy, it was associated with increased risk of the need for a caesarean section and operative vaginal delivery. Furthermore, it restricted the mobility of women in labour [4, 5].

IA is the technique of listening to and counting the fetal heartbeats for short periods of time during active labour. It is usually performed using a Pinard stethoscope or a hand-held Doppler device, with the uterine contractions palpated by hand. The Pinard device is a hollow tube often composed of wood or metal. The size of the Pinard varies from one country to another, ranging from 15 to 60 cm. It amplifies sounds associated with the closing of the fetal heart valves during each fetal cardiac cycle. With this device, the midwife can thus hear fetal heart sounds, including any abnormal heartbeat rhythms, in real time, rather than from a secondary source [6]. The advantages of the Pinard device are it is low cost, available in all settings and no consumables are needed.

The Doppler device is a small hand-held ultrasound transducer that uses the “Doppler effect” to provide an audible simulation of the fetal heartbeats. Its advantages are that it can be used in various maternal positions, including water immersion, and that the real-time audio sound is shared with everyone present in the room. It is more expensive than the Pinard, and consumables like batteries and spare parts are needed.

In contrast, although the evidence suggests that IA is the best way to monitor healthy women with healthy pregnancies at low risk of complications, many maternity care providers prefer continuous CTG for all women [7].

Unfortunately, because CTG is used far more often than recommended in high-, medium-, and low-risk settings worldwide, skills needed to perform IA–especially when using the Pinard device–are rapidly disappearing from midwifery practice [8, 9], reducing the opportunity for women to benefit from the use of this technique. To our knowledge, there is no scientific evidence for the ideal device, timing, frequency and duration for IA.

This systematic scoping review aimed to give an overview of the field, identify and describe methods and practices for performing IA, map the evidence (or lack thereof) for various methods, and identify research gaps. Specific aims were to 1) systematically map the techniques and protocols for performing IA; 2) map any effect or evidence of the accuracy of IA; and 3) map the findings to support recommendations given in international and national guidelines.

## Methods

A study protocol was developed and published before initiating the literature search [10]. We then conducted a scoping review following the methods described by the Joanna Briggs Institute [11]. In March 2017, we completed a systematic literature search in MEDLINE, EMBASE, Cinahl, Maternity & Infant Care, Cochrane Library, SveMed+, Web of Science, Scopus, Lilacs, and African Journals Online (AJOL). The first step was an initial search of two databases relevant to the topic (MEDLINE and Cinahl) to identify relevant search terms related to labour and IA. Then, a highly sensitive search strategy (step two) was developed using relevant subject headings (e.g., MeSH) and free-text words (.tw) for each of the study components and adapted it to all included databases. In January 2019, we updated the entire search. The search is described in detail in the S1 Table.

Each search was designed broadly, regardless of the study design and language. We included scientific studies that described or assessed all forms of IA during labour, and in languages understood by members of the study group (Danish, Dutch, English, French, German, Icelandic, Norwegian, Swedish and Turkish).

We also included FHR monitoring guidelines in the review. We limited the search for guidelines to international guidelines and national guidelines from the United Kingdom, Australia, New Zealand, United States, Canada, Denmark, Norway, Sweden and The Netherlands. Exclusion criteria were: studies or guidelines published in other languages than those mentioned above, articles not assessed as scientific studies, not containing information according to the aims of the scoping review, commentaries, expert opinions, letters to the editor, review or if we were unable to retrieve the article in full text.

A senior librarian (EK) performed the literature searches. Two reviewers then independently screened all titles and abstracts to determine eligibility based on the inclusion criteria and labelled the articles as “included,” “excluded,” or “uncertain” (AK, EB). All articles labelled “included” or “uncertain” by at least one of the reviewers underwent full-text assessment by pairs of reviewers (AK, EH, ASDP, SK, EB). Disagreements were discussed by a group of reviewers until consensus was reached.

A data charting form was developed and completed for each study. For scientific articles, relevant data included a description of the population, sample size, concept (phenomena of interest or study aim), context (details about the study setting), device used for IA, details on how IA was performed, any effects of the methods of performing IA, and results from studies reporting IA accuracy. Regarding IA, accuracy is how well observers can describe IA characteristics compared to a pre-defined reference standard. Recommendations for IA were extracted from the included guidelines.

According to the Joanna Briggs methodology [11], we did not perform quality assessments of the studies that described devices or modes for performing IA. We did perform quality assessments of the included guidelines by assessing if: 1) all relevant areas and/or professions were included; 2) views and preferences of target populations were sought; 3) the targeted users were defined; 4) systematic methods were used to search for evidence; 5) criteria for selecting evidence were clearly described; 6) strengths and limitations of the body of evidence were clearly described; 7) methods for formulating recommendations were clearly described; 8) the guidelines were externally reviewed by experts before publication [12].

We did descriptive summaries of studies and guidelines. The trials comparing IA with the Doppler device vs. Pinard device were combined in a meta-analysis performed according to the methods described in the *Cochrane Handbook* [13]. Studies that met the inclusion criteria were critically appraised using the “risk of bias’’’ tool, and outcomes were analysed by calculating the pooled risk ratio with 95% confidence intervals and a random-effects model. We also conducted sensitivity analyses [13] and an overall assessment of the summary of evidence using the Grading of Recommendations Assessment, Development, and Evaluation (GRADE) approach [14].

## Results

The electronic search generated 6401 hits, with another 7 studies found by hand search. After deleting duplicates, 4081 records were screened by reading their titles and abstracts. At this stage, 3804 records were excluded, mainly because they were unrelated to the topic of interest. Altogether, 276 papers then underwent full-text review, resulting in 239 papers being excluded. Finally, 37 papers (26 articles [4, 9, 15–38] and 11 guidelines [1–3, 39–46]) were included in the review. Characteristics of the included papers are shown in Table 1 and Table 2.

**Table 1:** Characteristics of included studies.

Author, year, country	Aim of study	Design and study population	Information of interest
*Studies describing devices and modes of performing intermittent auscultation (IA)*			
Smith et al, 2019, Ireland [38]	Compare the effect on caesarean section rates of admission CTG vs. IA	RCT 3034 low-risk women in labour (1513 IA, 1521 admission CTG)	Descriptions of IA practices (device, frequency, timing, duration)
Kamala et al, 2018, Tanzania [37]	Compare continuous fetal heart rate monitoring using the Moyo strap-on monitor with IA using a Pinard for the detection of FHR abnormalities	Pre- and post-intervention study. 1640 low-risk women monitored with a Pinard and 2442 with a Moyo device	Descriptions of IA practice (device, frequency, timing, abnormal FHR)
Maude et al, 2014, NZ [9]	Describe the implementation of the Intelligent Structured Intermittent Auscultation (ISIA) framework in one maternity unit	Mixed method pre- and post-intervention study. Audit of 511 medical records before intervention and 422 after intervention	Descriptions of IA practices (device, frequency, timing, duration, definitions of normal and abnormal FHR, assessments of uterine contractions, fetal movements and maternal pulse). Presentation and evaluation of a decision-making framework for fetal heart monitoring in low-risk women
Rathore et al, 2011, India [19]	Evaluate fetal scalp stimulation test as an adjunct to IA in diagnosis of intrapartum fetal acidosis	Prospective observational cohort 750 women in labour, with fetal heart abnormalities and/or thick meconium stained amniotic fluid	Descriptions of IA practices (device, frequency, timing, duration)
Maude et al, 2009, NZ [17]	Explore the fetal heart rate monitoring practices of midwives and doctors and determine compliance with a NZ evidence-based guideline for fetal heart monitoring	Retrospective audit of 193 randomly selected medical records undertaken over six months in 2006	Descriptions of IA practices (device, frequency, timing, duration, definitions of normal and abnormal FHR, assessments of uterine contractions, fetal movements and maternal pulse, documentation practices for IA).
Soltani, 2009, Iran [20]	Present and evaluate a new electronic device for IA	Presentation of a new device for IA, with evaluations from 28 medical trainees	Description of a device for IA, a Personal Digital Assistant, an electronic stethoscope attached to a hand-held computer.
Madaan & Trivedi, 2006, India [21]	Compare the effect of EFM and IA for intrapartum fetal monitoring	RCT 100 women included (50 EFM, 50 IA), with post caesarean pregnancies and no contraindications for a vaginal delivery	Descriptions of IA practices (frequency, timing, duration, definition of abnormal FHR)
Impey et al, 2003, Ireland [22]	Compare the effect on neonatal outcome of admission CTG vs. IA	RCT 8580 low-risk women included (4320 admission CTG, 4308 IA)	Descriptions of IA practices (frequency, timing, duration)
Mires et al, 2001, UK [23]	Compare the effect on neonatal outcome of admission CTG vs. IA and levels of obstetric interventions in a low-risk obstetric population	RCT 3751 low-risk women included (1866 admission CTG, 1885 IA)	Descriptions of IA practices (device, duration)
Gilles et al, 1997, Australia [25]	Survey the use of IA throughout maternity units in Western Australia, compare protocols and suggest a protocol for use in women with low-risk labours	Survey to all hospitals in Western Australia	Descriptions of IA practices (frequency, definition abnormal FHR). A new protocol for IA based on review of practices and research literature
Vintzileos et al, 1993, Greece [27]	Compare the effect on neonatal outcome of EFM vs. IA	RCT 1428 women with singleton living fetus and gestational age ≥26 weeks included (746 EFM, 682 IA).	Description of IA practices (device, frequency, timing, duration, definition of abnormal FHR, assessments of uterine contractions)
Luthy et al, 1987, Canada and USA [28]	Compare the effect on neonatal outcome of EFM vs. IA	RCT 246 women with preterm singleton pregnancies with fetal weights 700–1750 g included (122 EFM. 124 IA)	Description of IA practices (device, frequency, timing, duration,definition of normal FHR, definition of abnormal FHR, assessments of uterine contractions)
Neldam et al, 1986, Denmark [29]	Compare the effect on maternal and neonatal outcome of EFM vs. IA	RCT 969 low- and high-risk women included (482 EFM, 487 IA)	Description of IA practices (device, frequency, timing, duration, definition of normal baseline, definition of abnormal FHR)
MacDonald et al, 1985, Ireland [4]	Compare the effect on maternal and neonatal outcome of EFM vs. IA	RCT 12964 women with a live fetus and gestational age ≥ 28 weeks were included (6474 EFM, 6490 IA)	Description of IA practices (devise, frequency, duration, definition of abnormal FHR)
Appelgate et al, 1979, USA [31] and Haverkamp et al, 1976, USA [33]	Compare the effect on maternal and neonatal outcome of EFM vs. IA	RCT 483 high-risk women included (242 EFM, 241 IA)	Description of IA practices (frequency, timing, duration, definition of normal baseline, definition of abnormal FHR)
Haverkamp et al, 1979, USA [32]	Compare the effect on maternal and neonatal outcome of EFM alone or EFM with option to FBS or IA	RCT 669 high-risk women included (220 EFM alone, 223 EFM with option to FBS, 226 IA)	Description of IA practices (frequency, timing, duration, definition abnormal FHR)
Kelso et al, 1978, UK [34]	Compare the effect on maternal and neonatal outcome of EFM vs. IA	RCT 504 low-risk women included (253 EFM, 251 IA)	Description of IA practices (device, frequency, timing, duration, definition normal baseline)
*Studies describing devices and modes of performing*, *and assessing the effect of different modes of IA*			
Kamala et al, 2018, Tanzania [15]	Compare the effect on maternal and neonatal outcome of IA with Doppler device vs. IA with Pinard	RCT 2844 women with cephalic presentation, gestational age ≥ 37 weeks and normal FHR at admission included (1421 Doppler, 1423 Pinard)	Description of IA practices (device, frequency, timing, definition of abnormal baseline). Effects of Doppler device vs. Pinard (detection of abnormal FHR, caesarean section, Apgar score < 7 at 5 min, bag mask ventilation attempted, admission to neonatal unit, fresh stillbirth, perinatal death, composite outcome)
Mdoe et al, 2018, Tanzania [16]	Compare the effect on maternal and neonatal outcome of IA with Doppler device vs. IA with Pinard	RCT 2684 women with cephalic presentation, gestational age ≥ 36 weeks and normal FHR at admission included (1309 Doppler device, 1375 Pinard)	Description of IA practices (device, definition of normal and abnormal baseline. Effects of Doppler device vs. Pinard (detection of abnormal FHR, time interval abnormal FHR to birth, caesarean section, bag mask ventilation, Apgar score <7 at 1 and 5 min, fresh stillbirth, early neonatal death, admitted to neonatal area, adverse perinatal outcome)
Byaruhanga et al, 2015, Uganda [18]	Compare the effect on maternal and neonatal outcome of IA with Doppler device vs. IA with Pinard	RCT 1971 women with a singleton pregnancy, in a cephalic position with gestational age > 37 weeks (992 Doppler device, 979 Pinard)	Description of IA practices (device, frequency, timing, duration, how FHR was counted, definition of normal baseline, definition of abnormal FHR, assessment of maternal pulse) Effects of Doppler device vs. Pinard (detection of abnormal FHR, Apgar score < 7 at 5 min, admission to special care unit, neonatal encephalopathy, caesarean section)
Mahomed et al, 1994, Zimbabwe [26]	Compare the effect on maternal and neonatal outcome of IA with Doppler device by a research midwife, Pinard by a research midwife, Pinard by midwife on duty or intermittent CTG	RCT 1255 high- and low-risk women with a singleton pregnancy, in a cephalic position, gestational age >37 weeks, singleton, cephalic present, with normal FHR at admission were included (312 Doppler device by research midwife, 310 Pinard by research midwife, 315 Pinard by midwife on duty, 318 intermittent CTG)	Description of IA practices (device, frequency, timing) Effects of Doppler device vs. Pinard (duration of labour, caesarean section, assisted vaginal delivery, spontaneous vaginal delivery, Apgar score <6 at 5 min, fits in neonatal unit, hypoxic encephalopathy, stillbirth or neonatal death)
*Studies assessing the accuracy of IA*			
Simpson et al, 1999, Canada [24]	Investigate if the accuracy of auscultation could be improved with the use of a heart rate meter	Accuracy study 15 experienced nurses and 15 obstetric residents were asked to assess six FHR recordings/traces by counting alone, counting with the help of a meter and visual assessment	Description of the accuracy of baseline variability, periodic changes and if the FHR pattern was assessed as reassuring or non-reassuring when counting alone and counting by the help of a meter
Strong & Jarles, 1992, USA [36]	Evaluate current practice of auscultation on the detection of decelerations	Accuracy study 120 nurses and physicians were asked to assess an intrapartum FHR recording containing a deceleration	Description of accuracy of baseline, deceleration nadir and deceleration duration
Miller et al, 1984, USA [30]	Define what characteristics of FHR and FHR patterns can be recognised by IA	Accuracy study 16 nurses and 16 physicians were asked to assess eight intrapartum FHR recordings containing a contraction	Descriptions of accuracy of baseline, accelerations without periodic change and non-uniform), saltatory pattern, decelerations (early, variable, late with good variability and late with diminished variability
Day et al, 1968, Australia [35]	Determine accuracy and usefulness of clinical measurement of the FHR	Accuracy study A trained midwife, a resident obstetrician and two medical students did clinical auscultations (126 observations in 90 women)	Descriptions of auscultation errors (random error, error biased towards normality, error based on inability to count during contractions)

**Table 2:** Characteristics and quality of included guidelines.

Organisation/country, year, guideline title, developed by	Description	Quality assessment
World Health Organization, 2018 [1] *Intrapartum care for a positive childbirth experience* World Health Organization	Guideline including 56 recommendations for intrapartum care. Recommendations 13 and 18 are about IA	• The guideline group included persons from all relevant areas/professions • Views and preferences of target population were sought • The target users of the guideline are clearly defined • Systematic methods were used to search for evidence • Criteria for selecting evidence are clearly described • Strengths and limitations of the body of evidence are clearly described • Methods for formulating recommendations are clearly described • The guideline were externally reviewed by experts before publication Overall quality assessment: good
International Confederation of Midwives (ICM), 2017 [2] *Use of Intermittent Auscultation for Assessment of Foetal Wellbeing during Labour* ICM	Position statement on use of IA	• Unclear who developed the statement • Views and preferences of target population have not been sought • The target users of the guideline are not defined • Unclear if systematic methods were used to search for evidence • Criteria for selecting evidence are unclear • Strengths and limitations of the body of evidence are not described • Methods for formulating recommendations are unclear • Unclear if the guideline were externally reviewed by experts before publication Overall quality assessment: unclear (Position statement, quality assessment meant for guidelines aimed to be evidence-based)
International Federation of Gynecology and Obstetrics (FIGO), 2015 [3] *FIGO consensus guidelines on intrapartum fetal monitoring* D. Lewis and S. Downe for the FIGO Intrapartum Fetal Monitoring Expert Consensus Panel	Consensus guideline describing devices for IA, discusses advantages and disadvantages of the devices, providing recommendations for when to continue IA in settings where CTG is available and techniques for how to perform IA	• The guideline development group included 2 midwives, the FIGO Intrapartum Fetal Monitoring Expert Panel of 45 obstetricians • Views and preferences of target population are partly described • The target users of the guideline are defined • Unclear if systematic methods were used to search for evidence • Criteria for selecting evidence are unclear • Strengths and limitations of the body of evidence are not described • Methods for formulating recommendations are unclear • The guideline were not externally reviewed by experts before publication Overall quality assessment: unclear (Consensus guideline, quality assessment meant for guidelines aimed to be evidence-based)
Denmark, 2017 [41] *Intrapartum fetal surveillance–indications* The Danish Society of Obstetricians and Gynaecology (DSOG)	Guideline providing recommendations on when to use IA, intermittent CTG and continuous CTG during intrapartum care	• The guideline development group included 2 midwives and 15 obstetricians • Views and preferences of target population were not sought • The target users of the guideline are not clearly defined • Systematic methods were used to search for evidence • Criteria for selecting evidence are partly described • Strengths and limitations of the body of evidence are not always clearly described • Methods for formulating the recommendations are clearly described • Unclear if the guideline were externally reviewed by experts before publication Overall quality assessment: moderate
Norway, 2014 [40] *Intrapartum fetal surveillance*, *cord clamping and cord-blood sampling for blood gas analyses* Norwegian Society of Gynecology and Obstetrics (NGF)	Guideline with recommendations for intrapartum fetal monitoring, cord clamping and cord-blood gas analyses	• The guideline development group included 1 midwife and 8 obstetricians • Views and preferences of target population were not sought • The target users of the guideline are clearly defined • Unclear to what degree systematic methods were used to search for evidence • Strengths and limitations of the body of evidence are not clearly described • Methods for formulating the recommendations are unclear • The guideline was not externally reviewed by experts before publication Overall quality assessment: unclear
Sweden, 2015 [42] *Fetal surveillance in active labour*. *Recommendations for clinical practice* The insurance company for Swedish regions (LØF)	Guideline for fetal monitoring during active labour	• The guideline development group included 1 midwife, 1 neonatologist, 3 obstetricians • Views and preferences of target population were not sought • The target users of the guideline are not defined • Unclear if systematic methods were used to search for evidence • Strengths and limitations of the body of evidence are not described • Methods for formulating recommendations are not described • Unclear if the guideline was externally reviewed by experts before publication Overall quality assessment: unclear
England and Wales, 2014 [39] *Intrapartum care*. *Care of healthy women and their babies during childbirth* National Institute of Clinical Excellence (NICE)	Guideline providing recommendations for intrapartum care. Recommendations Chapters 1.4., 1.10 and 1.13 include recommendations on IA Chapters 1.4 and 1.10 were updated in 2017	• The working group included 3 midwives, 3 obstetricians, 1 neonatologist, 1 obstetric anesthesiologist, 2 patient/carer/consumer representatives • Views and preferences of the target population were sought • The target users of the guideline are clearly defined • Systematic methods were used to search for evidence • Criteria for selecting evidence are clearly described • Strengths and limitations of the body of evidence are clearly described • Methods for formulating recommendations are clearly described • The guidelines were externally reviewed by stakeholders before publication Overall quality assessment: good
Canada, 2007, reaffirmed 2018 [43] *Fetal health surveillance*: *Antepartum and intrapartum consensus guideline* The Society of Obstetricians and Gynaecologists of Canada (SOGC) and British Columbia Perinatal Health Program.	Guideline for intrapartum fetal monitoring	• Principal authors were 2 medical doctors and 1 nurse; Consensus committee included 11 medical doctors, 1 midwife, 1 nurse, 1 unknown profession • Unclear if views and preferences of the target population were sought • The target users of the guideline are clearly defined • Unclear to what degree systematic methods were used to search for evidence • Criteria for selecting evidence are unclear • Strengths and limitations of the body of evidence are clearly described • Methods for formulating recommendations are clearly described • The guidelines were externally reviewed before publication Overall quality assessment: moderate (Consensus guideline, quality assessment meant for guidelines aimed to be evidence-based)
Australia and New Zealand, 2014 [44] *Intrapartum fetal surveillance*. *Clinical guideline*. The Royal Australian and New Zealand College of Obstetricians and Gynaecologists (RANZCOG)	Guideline for intrapartum fetal monitoring	• The guideline development group included 2 midwives, 8 obstetricians, 2 MFM specialist, 1 medical specialist, 3 RANZOG administrative staff. In addition, a NZ consultation group with 3 obstetricians, 2 midwives. • Views and preferences of the target population were not sought • The target users of the guideline are clearly defined • Systematic methods were used to search for evidence • Criteria for selecting evidence are clearly described • Strengths and limitations of the body of evidence are clearly described • Methods for formulating recommendations are clearly described • The guideline was not externally reviewed by experts before publication Overall quality assessment: moderate
USA, 2015 [45] *ACNM Clinical Bulletin*: *Intermittent auscultation for intrapartum fetal heart rate surveillance* American College of Nurse-Midwives (ACNM)	Guideline for IA during childbirth	• The guideline development group included 6 midwives • Views and preferences of the target population have been sought • The target users of the guideline are not defined • Unclear if systematic methods were used to search for evidence • Criteria for selecting the evidence are unclear • Strengths and limitations of the body of evidence are clearly described • Methods for formulating the recommendations are clearly described • Unclear if the guideline was externally reviewed before publication Overall quality assessment: moderate
USA, 2018 [46] *AWHONN position statement*: *Fetal heart monitoring* Association of Women’s Health, Obstetric and Neonatal Nurses (AWHONN)	Position statement for fetal heart monitoring during childbirth	• The guideline development group is not described • The views and preferences of the target population were not sought • The target users of the guideline are clearly defined • Unclear if systematic methods were used to search for evidence • Criteria for selecting the evidence are unclear • Strengths and limitations of the body of evidence are not described • The methods for formulating the recommendations are unclear • Unclear if the guidelines were externally reviewed before publication Overall quality assessment: unclear (Position statement, quality assessment meant for guidelines aimed to be evidence-based)

**Fig 1:**
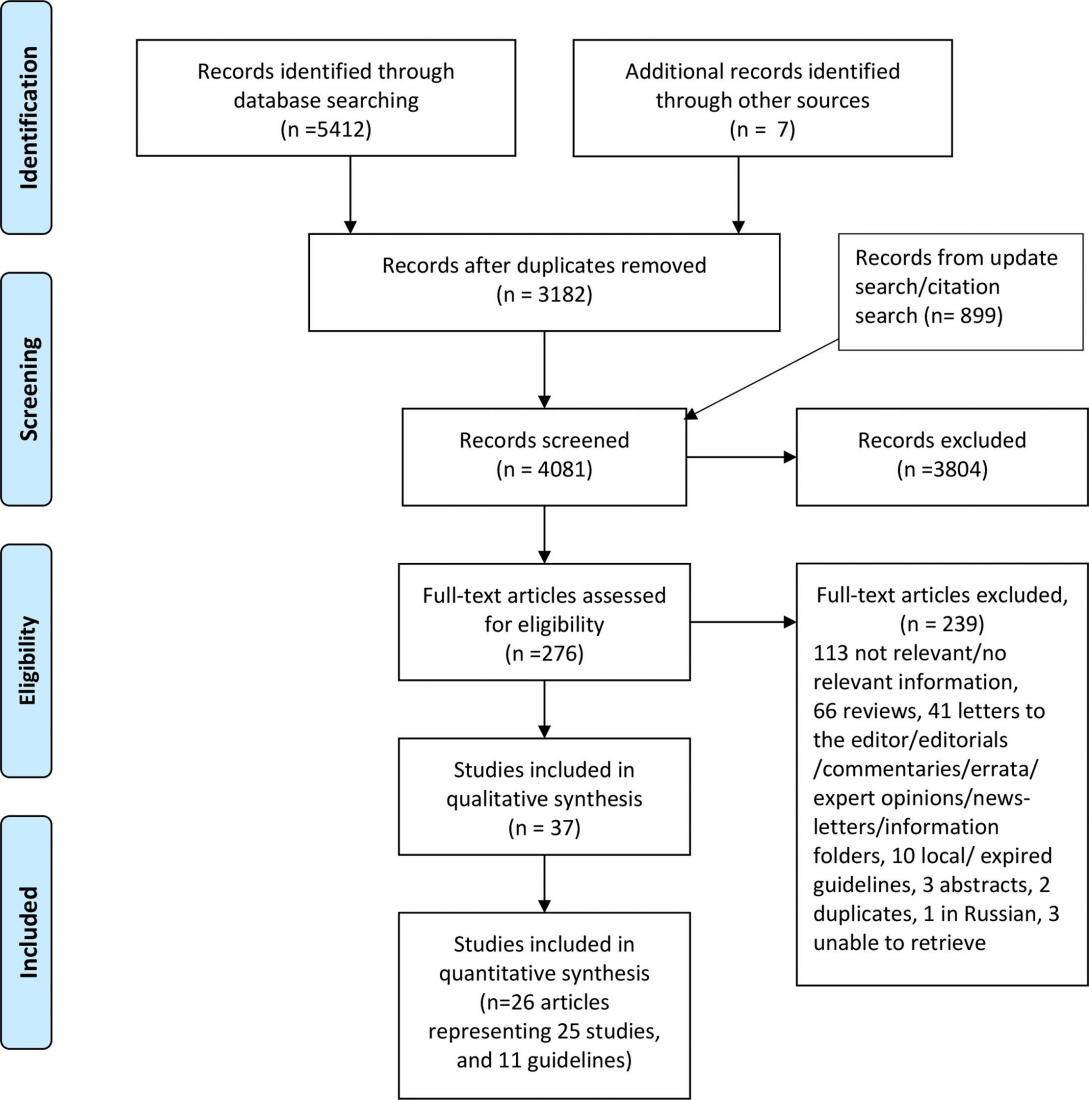
PRISMA flow diagram of included studies.

Fig 1 gives an overview of the inclusion process, and the S2 Table lists the papers excluded after full-text assessment and the reason for exclusion.

### Findings from the studies

Five of the 26 studies were from African countries [15, 16, 18, 26, 37], two from India [19, 21], one from the Middle East [20], seven from Europe [4, 22, 23, 27, 29, 34, 38], seven from North America [24, 28, 30–33, 36] and four were from Australia/New Zealand [9, 17, 25, 35]. The date range was from 1968 to 2019 (Table 1). Among the 26 articles, 25 were original studies (Table 1). Altogether 17 studies (in 18 articles) [4, 9, 17, 19–23, 25, 27–29, 31–34, 37, 38] provided descriptions of devices and techniques for performing IA, one of which also presented and evaluated a decision-making framework for fetal heart monitoring in low-risk women [9] (S1 Fig). Four were randomized trials comparing the effects of IA using Doppler vs. Pinard devices and provided descriptions of the devices and modes [15, 16, 18, 26]. Four studies assessed the accuracy of IA [24, 30, 35, 36] (Table 1).

#### Devices used for IA

The Pinard fetoscope, DeLee fetoscope, and Doppler device (battery or wind-up) were the common tools used to perform IA. Some studies did not specify the device [21, 22, 25, 31–33, 35], and two studies just called it “a stethoscope” [19, 29]. There were no detailed descriptions of Pinard or DeLee fetoscopes in the studies in which they were used [4, 9, 15–18, 26, 28, 34, 38]. Seven studies provided information of the type of the Doppler or electronic device [15, 16, 18, 26, 27, 34, 37] (S3 Table). One study [20] described a new intervention, an electronic stethoscope (the stethoscope was attached to a hand-held computer). The authors showed examples–one with a stethoscope attached to a cell phone and the other a Pinard device attached to an MP3 player. According to the authors, in addition to real-time fetal heart sound analyses, the device could create and save images of the sounds.

#### Frequency, timing, and duration of auscultation

The studies described slightly different techniques for performing IA. Some did not provide detailed descriptions. The S3 Table provides a detailed overview of each study.

During the first stage of labour, the frequency of auscultation was described as either every 15 min [4, 19, 21, 27, 28, 31–34, 38], 15–30 min [9, 17] or 30 min [15, 16, 18, 37]. One study reported the frequency as every 30 min in low-risk women and every 15–30 min in women at higher risk [25]. Another study reported IA frequency at every 30 min until 5-cm cervical dilatation and then every 15 min [29]. Timing of auscultation was described as either after a contraction [4, 18, 19, 22, 31–33, 37, 38], during and after a contraction [21, 23, 26, 34], between and after contractions [28], and between, during, and after contractions [9, 17, 27]. One study reported auscultation during fetal movement as part of a baseline assessment [17]. Duration of each FHR counting varied from 15 to 60 s (S3 Table).

During the second stage of labour, the most common frequencies of auscultation were every 5 min or after each contraction [9, 17, 19, 21, 22, 27, 28, 31–33, 38]. The most common frequencies of auscultation were every 5 min or after each contraction [9, 17–19, 21, 22, 25, 27, 28, 31–33]. One study reported a frequency of every 15 min [15], and two every 5–15 min [16, 37]. One study reported the frequency as every 15 min in low-risk women and after most contractions in those at higher risk [25]. Most studies had similar recommendations for the timing of auscultation for the first and second stages (S3 Table).

The most common duration of each auscultation was a full minute but varied from 15 to at least 60 s. Two of the studies reported that auscultations were performed for periods of 10 min [15, 26]. We understood this to mean several frequent auscultations rather than one reading that lasted 10 min.

#### Observations during auscultation

The following observations were reported: baseline or average FHR [4, 9, 15, 16, 18, 19, 25, 27–29, 31, 32, 34]; the presence or absence of FHR decreases (decelerations) and increases (accelerations) [9, 17–19, 26–29, 31–33]; FHR rhythm [9, 18]; maternal pulse [9, 17]; frequency and duration of contractions [9, 17]; uterine activity and tone [9, 17, 27]; fetal movements [9, 17] (S3 Table).

#### Definitions of normal and abnormal FHR

Few of the included studies provided detailed descriptions or definitions of normal and abnormal IA findings. Normal FHR baseline was described as 110–160 beats per minute (bpm) [9, 17, 18, 38] or 120–160 bpm [16, 27–29, 31–34, 37]. One study described normal auscultation findings as follows: average FHR 110–160 bpm, FHR increases of ≥15 bpm (accelerations), absence of FHR decreases (decelerations), regular rhythm–all in combination with normal uterine activity and tone [9].

Abnormal findings were most commonly described as “below or above the normal baseline” but also as the presence of deceleration and the absence of acceleration. The S3 Table provides a more detailed description of definitions of normal and abnormal FHR.

### Findings from the guidelines

The WHO international guidelines [1] and the England and Wales NICE guidelines [39] were found to be of good quality, meaning that they established quality criteria. This included the requirement that relevant professions were involved in the guideline development group, that views and preferences of service users were sought and that systematic methods were used to search for the evidence. The rest of the included guidelines were assessed as either of moderate or unclear quality (Table 2). Three guidelines of unclear quality were either position statements or consensus guidelines and did not claim to be evidence based [2, 3, 54]. In five of the seven national guidelines, obstetricians or medical doctors comprised an absolute majority of the guideline development groups [40–44]. One guideline was developed by midwives only [45]. Only three of the 11 guidelines stated clearly that users were represented in the guideline development group or that their views and preferences were sought in other ways [1, 39, 45]. Three guidelines stated clearly that systematic methods were used to search for the evidence [1, 39, 42].

The Swedish guideline recommended admission CTG for all women, and intermittent CTG or IA for low-risk women [42]. We assessed the Swedish guidelines as being of unclear quality (Table 3). The Canadian guideline was updated the most recently in 2007 [47], and was reaffirmed in 2018, with no updates or changes [43]. None of the other guidelines had been established before 2014 (Table 2).

**Table 3:** Outcome events and meta-analyses.

Outcome	No. of studies	Events, n/N Doppler	Events, n/N Pinard	Effect size (95% CI)^1^	I^2^ (%)
Apgar score <7 at five minutes^2^	4	66/4034	63/4402	1.12 (0.65–1.93)	49
Caesarean section	4	696/4034	704/4402	1.16 (0.91–1.49)	84
Composite neonatal outcome^3^	2	60/2730	63/2798	0.98 (0.69–1.39)	0
Stillbirth and early neonatal death^4^	4	31/4034	39/4402	0.92 (0.44–1.91)	48
Assisted vaginal delivery	1	104/312	78/625	0.72 (0.48–1.08)	-
Detection of abnormal FHR	4	327/4034	226/4402	1.77 (1.29–2.43)	74

^1^Random effect model.

^2^One study measured Apgar score <6 at five minutes.

^3^Fresh stillbirth, early neonatal death < 24 h and admission to Neonatal Intensive care unit. Slightly different definitions in the two studies.

^4^Slightly different definitions across the studies.

The FIGO guideline recommended IA for fetal monitoring in all settings if CTG is not available, and stated that IA may be used in settings where CTG is available [3]. The Swedish guidelines recommended, for low-risk women, IA or intermittent CTG (20–30 min every second hour with IA in between) during the first stage of labour and IA or continuous CTG during the second stage of labour [42]. The remaining nine guidelines recommended IA, but not CTG, in low-risk women. Whereas four of the guidelines explicitly stated that admission CTG was not recommended in low-risk women [1, 39, 40, 43], the Swedish guideline recommended admission CTG for all women [42]. The remaining six guidelines did not provide any recommendation on fetal monitoring on admission. The S4 Table provides detailed information about the recommendations from each guideline.

#### Recommended devices for IA

Three of the guidelines did not provide recommendations on the device to be used for IA

[42, 44, 46]. The others recommended both the Doppler device and the Pinard or DeLee fetoscope.

#### Recommended frequency, timing and duration of auscultation

During the first stage of labour, most guidelines recommended a frequency of every 15–30 min [1, 40–46]. Two guidelines recommended a frequency of 15-min intervals [3, 39], while one offered no recommendations regarding the frequency of auscultation [2].

During the second stage of labour, one guideline recommended a frequency of every 5–15 min [46], two offered no recommendation [2, 39], and the remaining eight guidelines recommended auscultation every 5 min or after each contraction.

One of the guidelines recommended that the frequency of auscultation should be individualised to the contraction pattern, maternal activity, and interventions that may affect the FHR. The same guideline also recommended listening between contractions to assess the FHR baseline [45].

Six guidelines recommended that auscultation should be performed during and after a contraction [1, 3, 40, 41, 44, 45], two immediately after a contraction [39, 42], and one between contractions [43]. Two guidelines did not describe the timing of auscultation [2, 46]. Three of the guidelines recommended auscultating over three contractions if the FHR was not always in the normal range [1, 39, 41]. Among the guidelines recommending auscultation for at least 60 s, one recommended 15–60 s [43] and another 30–60 seconds [45]. Two others did not describe the duration of auscultation [2, 46].

Some of the guidelines provided detailed descriptions on how to count and observe the FHR, whereas others did not. Most guidelines recommended documentation of the FHR as a single count (in bpm). Others simply stated that the FHR rhythm and presence or absence of decelerations and accelerations should be documented. The S4 Table describes in detail the recommendations in each guideline.

#### Recommended observations during auscultation

Some of the guidelines recommended observation of the frequency or pattern of contractions [3, 41–43, 45], fetal movements [3, 39], and maternal pulse to differentiate it from the FHR [3, 39, 42, 43].

#### Definitions of normal and abnormal FHR

Most guidelines did not provide detailed descriptions of normal and abnormal auscultation findings, except for the FHR baseline. Denmark and Norway guidelines defined the normal baseline as 110–150 bpm [40, 41], whereas all other guidelines defined it as 110–160 bpm. One guideline provided a systematic classification of normal and abnormal auscultation findings [45]. Its normal findings included a baseline of 110–160 bpm, regular rhythm, and the absence of FHR decreases or decelerations from the baseline. Abnormal findings included any of the following: irregular rhythm, presence of FHR decreases or decelerations from the baseline, tachycardia (>160 bpm for >10 min), or bradycardia (<110 bpm for >10 min) [45].

### Effects of various IA modes

Four studies were included in a meta-analysis comparing the effects of IA performed with a Pinard vs. a Doppler device [15, 16, 18, 26]. The methods of the meta-analysis, risk-of-bias assessments and sensitivity analyses are described in detail in S1 Text. The studies were from Uganda, Tanzania and Zimbabwe and included 8436 women and their babies. An Apgar score <7 after five minutes and caesarean sections were the primary endpoints. Secondary endpoints were a composite neonatal outcome (fresh stillbirth, early neonatal death at <24 h, admission to the neonatal intensive care unit) and the following single-outcome measures: stillbirth/neonatal death, assisted vaginal delivery, detection of an abnormal FHR.

Among the women randomized to IA by different devices, abnormal FHR was detected more often using the Doppler device than with the Pinard device [risk ratio 1.77, 95% confidence interval 1.29–2.43]. The difference, however, did not affect the clinical outcomes, as there were no significant differences in any of the other outcomes (Table 3).

For the sensitivity analyses, one of the studies [26] was excluded, resulting in lower heterogeneity, but it did not affect the results significantly (S1 Text). The GRADE assessment of the overall quality of evidence found low confidence in the effect estimates, except for stillbirth and neonatal death, which were assessed as moderate (S1 Text).

### Accuracy of IA

We identified four studies that assessed the accuracy of auscultation [24, 30, 35, 36]. Three of the studies were performed in classroom settings, where the observers listened to recorded soundtracks [24, 30, 36] and one in a clinical setting where the observers auscultated labouring women [35]. CTG signals recorded simultaneously were regarded as the gold standard comparator. One study [24] found that counting alone was associated with underestimation of the FHR. When a heart rate meter was displayed together with the soundtrack (i.e., the FH was displayed on screen), the assessments were significantly more accurate, and the intra-observer variation decreased.

Another study investigated the characteristics of FHR and FHR patterns that could be recognised by IA [30]. One-third of the observers failed to identify periodic FH patterns such as decelerations and a saltatory pattern (wide, rapid oscillations of the FHR). Baseline was the most likely to be scored correctly, and the saltatory pattern was the most difficult. The third study [35] investigated the accuracy of clinical auscultation of FHR. The authors described three types of auscultation errors: random error; error biased toward normality when the FHR was fast or slow; error based on inability to count FHR during contractions. In the fourth study [36], a recording of intrapartum FHR was played to 120 physicians and nurses. They were asked to estimate the baseline and then the duration and nadir of a deceleration. Mean estimates were not significantly different from the actual values, but individual assessments varied widely.

## Discussion

### Summary of evidence

We identified different protocols for frequency, timing and duration of IA. Studies assessing FHR auscultation against a “reference” or “gold standard” described deviations in interpretations. The Doppler device and the Pinard fetoscope were the most commonly reported devices for undertaking IA. We carried out a meta-analysis assessing IA performed by a Doppler device vs. Pinard device and found no difference in clinical outcomes. The quality of the included guidelines varied.

We are aware that the transducer from the CTG machine is often used for IA. We were, however, not able to identify any studies that described this practice, and it was not mentioned in any of the recommendations. Martis et al. mention the practice in their Cochrane systematic review, and point to that most CTG machines have autocorrelation that frequently averages the FHR, and that this is not the same as counting FHR for a full minute or other recommended periods of time [48].

We were not able to identify any trials that assessed the optimal timing or duration for auscultations on neonatal and maternal outcomes. Sholapurkar claimed, based on literature reviews and clinical cases, that auscultation conducted after a contraction can result in missing late decelerations. He recommended auscultation 30–60 s before and after contractions to assess the baseline FHR and late decelerations [49].

There is no research to guide the optimal techniques for and timing of IA assessment of fetal heart activity, and most of the guideline recommendations were based on the techniques described in controlled trials that compared the effects of the Doppler vs. Pinard devices, most of them from Anglo-Saxon countries. Based on historical anecdotes, the frequency of auscultation in most European countries was variable (in some cases less than hourly and often every 30 min or so during the first stage of labour). Most routine clinical practice changed to every 15 min, however, following publication of the Dublin trial in 1985 [5]. Nevertheless, a controlled study has never been undertaken to compare the outcomes of the various frequency intervals.

The definitions have changed over time, with the normal FHR baseline being defined as 120–160 bpm in older studies [27–29, 31, 32] but also in the three contemporary studies from Tanzania [15, 16, 37]. In 1987, FIGO accepted 110–150 bpm as the normal baseline [50], which was revised to 110–160 bpm in their 2015 consensus guidelines [3, 51]. National guidelines from Denmark and Norway define the normal baseline as 110–150 bpm [40, 41], whereas other guidelines define normal as 110–160 bpm. There is resistance to implementing the 2015 FIGO recommendations for intrapartum fetal monitoring in Norway and Denmark, the main reasons being that the 2015 FIGO guidelines are not regarded as evidence based [52, 53]. CTG combined with ST waveform analysis of the fetal ECG is widespread in the two countries, and the algorithm for using the ST waveform analysis equipment depends on the 1987 FIGO guidelines [50].

Midwives and doctors who traditionally auscultated using a Pinard device assessed and described the findings using other senses and words than when interpreting a CTG tracing. They described the characteristics of the fetal heart sound as “timbre,” “strength,” and “rhythm”. A 1972 textbook on obstetrics states that “skilled maternity carers will be able to discriminate the normal timbre from the abnormal” [54]. These descriptive, and possibly clinically significant, words and expressions disappeared from the formal clinical record after CTG usage became widespread, and auscultation findings are now described using the same words and expressions as when describing CTG tracings. Only two of the articles [9, 18] and two guidelines [43, 45] mentioned rhythm in auscultation assessments.

One study provided a decision-making framework for how to assess and perform fetal monitoring of low-risk women (S1 Fig). The framework was developed, as well as implemented and evaluated, in New Zealand. After implementation, the rate of performing IA increased, with fewer low-risk women being monitored with CTG [9].

Only two of the 11 included guidelines were assessed as of good quality (Table 3). The main weakness was that all professions that provide, and women who receive, maternity care had little or no influence in the working groups that made the guidelines. Medical doctors, many of them not skilled in IA, usually dominated the guideline working groups, which may be have contributed to superficial descriptions of IA methods and FHR characteristics.

The four studies evaluating accuracy, [24, 30, 35, 36], found that individual assessments could deviate from the values documented on CTG tracings. This discrepancy is possibly because IA allows practitioners to hear fetal sounds directly, and thus the examiner is being exposed to information that is different from that recorded by CTG technology. Therefore, it is probably not easy to define and assess fetal heart sounds uniformly by either auscultation or CTG. Indeed, studies evaluating how clinicians interpreted CTG tracings found poor interrater agreement [55, 56].

Although IA is the recommended method for intrapartum fetal monitoring, few trials have evaluated its use during the past two and a half decades. Five trials have assessed IA vs. 20–30 min CTG upon admission to the labour ward [22, 23, 38, 57, 58]. All five trials–four of them in a meta-analysis [59]–concluded that admission CTG does not improve neonatal or maternal outcomes.

Recently, three trials assessed the effect of IA performed by Doppler vs. Pinard devices [15, 16, 18]. We included them, together with a previous trial [26], in a meta-analysis and found no difference in outcomes in women and babies randomised to a Doppler device vs. a Pinard device. As all four trials were performed in low-income countries, the comparison should be repeated in different settings to enable maximum external generalisability. The GRADE assessment of the quality of evidence suggests that the findings for stillbirth and neonatal death were of moderate quality, but those for other outcomes were less robust, In addition, it found that the direction of effect and the effect size could change in the future if new studies are included in the meta-analyses (S1 Text).

The Pinard is produced in different sizes and from different materials. It is possible that size and material influence on the quality of auscultation. None of the studies described the design or material of the Pinard model used. Doppler devices and CTG machines were often described by model.

### Limitations

Our scoping review has strengths and limitations. We published the study protocol before we initiated the literature searches [10]. We did broad literature searches, and it is unlikely that we missed significant publications. The searches were not limited by language restrictions, although we realised that we had limited resources for translation. Hence, for the analyses, we included only literature in languages with which the study group was confident about understanding. The searches were limited to scientific literature and guidelines. Including literature such as textbooks and essays probably would have resulted in more descriptions of IA techniques and protocols. Deviations from our protocol are described in S3 Text.

## Conclusions

### Implications for future research

No trials have been published that compared protocols for performing IA. In the future, if such trials are performed to assess perinatal morbidity and mortality, iatrogenic intervention, and the women’s and practitioners’ views and experiences, they must include large numbers of women to gain sufficient power because serious adverse outcomes seldom occur in low-risk women. Because the current evidence suggests that routine (vs. selective) use of CTG does not benefit women and babies at high-risk and is disadvantageous for those at low-risk, an”all-risk’ trial might be helpful. Another challenge is that the model of care, like e.g., continuity of care, one-to-one midwifery care, must be considered in such trials.

There is a need to investigate the practice and potential use of IA by performing studies assessing interrater agreement and to what degree clinicians can detect and describe different FHR patterns. The skills needed to perform IA using the Pinard device are being lost among today’s midwives and obstetricians and have more-or-less disappeared in many settings [60, 61]. This means that practitioners will not have the skills to assess fetal well-being in situations where more advanced tethe machinery is unavailable for any reason, or where they are attending women in settings where there is no access to CTG or Doppler monitoring.

The benefits of IA, especially with the Pinard device, are increasingly unavailable to women. Such benefits include the midwife or obstetrician being in direct contact with the woman herself, which would allow them to observe the range of physiological and behavioural cues from the woman in conjunction with the aural fetal heart and uterine sounds. Being in close proximity also promotes personal engagement with the woman, increasing the chance of building a supportive relationship. This situation is less likely to happen if the fetal heart assessment technique creates a physical distance from the woman’s body and the care provider, as is the case to a lesser extent with a Doppler device and to a much greater extent with a CTG machine, especially if the latter is wireless and the signal is being read away from the woman herself, at a staff station or even in another building.

Innovation, leading to new devices for IA, and in signal processing and analysis improving accuracy, could be useful. The Pinard device is reported to be difficult to use in some maternal positions, and Doppler devices expose the fetus to ultrasound waves [48]. We identified only one study that had developed an electronic stethoscope for intermittent fetal monitoring [20].

### Implications for practice

There is a need to improve the quality of guideline recommendations by involving user representatives and midwives and ensuring that the best evidence is sought and used. Our meta-analysis found that IA performed with a Doppler device was associated with the detection of more abnormal FHR patterns. Such detection, however, did not lead to better outcomes for the babies, nor did it lead to more interventions in the mothers. Currently, there is no evidence to recommend the Doppler over the Pinard device, or vice versa.

## Supporting information

S1 Table Literature search history. A detailed description of the electronic literature searches.
(DOCX)

S2 Table Articles excluded after full text assessment. A list of the 238 articles that were excluded after full-text assessment and the reason for exclusion.
(DOCX)

S3 Table Detailed descriptions of devices and modes of performing IA from the included articles. A detailed description of devices used; the frequency, timing, and duration of IA; definitions of normal and abnormal FHRs; and additional observations performed together with auscultation.
(DOCX)

S4 Table Detailed descriptions of recommendations from the included guidelines. A detailed description of recommendations of devices; frequency, timing, and duration of IA; definitions of normal and abnormal FHRs; and additional observations performed together with auscultation.
(DOCX)

S1 Fig Decision making framework for fetal heart monitoring in low-risk women. Figures suggesting decision making on fetal heart monitoring at admission or first contact and during active labour.
(DOCX)

S1 Text Effects of IA performed with a Doppler device vs. a Pinard stethoscope. A detailed description of the methods and results of meta-analyses and GRADE assessment of the body of evidence.
Table 1. Risk of bias assessment
Table 2. Meta-analyses with forest plots and sensitivity analyses
Table 3. GRADE assessment of the overall quality of evidence (“Summary of findings”).
(DOCX)

S2 Text Protocol. The file contains the full protocol. As Prospero does not allow publication of systematic scoping review protocols, we published the protocol at Open Science Framework, 04.03.2017. DOI 10.17605/OSF.IO/KFT6K | ARK c7605/osf.io/kft6k.
(DOCX)

S3 Text Deviations from the protocol. During the study, we found it appropriate to change some of our plans. All deviations from the original protocol are described in the file.
(DOCX)
