# Supplementary material for: Intermittent auscultation fetal monitoring during labour: A systematic scoping review to identify methods, effects, and accuracy
Source: PLoS One. 2019 Jul 10;14(7):e0219573. doi: 10.1371/journal.pone.0219573 (PMC6619817; doi:10.1371/journal.pone.0219573)
Supplement: S1 Table — A detailed description of the electronic literature searches. (DOCX) [file pone.0219573.s001.docx]

**S1 Table. Literature search history**

**Database:** Ovid MEDLINE(R) Epub Ahead of Print, In-Process & Other Non-Indexed Citations, Ovid MEDLINE(R) Daily and Ovid MEDLINE(R) 1946 to Present

**Date:**  15.03.2017

**Results:** 1115

**Results after update of searches 05.01.2019**: 1190

| # | Searches | Results |
| --- | --- | --- |
| 1 | Labor, Obstetric/ | 28286 |
| 2 | Labor Onset/ | 868 |
| 3 | Labor Stage, First/ | 1244 |
| 4 | Labor Stage, Second/ | 1231 |
| 5 | Delivery, Obstetric/ | 25725 |
| 6 | Natural Childbirth/ | 2331 |
| 7 | Parturition/ | 6952 |
| 8 | Obstetric Labor Complications/ | 16184 |
| 9 | Pregnancy/ | 804207 |
| 10 | Gravidity/ | 1049 |
| 11 | Pregnancy, High-Risk/ | 4505 |
| 12 | Prenatal Care/ | 23467 |
| 13 | Prenatal Diagnosis/ | 34162 |
| 14 | Perinatal Care/ | 3583 |
| 15 | Fetus/ | 75098 |
| 16 | Fetal Heart/ | 8314 |
| 17 | Heart Rate, Fetal/ | 4675 |
| 18 | Fetal Monitoring/ | 6902 |
| 19 | (fetal or foetal or fetus* or foetus*).tw,kw,kf. | 287048 |
| 20 | (labor or laboring or labour or labouring).tw,kw,kf. | 95517 |
| 21 | pregnan*.tw,kw,kf. | 454478 |
| 22 | (childbirth* or child birth*).tw,kw,kf. | 15090 |
| 23 | parturition*.tw,kw,kf. | 13314 |
| 24 | intrapartum.tw,kw,kf. | 7419 |
| 25 | (antenatal* or prenatal* or perinatal*).tw,kw,kf. | 165370 |
| 26 | (obstetric* adj2 deliver*).tw,kw,kf. | 1162 |
| 27 | or/1-26 | 1108352 |
| 28 | Auscultation/ | 3952 |
| 29 | Heart Auscultation/ | 5133 |
| 30 | stethoscopes/ | 678 |
| 31 | fetoscopes/ | 112 |
| 32 | auscultat*.tw,kw,kf. | 5768 |
| 33 | ((handheld or hand held) adj3 (doppler or ultrasound* or transducer*)).tw,kw,kf. | 538 |
| 34 | (pinard* or pinnard*).tw,kw,kf. | 32 |
| 35 | la?nnec*.tw,kw,kf. | 658 |
| 36 | isia.tw,kw,kf. | 103 |
| 37 | delee hillis.tw,kw,kf. | 0 |
| 38 | (sonicaid* or sonic aid*).tw,kw,kf. | 63 |
| 39 | fetoscope*.tw,kw,kf. | 75 |
| 40 | (intermittent adj2 monitor*).tw,kw,kf. | 234 |
| 41 | or/28-40 | 14203 |
| 42 | 27 and 41 | 956 |
| 43 | ((f?etal monitoring or cardiotocograph* or ctg or f?etal heart rate* or fhr) adj3 intermittent).tw,kw,kf. | 67 |
| 44 | ((obstetric or f?etal) adj5 stethoscope*).tw,kw,kf. | 8 |
| 45 | 42 or 43 or 44 | 975 |
| 46 | limit 18 to (guideline or meta analysis or practice guideline or systematic reviews) | 156 |
| 47 | 45 or 46 | 1115 |

Comment: In Medline a supplemental search on «fetal monitoring» (line 46) limited to, guideline, meta-analysis, practice guideline and systematic review has been conducted.

**Database:** Embase 1974 to 2017 Week 11

**Date:**  15.03.2017

**Results:** 1216

**Results after update of searches 05.01.2019:** 1233

| # | Searches | Results |
| --- | --- | --- |
| 1 | labor/ | 27585 |
| 2 | labor onset/ | 2476 |
| 3 | labor stage/ | 506 |
| 4 | labor stage 1/ | 1139 |
| 5 | labor stage 2/ | 2005 |
| 6 | labor management/ | 1201 |
| 7 | obstetric delivery/ | 2126 |
| 8 | childbirth/ | 19347 |
| 9 | labor complication/ | 9246 |
| 10 | intrapartum care/ | 1697 |
| 11 | pregnancy/ | 681470 |
| 12 | high risk pregnancy/ | 10351 |
| 13 | prenatal care/ | 34321 |
| 14 | prenatal diagnosis/ | 55141 |
| 15 | perinatal care/ | 13329 |
| 16 | fetus/ | 198744 |
| 17 | fetus heart/ | 6867 |
| 18 | fetus heart rate/ | 9653 |
| 19 | fetus monitoring/ | 10813 |
| 20 | (fetal or foetal or fetus* or foetus*).tw,kw. | 347780 |
| 21 | (labor or laboring or labour or labouring).tw,kw. | 106143 |
| 22 | pregnan*.tw,kw. | 545074 |
| 23 | (childbirth* or child birth*).tw,kw. | 17741 |
| 24 | parturition*.tw,kw. | 14926 |
| 25 | intrapartum.tw,kw. | 9569 |
| 26 | (antenatal* or prenatal* or perinatal*).tw,kw. | 211888 |
| 27 | (obstetric* adj2 deliver*).tw,kw. | 1215 |
| 28 | or/1-27 | 1182481 |
| 29 | auscultation/ | 6497 |
| 30 | heart auscultation/ | 5271 |
| 31 | stethoscope/ | 1844 |
| 32 | fetal stethoscope/ | 11 |
| 33 | auscultat*.tw,kw. | 7366 |
| 34 | ((handheld or hand held) adj3 (doppler or ultrasound* or transducer*)).tw,kw. | 725 |
| 35 | (pinard* or pinnard*).tw,kw. | 59 |
| 36 | la?nnec*.tw,kw. | 615 |
| 37 | isia.tw,kw. | 107 |
| 38 | delee hillis.tw,kw. | 0 |
| 39 | (sonicaid* or sonic aid*).tw,kw. | 77 |
| 40 | fetoscope*.tw,kw. | 109 |
| 41 | (intermittent adj2 monitor*).tw,kw. | 354 |
| 42 | or/29-41 | 17787 |
| 43 | 28 and 42 | 1191 |
| 44 | ((f?etal monitoring or cardiotocograph* or ctg or f?etal heart rate* or fhr) adj3 intermittent).tw,kw. | 81 |
| 45 | ((obstetric or f?etal) adj5 stethoscope*).tw,kw. | 18 |
| 46 | 43 or 44 or 45 | 1216 |

**Database:** Cinahl with full text EBSCOhost

**Date:**  15.03.2017

**Results:** 511

**Results after update of searches 05.01.2019:** 691

| # | Query | Results |
| --- | --- | --- |
| S1 | (MH "Labor") | 3,236 |
| S2 | (MH "Labor Stages") | 185 |
| S3 | (MH "Labor Stage, First") | 307 |
| S4 | (MH "Labor Stage, Second") | 566 |
| S5 | (MH "Delivery, Obstetric") | 4,288 |
| S6 | (MH "Management of Labor") | 1,416 |
| S7 | (MH "Childbirth") | 6,232 |
| S8 | (MH "Labor Complications") | 1,76 |
| S9 | (MH "Pregnancy") | 103,88 |
| S10 | (MH "Pregnancy, High Risk") | 1,36 |
| S11 | (MH "Intrapartum Care") | 1,272 |
| S12 | (MH "Prenatal Care") | 8,905 |
| S13 | (MH "Perinatal Care") | 2,145 |
| S14 | (MH "Prenatal Diagnosis") | 3,881 |
| S15 | (MH "Obstetric Care") | 4,723 |
| S16 | (MH "Fetus") | 14,321 |
| S17 | (MH "Fetal Heart") | 281 |
| S18 | (MH "Heart Rate, Fetal") | 967 |
| S19 | (MH "Fetal Monitoring") | 1,044 |
| S20 | (MH "Home Fetal Monitoring") | 50 |
| S21 | TI ( (fetal or foetal or fetus* or foetus*) ) OR AB ( (fetal or foetal or fetus* or foetus*) ) OR MW ( (fetal or foetal or fetus* or foetus*) ) | 33,476 |
| S22 | TI ( (labor or laboring or labour or labouring or childbirth* or child birth* or pregnan* or parturition* ) ) OR AB ( (labor or laboring or labour or labouring or childbirth* or child birth* or pregnan* or parturition* ) ) OR MW ( (labor or laboring or labour or labouring or childbirth* or child birth* or pregnan* or parturition* ) ) | 140,545 |
| S23 | TI ( (intrapartum or antenatal* or prenatal* or perinatal*) ) OR AB ( (intrapartum or antenatal* or prenatal* or perinatal*) ) OR MW ( (intrapartum or antenatal* or prenatal* or perinatal*) ) | 39,84 |
| S24 | TI (deliver* N2 obstetric*) OR AB (deliver* N2 obstetric*) OR MW (deliver* N2 obstetric*) | 4,469 |
| S25 | (MH "Auscultation") | 734 |
| S26 | (MH "Heart Auscultation") | 247 |
| S27 | (MH "Stethoscopes") | 319 |
| S28 | TX auscultat* | 4,297 |
| S29 | TI ( (pinard* or pinnard*) ) OR AB ( (pinard* or pinnard*) ) OR MW ( (pinard* or pinnard*) ) | 23 |
| S30 | TX delee hillis | 3 |
| S31 | TI laennec* OR AB laennec* OR MW laennec* | 13 |
| S32 | TX fetoscope* | 65 |
| S33 | TX isia | 28 |
| S34 | TI ( ((handheld or hand held) N3 (doppler or ultrasound* or transducer*)) ) OR AB ( ((handheld or hand held) N3 (doppler or ultrasound* or transducer*)) ) OR MW ( ((handheld or hand held) N3 (doppler or ultrasound* or transducer*)) ) | 102 |
| S35 | TI (intermittent N2 monitor*) OR AB (intermittent N2 monitor*) OR MW (intermittent N2 monitor*) | 62 |
| S36 | TX (sonicaid* or sonic aid*) | 83 |
| S37 | S25 OR S26 OR S27 OR S28 OR S29 OR S30 OR S31 OR S32 OR S33 OR S34 OR S35 OR S36 | 4,806 |
| S38 | TI ( ((f#etal monitoring or cardiotocograph* or ctg or f#etal heart rate* or fhr) N3 intermittent) ) OR AB ( ((f#etal monitoring or cardiotocograph* or ctg or f#etal heart rate* or fhr) N3 intermittent) ) OR MW ( ((f#etal monitoring or cardiotocograph* or ctg or f#etal heart rate* or fhr) N3 intermittent) ) | 33 |
| S39 | TX (obstetric or f#etal) N5 stethoscope* | 46 |
| S40 | S1 OR S2 OR S3 OR S4 OR S5 OR S6 OR S7 OR S8 OR S9 OR S10 OR S11 OR S12 OR S13 OR S14 OR S15 OR S16 OR S17 OR S18 OR S19 OR S20 OR S21 OR S22 OR S23 OR S24 | 157,687 |
| S41 | S37 AND S40 | 486 |
| S42 | S38 OR S39 OR S41 | 511 |

**Database:** Maternity & Infant Care Database (MIDIRS) 1971 to January 2017

**Date:**  15.03.2017

**Results:** 385

**Results after update of searches 05.01.2019:** 434

| # | Searches | Results |
| --- | --- | --- |
| 1 | Intermittent Auscultation Versus Continuous Fetal Monitoring.ss. | 141 |
| 2 | Labour.de. | 15852 |
| 3 | Labour stage.de. | 4 |
| 4 | Labour stage - first.de. | 263 |
| 5 | Labour stage - second.de. | 541 |
| 6 | Labour duration.de. | 443 |
| 7 | Labour complications.de. | 1608 |
| 8 | Delivery.de. | 1340 |
| 9 | Childbirth.de. | 2242 |
| 10 | Pregnancy.de. | 57538 |
| 11 | Pregnancy - high-risk.de. | 734 |
| 12 | Gravidity.de. | 23 |
| 13 | Prenatal care.de. | 3 |
| 14 | perinatal care.de. | 809 |
| 15 | prenatal diagnosis.de. | 4497 |
| 16 | Fetal heart.de. | 454 |
| 17 | Heart rate - fetal.de. | 910 |
| 18 | Fetal monitoring.de. | 1498 |
| 19 | Fetus.de. | 3057 |
| 20 | (fetal or foetal or fetus* or foetus*).mp. | 42019 |
| 21 | (labor or laboring or labour or labouring).mp. | 31417 |
| 22 | pregnan*.mp. | 98745 |
| 23 | (childbirth* or child birth*).mp. | 13031 |
| 24 | parturition*.mp. | 512 |
| 25 | (intrapartum or antenatal* or prenatal* or perinatal*).mp. | 54359 |
| 26 | (obstetric* adj2 deliver*).mp. | 341 |
| 27 | or/1-26 | 137354 |
| 28 | Auscultation.de. | 62 |
| 29 | Auscultation - intermittent.de. | 47 |
| 30 | Heart auscultation.de. | 14 |
| 31 | Stethoscopes.de. | 4 |
| 32 | Pinard stethoscopes.de. | 20 |
| 33 | Fetoscope.de. | 4 |
| 34 | Hand-held Doppler.de. | 1 |
| 35 | auscultat*.mp. | 273 |
| 36 | (pinard* or pinnard*).mp. | 40 |
| 37 | la?nnec*.mp. | 4 |
| 38 | isia.mp. | 2 |
| 39 | delee hillis.mp. | 0 |
| 40 | (sonicaid* or sonic aid*).mp. | 45 |
| 41 | fetoscope*.mp. | 20 |
| 42 | stethoscope*.mp. | 57 |
| 43 | ((handheld or hand held) adj3 (doppler or ultrasound* or transducer*)).mp. | 18 |
| 44 | (intermittent adj2 monitor*).mp. | 39 |
| 45 | or/28-44 | 397 |
| 46 | 27 and 45 | 351 |
| 47 | ((f?etal monitoring or cardiotocograph* or ctg or f?etal heart rate* or fhr) adj3 intermittent).mp. | 51 |
| 48 | ((obstetric or f?etal) adj5 stethoscope*).mp. | 9 |
| 49 | 1 or 46 or 47 or 48 | 385 |

Comment: mp= abstract, heading word, title

**Database:** Cochrane Library

**Date:**  15.03.2017

**Results:** 93 (Cochrane reviews (12), Other Reviews (4), Trials (72), Methods Studies (2), Technology Assessments (1), Economic Evaluations (1) Cochrane Groups (1)

**Results after update of searches 05.01.2019:** 403 (Cochrane reviews (25), Trials (374), Protocols (2), Clinical answers (2))

| #1 | MeSH descriptor: [Labor Stage, First] this term only | 195 |
| --- | --- | --- |
| #2 | MeSH descriptor: [Labor Stage, Second] this term only | 163 |
| #3 | MeSH descriptor: [Labor, Obstetric] this term only | 958 |
| #4 | MeSH descriptor: [Labor Onset] this term only | 63 |
| #5 | MeSH descriptor: [Natural Childbirth] this term only | 41 |
| #6 | MeSH descriptor: [Obstetric Labor Complications] this term only | 466 |
| #7 | MeSH descriptor: [Delivery, Obstetric] this term only | 1087 |
| #8 | MeSH descriptor: [Parturition] this term only | 124 |
| #9 | MeSH descriptor: [Pregnancy] this term only | 61 |
| #10 | MeSH descriptor: [Pregnancy, High-Risk] this term only | 203 |
| #11 | MeSH descriptor: [Gravidity] this term only | 57 |
| #12 | MeSH descriptor: [Prenatal Care] this term only | 1281 |
| #13 | MeSH descriptor: [Prenatal Diagnosis] this term only | 376 |
| #14 | MeSH descriptor: [Perinatal Care] this term only | 159 |
| #15 | MeSH descriptor: [Fetus] this term only | 364 |
| #16 | MeSH descriptor: [Heart Rate, Fetal] this term only | 330 |
| #17 | MeSH descriptor: [Fetal Heart] this term only | 107 |
| #18 | MeSH descriptor: [Fetal Monitoring] this term only | 267 |
| #19 | (fetal or foetal or fetus* or foetus*):ti,ab,kw | 8712 |
| #20 | (labor or laboring or labour or labouring):ti,ab,kw | 10062 |
| #21 | (pregnan* or parturition* or childbirth* or child birth*):ti,ab,kw | 36236 |
| #22 | (intrapartum or prenatal* or perinatal* or antenatal*):ti,ab,kw | 9056 |
| #23 | (obstetric* near/2 deliver*):ti,ab,kw | 1286 |
| #24 | {or #1-#23} | 42971 |
| #25 | MeSH descriptor: [Auscultation] this term only | 111 |
| #26 | MeSH descriptor: [Heart Auscultation] this term only | 53 |
| #27 | MeSH descriptor: [Stethoscopes] this term only | 25 |
| #28 | MeSH descriptor: [Fetoscopes] this term only | 1 |
| #29 | auscultat*:ti,ab,kw | 471 |
| #30 | ((handheld or hand held) near/3 (doppler or ultrasound* or transducer*)):ti,ab,kw 38 | |
| #31 | (pinard* or pinnard*):ti,ab,kw | 9 |
| #32 | la*nnec*:ti,ab,kw | 8 |
| #33 | isia:ti,ab,kw | 0 |
| #34 | delee hillis:ti,ab,kw | 0 |
| #35 | (sonicaid* or sonic aid*):ti,ab,kw | 9 |
| #36 | fetoscope*:ti,ab,kw | 7 |
| #37 | (intermittent near/2 monitor*):ti,ab,kw | 63 |
| #38 | {or #25-#37} | 578 |
| #39 | #24 and #38 | 79 |
| #40 | ((f*etal monitoring or cardiotocograph* or ctg or f*etal heart or fhr) near/5 intermittent) | 44 |
| #41 | ((obstetric or f*etal) near/5 stethoscope*):ti,ab,kw | 5 |
| #42 | #39 or #40 or #41 | 93 |

**Database:** SveMed+

**Date:**  15.03.2017

**Results:** 86

**Results after update of searches 05.01.2019:** Database error – not possible to reproduce the search

| 1 | [noexp:"labor obstetric"](http://svemedplus.kib.ki.se/Default.aspx?query=noexp:%22labor%20obstetric%22) | 304 |
| --- | --- | --- |
| 2 | [noexp:"labor onset"](http://svemedplus.kib.ki.se/Default.aspx?query=noexp:%22labor%20onset%22) | 15 |
| 3 | [noexp:"labor stage first"](http://svemedplus.kib.ki.se/Default.aspx?query=noexp:%22labor%20stage%20first%22) | 2 |
| 4 | [noexp:"labor stage second"](http://svemedplus.kib.ki.se/Default.aspx?query=noexp:%22labor%20stage%20second%22) | 10 |
| 5 | [noexp:"delivery obstetric"](http://svemedplus.kib.ki.se/Default.aspx?query=noexp:%22delivery%20obstetric%22) | 333 |
| 6 | [noexp:"natural childbirth"](http://svemedplus.kib.ki.se/Default.aspx?query=noexp:%22natural%20childbirth%22) | 28 |
| 7 | [noexp:"Parturition"](http://svemedplus.kib.ki.se/Default.aspx?query=noexp:%22Parturition%22) | 72 |
| 8 | [noexp:"Obstetric Labor Complications"](http://svemedplus.kib.ki.se/Default.aspx?query=noexp:%22Obstetric%20Labor%20Complications%22) | 213 |
| 9 | [noexp:"Pregnancy"](http://svemedplus.kib.ki.se/Default.aspx?query=noexp:%22Pregnancy%22) | 4246 |
| 10 | [noexp:"pregnancy high-risk"](http://svemedplus.kib.ki.se/Default.aspx?query=noexp:%22pregnancy%20high-risk%22) | 12 |
| 11 | [noexp:"gravidity"](http://svemedplus.kib.ki.se/Default.aspx?query=noexp:%22gravidity%22) | 11 |
| 12 | [noexp:"prenatal care"](http://svemedplus.kib.ki.se/Default.aspx?query=noexp:%22prenatal%20care%22) | 62 |
| 13 | [noexp:"Prenatal Diagnosis"](http://svemedplus.kib.ki.se/Default.aspx?query=noexp:%22Prenatal%20Diagnosis%22) | 267 |
| 14 | [noexp:"perinatal care"](http://svemedplus.kib.ki.se/Default.aspx?query=noexp:%22perinatal%20care%22) | 32 |
| 15 | [noexp:"fetus"](http://svemedplus.kib.ki.se/Default.aspx?query=noexp:%22fetus%22) | 77 |
| 16 | [noexp:"fetal heart"](http://svemedplus.kib.ki.se/Default.aspx?query=noexp:%22fetal%20heart%22) | 10 |
| 17 | [noexp:"heart rate fetal"](http://svemedplus.kib.ki.se/Default.aspx?query=noexp:%22heart%20rate%20fetal%22) | 20 |
| 18 | [noexp:"fetal monitoring"](http://svemedplus.kib.ki.se/Default.aspx?query=noexp:%22fetal%20monitoring%22) | 55 |
| 19 | [gravid* OR svangerskap* OR lavrisikoföd* OR födsel* OR föde* OR födande OR foster*](http://svemedplus.kib.ki.se/Default.aspx?query=gravid*%20OR%20svangerskap*%20OR%20lavrisikof%C3%B6d*%20OR%20f%C3%B6dsel*%20OR%20f%C3%B6de*%20OR%20f%C3%B6dande%20OR%20foster*) | 5039 |
| 20 | [fetal OR foetal OR fetus* OR foetus* OR labor OR laboring OR labour OR labouring OR pregnan* OR childbirth* OR child birth* OR parturition* OR intrapartum OR antenatal* OR prenatal* OR perinatal*](http://svemedplus.kib.ki.se/Default.aspx?query=fetal%20OR%20foetal%20OR%20fetus*%20OR%20foetus*%20OR%20labor%20OR%20laboring%20OR%20labour%20OR%20labouring%20OR%20pregnan*%20OR%20childbirth*%20OR%20child%20birth*%20OR%20parturition*%20OR%20intrapartum%20OR%20antenatal*%20OR%20prenatal*%20OR%20perinatal*) | 4899 |
| 21 | [#1 OR #2 OR #3 OR #4 OR #5 OR #6 OR #7 OR #8 OR #9 OR #10 OR #11 OR #12 OR #13 OR #14 OR #15 OR #16 OR #17 OR #18 OR #19 OR #20](http://svemedplus.kib.ki.se/Default.aspx?query=%231%20OR%20%232%20OR%20%233%20OR%20%234%20OR%20%235%20OR%20%236%20OR%20%237%20OR%20%238%20OR%20%239%20OR%20%2310%20OR%20%2311%20OR%20%2312%20OR%20%2313%20OR%20%2314%20OR%20%2315%20OR%20%2316%20OR%20%2317%20OR%20%2318%20OR%20%2319%20OR%20%2320) | 5317 |
| 22 | [noexp:"auscultation"](http://svemedplus.kib.ki.se/Default.aspx?query=noexp:%22auscultation%22) | 21 |
| 23 | [noexp:"heart auscultation"](http://svemedplus.kib.ki.se/Default.aspx?query=noexp:%22heart%20auscultation%22) | 15 |
| 24 | [noexp:"Stethoscopes"](http://svemedplus.kib.ki.se/Default.aspx?query=noexp:%22Stethoscopes%22) | 15 |
| 25 | [noexp:"fetoscopes"](http://svemedplus.kib.ki.se/Default.aspx?query=noexp:%22fetoscopes%22) | 0 |
| 26 | [auscultat* OR pinard* OR pinnard* OR doppler OR fetoscope* OR isia OR stethoscope* OR sonicaid* OR "sonic aid"](http://svemedplus.kib.ki.se/Default.aspx?query=auscultat*%20OR%20pinard*%20OR%20pinnard*%20OR%20doppler%20OR%20fetoscope*%20OR%20isia%20OR%20stethoscope*%20OR%20sonicaid*%20OR%20%22sonic%20aid%22) | 231 |
| 27 | [laennec* OR "delee hillis"](http://svemedplus.kib.ki.se/Default.aspx?query=laennec*%20OR%20%22delee%20hillis%22) | 4 |
| 28 | [Hjärtauskultation OR auskult* OR stetoskop*](http://svemedplus.kib.ki.se/Default.aspx?query=Hj%C3%A4rtauskultation%20OR%20auskult*%20OR%20stetoskop*) | 44 |
| 29 | [handheld OR "hand held" OR håndholdt OR handhållna](http://svemedplus.kib.ki.se/Default.aspx?query=handheld%20OR%20%22hand%20held%22%20OR%20h%C3%A5ndholdt%20OR%20handh%C3%A5llna) | 32 |
| 30 | [#22 OR #23 OR #24 OR #25 OR #26 OR #27 OR #28 OR #29](http://svemedplus.kib.ki.se/Default.aspx?query=%2322%20OR%20%2323%20OR%20%2324%20OR%20%2325%20OR%20%2326%20OR%20%2327%20OR%20%2328%20OR%20%2329) | 265 |
| 31 | [#21 AND #30](http://svemedplus.kib.ki.se/Default.aspx?query=%2321%20AND%20%2330) | 14 |
| 32 | [fetal monitoring OR fosterovervåkning OR Fosterövervakning](http://svemedplus.kib.ki.se/Default.aspx?query=fetal%20monitoring%20OR%20fosteroverv%C3%A5kning%20OR%20Foster%C3%B6vervakning) | 76 |
| 33 | [#31 OR #32](http://svemedplus.kib.ki.se/Default.aspx?query=%2331%20OR%20%2332) | 86 |

**Database:** Web of Science, Indexes=SCI-EXPANDED, ESCI Timespan=All years

**Date:**  20.03.2017

**Results:** 356

**Results after update of searches 05.01.2019:** 418

| #1 | TS=(labor OR laboring OR labour OR labouring OR pregnan* OR (deliver* NEAR/2 obstetric*) OR childbirth* OR "child birth*" OR parturition* OR intrapartum OR antenatal* OR prenatal* OR perinatal* OR fetal OR foetal OR fetus* OR foetus*) | 595,110 |
| --- | --- | --- |
| #2 | TS=(auscultat* OR fetoscope* OR pinard* OR pinnard* OR la$nnec OR isia OR "delee hillis" OR sonicaid* OR "sonic aid*" OR stethoscope*) | 4,238 |
| #3 | TS=(intermittent near/2 monitor*) | 292 |
| #4 | TS=((handheld OR "hand held") NEAR/2 (doppler OR ultrasound* OR transducer*)) | 505 |
| #5 | #4 OR #3 OR #2 | 4,993 |
| #6 | #5 AND #1 | 344 |
| #7 | TS=(("f$etal monitoring" or cardiotocograph* or ctg or "f$etal heart rate*" or fhr) NEAR/3 intermittent) | 55 |
| #8 | TS=((obstetric or f$etal) NEAR/5 stethoscope*) | 3 |
| #9 | #8 OR #7 OR #6 | 356 |

**Database:** Scopus

**Date:**  20.03.2017

**Results:** 1554

**Results after update of searches 05.01.2019:** 1695

| ( ( ( TITLE-ABS-KEY ( auscultat*  OR  fetoscope*  OR  pinard*  OR  pinnard*  OR  laennec*  OR  isia  OR  "delee hillis"  OR  sonicaid*  OR  "sonic aid"  OR  "sonic aids"  OR  stethoscope*  OR  ( intermittent  W/2  monitor* )  OR  ( ( handheld  OR  "hand held" )  W/2  ( doppler  OR  ultrasound*  OR  transducer* ) ) ) )  AND  ( TITLE-ABS-KEY ( labor  OR  laboring  OR  labour  OR  labouring  OR  pregnan*  OR  ( deliver*  W/2  obstetric* )  OR  childbirth*  OR  "child birth"  OR  "child births"  OR  parturition*  OR  intrapartum  OR  antenatel*  OR  prenatal*  OR  perinatal*  OR  fetal  OR  foetal  OR  fetus*  OR  foetus* ) ) )  OR  ( TITLE-ABS-KEY ( ( "fetal monitoring"  OR  "foetal monitoring"  OR  cardiotocograph*  OR  ctg  OR  "fetal heart rate*"  OR  "foetal heart rate*"  OR  fhr )  W/2  intermittent ) ) )  OR  ( ( ( obstetric  OR  fetal  OR  foetal )  W/5  stethoscope* ) ) |
| --- |
| Child birth* = many irrelevant references (birth weight) |

**Database:** LILACS

http://bases.bireme.br/cgibin/wxislind.exe/iah/online/?IsisScript=iah/iah.xis&base=LILACS&lang=i&form=A

**Date:**  20.03.2017

**Results:** 42

**Results after update of searches 05.01.2019:** 49

| auscultat$ OR fetoscope$ OR pinard$ OR pinnard$ OR laennec$ OR isia OR delee hillis OR sonicaid$ OR sonic aid$ OR stethoscope$ OR handheld Doppler OR hand held Doppler OR handheld ultrasound$ OR hand held ultrasound$ OR handheld transducer$ OR hand held transducer$ [Words] and labor OR laboring OR labour OR labouring OR pregnan$ OR deliver$ OR childbirth$ OR birth$ OR parturition$ OR intrapartum OR antenatal$ OR prenatal$ OR perinatal$ OR fetal OR foetal OR fetus$ OR foetus$ [Words] |
| --- |

| intermittent monitor$ [Words]  monitor$ intermittent [Words] | 0=hits |
| --- | --- |
| fetal monitor$ foetal monitor$ OR cardiotocograph$ OR ctg OR fetal heart rate$ OR foetal heart rate$ OR fhr [Words] and intermittent [Words] | 2=hits (one unique) |
| obstetric stethoscope* [Words] | 0=hits |

**Database:** African Journals Online (AJOL): *Advanced search (all titles) https://www.ajol.info/index.php/index/search*

**Date:**  04.04.2017

**Results:** 54

**Results after update of searches 05.01.2019:** 64

| auscultat* OR fetoscope* OR pinard* OR pinnard* OR Laennec* OR isia OR "delee hillis" OR sonicaid* OR "sonic aid" OR "sonic aids" OR stethoscope* OR "handheld doppler" OR "hand held Doppler" | 36 |
| --- | --- |
| "intermittent monitoring" | 1 (irrelevant) |
| (cardiotocograph* OR ctg OR fetal heart rate OR fetal heart rates OR fhr) AND intermittent) | 3 (2 = irrelevant 1= identified (main search)) |
| "fetal monitoring" OR "foetal monitoring" | 14 (see trough) |

Comment: Could not export to EndNote.
