# Supplementary material for: Intermittent auscultation fetal monitoring during labour: A systematic scoping review to identify methods, effects, and accuracy
Source: PLoS One. 2019 Jul 10;14(7):e0219573. doi: 10.1371/journal.pone.0219573 (PMC6619817; doi:10.1371/journal.pone.0219573)
Supplement: S2 Table — A list of the 238 articles that were excluded after full-text assessment and the reason for exclusion. (DOCX) [file pone.0219573.s002.docx]

**S2 Table. Articles excluded after full text assessment**

|  | Adams WO. Observations on mediate auscultation, as a practical guide in difficult labours. The Dublin Journal of Medical and Chemical Science. 1833;3(1):65-73. | Not relevant information |
| --- | --- | --- |
|  | Ahmad HAB, El-Badawy IM, Singh OP, Hisham RB, Malarvili MB. Fetal heart rate monitoring device using condenser microphone sensor: Validation and comparison to standard devices. Technology and Health Care. 2018;26(4):573-9. | Not relevant, for antenatal care |
|  | Ainsworth S. The view within. The fascinating history behind the stethoscope and the electronic fetal monitor. Practising Midwife. 2002;5(9):26. | Commentary |
|  | Akileswaran CP, Hutchison MS. Making Room at the Table for Obstetrics, Midwifery, and a Culture of Normalcy Within Maternity Care. Obstet Gynecol. 2016;128(1):176-80. | Not relevant information |
|  | Albers LL. Monitoring the fetus in labor: evidence to support the methods. J Midwifery Womens Health. 2001;46(6):366-73. | Review |
|  | Albers LL. The evidence for physiologic management of the active phase of the first stage of labor. J Midwifery Womens Health. 2007;52(3):207-15. | Review |
|  | Alfirevic Z, Devane D, Gyte GM, Cuthbert A. Continuous cardiotocography (CTG) as a form of electronic fetal monitoring (EFM) for fetal assessment during labour. Cochrane Database Syst Rev. 2017;2:CD006066. | Review |
|  | Ali E. Pinnard obsolete?...Blake D (2008) Pinards: out of use and out of date? British Journal of Midwifery 16(6): 364-5. British Journal of Midwifery. 2008;16(7):480-. | Letter to the editor |
|  | Altaf S, Oppenheimer C, Shaw R, Waugh J, Dixon-Woods M. Practices and views on fetal heart monitoring: a structured observation and interview study. Bjog-an International Journal of Obstetrics and Gynaecology. 2006;113(4):409-18. | Not relevant information |
|  | American College of Nurse-Midwives. Intermittent Auscultation for Intrapartum Fetal Heart Rate Surveillance. Journal of Midwifery & Womens Health, vol 55, pg 397, 2010. | Expired guidelines |
|  | Anderson I. Portable fetal monitoring: a new option for intermittent fetal monitoring. MIDIRS Midwifery Digest. 1994;4:439-40. | Not relevant information |
|  | Anonymous. Erratum to Intermittent Auscultation for Intrapartum Fetal Heart Rate Surveillance, [Journal of Midwifery & Women's Health (2015), 60[5]:626-632]. Journal of Midwifery and Women's Health. 2016;61(1):134-. | Erratum |
|  | Association of Women’s Health Obstetric and Neonatal Nurses. Fetal Heart Monitoring. Nursing for Women's Health. 2015;19(6):557-60. | Expired guideline |
|  | Austin JM, Jr., New TG, Flowers CE, Jr. Fetal heart sound monitoring in high risk patients. Ala J Med Sci. 1972;9(3):305-9. | Not relevant information |
|  | Ayres-de-Campos D, Arulkumaran S, Panel FIFMEC. FIGO consensus guidelines on intrapartum fetal monitoring: Physiology of fetal oxygenation and the main goals of intrapartum fetal monitoring. Int J Gynaecol Obstet. 2015;131(1):5-8. | Not relevant information |
|  | Ayres-De-Campos D, Arulkumaran S. FIGO consensus guidelines on intrapartum fetal monitoring: Introduction. International Journal of Gynecology and Obstetrics. 2015;131(1):3-4. | Not relevant information |
|  | Banta D, Thacker S. Electronic fetal monitoring: is it of benefit? Birth Fam J. 1979;6(4):237-49. | Review |
|  | Bartholomew RA. The prevention of fetal asphyxia. Am J Obstet Gynecol. 1923;6(4):418-26. | Historical review |
|  | Benson RC, Shubeck F, Deutschberger J, Weiss W, Berendes H. Fetal heart rate as a predictor of fetal distress. A report from the collaborative project. Obstet Gynecol. 1968;32(2):259-66. | Not relevant information |
|  | Birch L, Thompson B. Survey into fetal monitoring practices and attitudes. British Journal of Midwifery. 1997;5(12):732-6. | Not relevant information |
|  | Blake D. Pinards: out of use and out of date? British Journal of Midwifery. 2008;16(6):364-5. | Review |
|  | Blincoe AJ. Fetal monitoring challenges and choices for midwives. British Journal of Midwifery. 2005;13(2):108-11. | Review |
|  | Blix E, Reinar LM, Klovning A, Øian P. Prognostic value of the labour admission test and its effectiveness compared with auscultation only: a systematic review . BJOG An International Journal of Obstetrics and Gynaecology . 2005; 112(12):[1595-604 pp.]. | Review |
|  | Borg E. Intermittent auscultation of the fetal heart rate. Can Nurse. 2003;99(8):40-1. | Not relevant, case report |
|  | Boylan P. Intrapartum fetal monitoring. Baillieres Clin Obstet Gynaecol. 1987;1(1):73-95. | Not scientific article |
|  | British Columbia Reproductive Care P. Obstetric guideline 6A: Intermittent auscultation in labour. Vancouver: British Columbia Reproductive Care Program; 2005. p. 7. | Regional guideline |
|  | Brown AD, Robertson JG. The ultrasonic Doppler cardioscope in obstetrics. J Obstet Gynaecol Br Commonw. 1968;75(1):92-6. | Not relevant information |
|  | Cahill AG, Spain J. Intrapartum fetal monitoring. Clin Obstet Gynecol. 2015;58(2):263-8. | Not relevant information |
|  | Caplan RM. Electronic fetal-heart-rate monitoring as compared with periodic auscultation and the neurologic development of premature infants. N Engl J Med. 1990;323(5):345-8. | Letter to the editor |
|  | Chez BF, Harvey MG, Harvey CJ. Intrapartum fetal monitoring: past, present, and future. J Perinat Neonatal Nurs. 2000;14(3):1-18. | Review |
|  | Chinula L, Fawcus S, Woods D. Comparing an innovative doppler ultrasound fetal heart rate monitor to a pinard fetal stethoscope using cardiotocography as a standard in women with singleton pregnancies in labour at mowbray maternity hospital, South Africa. International Journal of Gynecology and Obstetrics. 2015;131:E121 | Abstract |
|  | Cibils LA. On intrapartum fetal monitoring. Am J Obstet Gynecol. 1996;174(4):1382-9. | Review |
|  | Colditz PB, Henderson-Smart DJ. Electronic fetal heart rate monitoring during labour: does it prevent perinatal asphyxia and cerebral palsy? Med J Aust. 1990;153(2):88-90. | Not relevant information |
|  | Cole C. Admission electronic fetal monitoring does not improve neonatal outcomes. J Fam Pract. 2003;52(6):443-4. | Not relevant information |
|  | Collins D, Branch RF. Fetal Heart Tone Monitor. Obstet Gynecol. 1964;23:457-9. | Not relevant information |
|  | Cox KJ, King TL. Preventing primary cesarean births: midwifery care. Clin Obstet Gynecol. 2015;58(2):282-93. | Not relevant information |
|  | Crane AD. Intermittent auscultation for intrapartum fetal heart rate surveillance clinical bulletin no. 11. J Midwifery Womens Health. 2010;55:397-403. J Midwifery Womens Health. 2011;56(4):409-. | Letter to the editor |
|  | Cranston CS. Obstetrical nurses' attitude toward fetal monitoring. JOGN Nurs. 1980;9(6):344-7. | Not relevant information |
|  | Cronk M. Me and my Pinard's. Midwifery Matters. 2002(94):3-4. | Review |
|  | Crozier K, Sinclair M. Medical device training in maternity care: part 1. British Journal of Midwifery. 2004;12(7):422-6. | Not relevant information |
|  | D’Souza R, Arulkumaran S. Intrapartum fetal surveillance. Best Practice in Labour and Delivery2009. p. 38-53. | Not scientific article |
|  | Dalmacion GV, Reyles RT, Habana AE, et al. Handheld ultrasound to avert maternal and neonatal deaths in 2 regions of the Philippines: an iBuntisReg. intervention study. BMC Pregnancy and Childbirth. 2018 | Not relevant information |
|  | Dawson JA, Schmolzer GM, Wyllie J. Monitoring heart rate in the delivery room. Semin Fetal Neonatal Med. 2018;23(5):327-32. | Not relevant information |
|  | Devane D, Lalor JG, Daly S, McGuire W, Cuthbert A, Smith V. Cardiotocography versus intermittent auscultation of fetal heart on admission to labour ward for assessment of fetal wellbeing. Cochrane Database Syst Rev. 2017;1:CD005122. | Review |
|  | Devane D, Smith V, Healy P. Cardiotocography for assessment of fetal wellbeing during labour. Practising Midwife. 2010;13(6):18-20. | Commentary |
|  | Dildy GA, 3rd. Intrapartum assessment of the fetus: historical and evidence-based practice. Obstet Gynecol Clin North Am. 2005;32(2):255-71, ix. | Review |
|  | Dover SL, Gauge SM. Fetal monitoring - midwifery attitudes. Midwifery. 1995;11(1):18-27. | Not relevant information |
|  | Dresang LT, Yonke N. Management of Spontaneous Vaginal Delivery. Am Fam Physician. 2015;92(3):202-8. | Not relevant information |
|  | Dunn PM. Dr. Robert Collins (1801-1868) and his Rotunda obstetric report. Archives of Disease in Childhood Fetal & Neonatal Edition. 1994;71(1):F67-8. | Not relevant information |
|  | Dutch Association of Obstetrics and Gynaecology. NVOG-richtlijn Intrapartum foetale bewaking à terme. Utrecht, NVOG, 2014 | Not relevant, on EFM only |
|  | Ellison PH, Foster M, Sheridan-Pereira M, MacDonald D. Electronic fetal heart monitoring, auscultation, and neonatal outcome. Am J Obstet Gynecol. 1991;164(5 Pt 1):1281-9. | Not relevant |
|  | Everden C, Kirkpatrick A, Modarres M. Fetal monitoring in labour in obese mothers. BJOG. 2015;122:290-1. | Not relevant information |
|  | Fainberg VB. [Methods of auscultation of the fetal heart]. Feldsher Akush. 1954;9:10-4. | Article in Russian |
|  | Falls FH, Hunter TA. An improved head stethoscope for the hearing and counting of fetal heart tones. Am J Obstet Gynecol. 1924;8(3):356-8. | Historical review |
|  | Feinstein NF, Sprague A, Trepanier MJ. Fetal heart rate auscultation. Comparing auscultation to electronic fetal monitoring. AWHONN Lifelines. 2000;4(3):35-44. | Review |
|  | Feinstein NF. Fetal heart rate auscultation: current and future practice. JOGNN - Journal of Obstetric, Gynecologic, & Neonatal Nursing. 2000;29(3):306-15. | Review |
|  | Fetal monitoring in VBAC. Birthing Beautifully. 2001:36-9. | Unable to retrieve full-text |
|  | Fetal stethoscope now in bamboo. Safe Motherhood. 1993(13):8. | Commentary |
|  | FIGO Study Group on the Assessment of New Technology. Intrapartum surveillance: recommendations on current practice and overview of new developments. FIGO Study Group on the Assessment of New Technology. International Federation of Gynecology and Obstetrics. Int J Gynaecol Obstet. 1995;49(2):213-21. | Not relevant information as the recommendations do not reflect an official position of FIGO |
|  | Flamm B, Mac Donald D, Shearer E, Mahan CS. Roundtable discussion: should the electronic fetal monitor always be used for women in labor who are having a vaginal birth after a previous cesarean section? Birth: Issues in Perinatal Care. 1992;19(1):31-5. | Commentary |
|  | Fraser J. The cost of making a mistake. Practising Midwife. 2010;13(7):4-5. | Commentary |
|  | Freire G. Surveillance of fetal arrhythmias in the outpatient setting: current limitations and call for action. Cardiol Young. 2015;25(8):1590-2. | Not relevant information |
|  | Garcia J, Corry M, MacDonald D, Elbourne D, Grant A. Mothers' views of continuous electronic fetal heart monitoring and intermittent auscultation in a randomized controlled trial. Birth. 1985;12(2):79-86. | Not relevant information |
|  | Gardosi J. Fetal monitoring during labour. Curr Anaesth Crit Care. 1991;2(3):187-93. | Review |
|  | Girard JA. Auscultation as an adjunct in obstetrics. Journal of the American Medical Womens Association. 1954;9(8):248-9; passim. | Not relevant information |
|  | Goodwin L. Intermittent auscultation of the fetal heart rate: a review of general principles. J Perinat Neonatal Nurs. 2000;14(3):53-61. | Review |
|  | Gourounti K, Sandall J. Admission cardiotocography versus intermittent auscultation of fetal heart rate: effects on neonatal Apgar score, on the rate of caesarean sections and on the rate of instrumental delivery--a systematic review. Int J Nurs Stud. 2007;44(6):1029-35. | Review |
|  | Govender L. Adjunctive techniques of fetal assessment in labour. International Journal of Gynecology and Obstetrics. 2009;107:S33. | Not relevant information |
|  | Graham ID, Logan J, Davies B, et al. Changing the use of electronic fetal monitoring and labor support: a case study of barriers and facilitators. Birth. 2004;31(4):293-301. | Not relevant information |
|  | Greer J. Are midwives irrational or afraid? Evidence Based Midwifery. 2010;8(2):47-52. | Not relevant information |
|  | Gribbin C, James D. Assessing fetal health. Current Obstetrics and Gynaecology. 2005;15(4):221-7. | Not relevant information |
|  | Groedel FM, Miller M, Neugarten L. Direct auscultation and recording of fetal heart sounds. Pflugers Arch Gesamte Physiol Menschen Tiere. 1952;255(2):143-5. | Not relevant information |
|  | Groom D, Sihvonen YT, Bass A, Francis WW. Augmentation of the Audibility of Fetal Heart Sounds by Frequency Multiplication. Am J Obstet Gynecol. 1964;90:345-9. | Not relevant information |
|  | Grunewald C, Håkansson S, Högberg U, Millde Luthander C, Sandin-Bojö A-K, Wiklund I. Svensk förlossningsvård säkras i ett rikstäckande projekt. Tvärprofessionell samverkan en grundpelare i »Säker förlossningsvård. Lakartidningen. 2012;109(19):956-9. | Not relevant information |
|  | Haggerty LA. Continuous electronic fetal monitoring: contradictions between practice and research. JOGNN - Journal of Obstetric, Gynecologic, & Neonatal Nursing. 1999;28(4):409-16. | Review |
|  | Hale R. Monitoring fetal and maternal wellbeing. British Journal of Midwifery. 2008;16(10):682-5. | Not relevant information |
|  | Hale R. Non-invasive techniques for fetal monitoring in pregnancy and labour. British Journal of Midwifery. 2009;17(10):661-5. | Not relevant information |
|  | Hansen PK, Smith SF, Nim J, Neldam S, Osler M. Maternal attitudes to fetal monitoring. Eur J Obstet Gynecol Reprod Biol. 1985;20(1):43-51. | Not relevant information |
|  | Harding C. How to... 'listen' for fetal wellbeing. RCM Midwives. 2012;15(4):32-3. | Commentary |
|  | Harrison J. Auscultation: the art of listening. RCM Midwives. 2004;7(2):64-9. | Review |
|  | Harvey B. Use of CTG monitoring: are recommendations suitable? RCM Midwives. 2004;7(12):518-20. | Commentary |
|  | Hawisa KT, Gabassa N. Comparative study between intermittent auscultation and cardiotocogram in low risk group during labour in Libya. Libyan Journal of .medical Research. 2014;8(1):8-13. | Not relevant information |
|  | Heelan L. Exploring the relationships of power, atiitudes regarding intermittent fetal monitoring, and perceived barriers to research utilization with a labor and delivery nurse's attitude toward patient advocacy: Seton Hall University; 2015. | Not relevant information |
|  | Heelan L. Fetal monitoring: creating a culture of safety with informed choice. J Perinat Educ. 2013;22(3):156-65. | Not relevant information |
|  | Herbst A. Att förebygga förlossningsasfyxi: Kan vi komma längre med fosterövervakning? [Prevention of perinatal asphyxia. Can more be done by fetal monitoring?]. Lakartidningen. 2000;97(32-33):3484-8. | Review |
|  | Hersh S, Megregian M, Emeis C. Intermittent auscultation of the fetal heart rate during labor: an opportunity for shared decision making. J Midwifery Womens Health. 2014;59(3):344-9. | Review |
|  | Hill JB, Chauhan SP, Magann EF, Morrison JC, Abuhamad AZ. Intrapartum fetal surveillance: review of three national guidelines. Am J Perinatol. 2012;29(7):539-50. | Review |
|  | Hill K. An exploration of the views and experiences of midwives using intermittent auscultation of the fetal heart in labor. International Journal of Childbirth. 2016;6(2):68-77. | Not relevant information |
|  | Hindley C, Hinsliff SW, Thomson AM. English Midwives' Views and Experiences of Intrapartum Fetal Heart Rate Monitoring in Women at Low Obstetric Risk: Conflicts and Compromises. Journal of Midwifery and Women's Health. 2006;51(5):354-60. | Not relevant information |
|  | Hindley C, Hinsliff SW, Thomson AM. Pregnant womens' views about choice of intrapartum monitoring of the fetal heart rate: a questionnaire survey. Int J Nurs Stud. 2008;45(2):224-31. | Not relevant information |
|  | Hindley C, Thomson AM. The rhetoric of informed choice: Perspectives from midwives on intrapartum fetal heart rate monitoring. Health Expect. 2005;8(4):306-14. | Not relevant information |
|  | Hon EH. Fetal monitoring for the practicing physician. Calif Med. 1970;113(6):46-7. | Letter to the editor |
|  | Hon EH. The Foetal Heart Rate. Mod Trends Hum Reprod Physiol. 1963;15:245-56. | Not relevant information |
|  | Hunt KM. Doptone foetal pulse recorder. Nurs Mirror Midwives J. 1968;126(10):38-9. | Not relevant, antenatal care |
|  | ICEA position statement on the assessment of fetal well-being during labor and birth. International Journal of Childbirth Education. 1997;12(3):42-5. | Not relevant information |
|  | Intermittent Auscultation for Intrapartum Fetal Heart Rate Surveillance...September/October 2015 issue of the Journal of Midwifery & Women’s Health (60[5]:626-632). J Midwifery Womens Health. 2016;61(1):134-. | Erratum |
|  | Internationl Childbirth Education A. ICEA position statement on the assessment of fetal well-being during labor and birth. International Journal of Childbirth Education. 1997;12(3):42-5. | ICEA position statement |
|  | Intrapartum fetal heart rate monitoring: nomenclature, interpretation, and general management principles. ACOG Practice Bulletin No. 106. American College of Obstetricians and Gynecologists. Obstet Gynecol 2009;114:192–202. | Not relevant information |
|  | Jauniaux E, Prefumo F. Fetal heart monitoring in labour: from pinard to artificial intelligence. BJOG. 2016;123(6):870. | Commentary |
|  | Jaynes AC, Scott KE. Intrapartum Care the Midwifery Way: A Review. Primary Care - Clinics in Office Practice. 2012;39(1):190-206. | Review |
|  | John AH. The accuracy of direct auscultation and the normal variation of foetal heart rate. J Obstet Gynaecol Br Commonw. 1966;73(6):983-5. | Not relevant, antenatal care |
|  | Kaiser G. Do electronic fetal heart rate monitors improve delivery outcomes? J Fla Med Assoc. 1991;78(5):303-7. | Review |
|  | Kamala B. Effectiveness of strap-on doppler versus intermittent doppler in abnormal fetal heart rate detection. International Journal of Gynecology and Obstetrics. 2018;143 (Supplement 3):238. | Not relevant information |
|  | Keith P. Fetal heart assessments. Determining which technique to use. Nurs BC. 2002;34(1):29-30. | Review |
|  | Killien MC, Shy K. A randomized trial of electronic fetal monitoring in preterm labour: mothers' views. Birth. 1989;16(1):7-12. | Same study as Luthy DA et al. 1987 which is included. |
|  | Kolarik J, Golembiovsky M, Docekal T, Kahankova R, Martinek R, Prauzek M. A Low-cost Device for Fetal Heart Rate Measurement. IFAC-PapersOnLine. 2018;51(6):426-31. | Not relevant information |
|  | Langendoerfer S, Haverkamp AD, Murphy J, Nowick KD, Orleans M, Pacosa F, et al. Pediatric follow-up of a randomized controlled trial of intrapartum fetal monitoring techiques. J Pediatr. 1980;97(1):103-7. | Not relevant information |
|  | Langli Ersdal H, Mduma E, Svensen E, Sundby J, Perlman J. Intermittent detection of fetal heart rate abnormalities identify infants at greatest risk for fresh stillbirths, birth asphyxia, neonatal resuscitation, and early neonatal deaths in a limited-resource setting. Neonatology . 2012;102(3):235-42. | Not relevant information |
|  | Lawrence L. Fetal heart monitoring with a fetoscope. Midwifery Today Childbirth Educ. 1992(24):30-4. | Not scientific article |
|  | Leslie K, Arulkumaran S. Intrapartum fetal surveillance. Obstetrics, Gynaecology and Reproductive Medicine. 2011;21(3):59-67. | Review |
|  | Lewis D, Downe S. Erratum: FIGO consensus guidelines on intrapartum fetal monitoring: Intermittent auscultation (International Journal of Gynecology and Obstetrics 131 (2015) (9-12)) doi 10.1016/j.ijgo.2015.06.019. International Journal of Gynecology and Obstetrics. 2016;133(1):129. | Erratum |
|  | Lewis L, Rowe J. Focus on the beat: current fetal monitoring practice in low risk labour. Aust J Midwifery. 2004;17(4):6-10. | Not relevant information |
|  | Li YP, Lin SY, Yeh CH, Hsu HC, Yang YL, Lee CN, et al. A proposed mother-friendly childbirth model for Taiwanese women and obstetricians' attitudes toward it. Taiwan J Obstet Gynecol. 2015;54(6):666-70. | Not relevant information |
|  | Li YP, Yeh CH, Lin SY, Chen TC, Yang YL, Lee CN, et al. A proposed mother-friendly childbirth model for Taiwanese women, the implementation and satisfaction survey. Taiwan J Obstet Gynecol. 2015;54(6):731-6. | Not relevant information |
|  | Liston R, Crane J, Hamilton E, Hughes O, Kuling S, MacKinnon C, et al. Fetal health surveillance in labour. Journal of Obstetrics & Gynaecology Canada: JOGC. 2002;24(3):250-76; quiz 77-80. | Expired guideline |
|  | Liston R, Sawchuck D, Young D, Society of O, Gynaecologists of C, British Columbia Perinatal Health P. Fetal health surveillance: antepartum and intrapartum consensus guideline. Journal of Obstetrics & Gynaecology Canada: JOGC. 2007;29(9 Suppl 4):S3-56. | Regional guideline |
|  | Liston R. Eyes and ears, hopes and fears. Journal of Obstetrics & Gynaecology Canada: JOGC. 2002;24(4):307-11. | Editorial |
|  | Lloyd TS, Jr. Obstetrical use of the ultrasonic Doppler instrument. JAMA. 1968;204(13):1195-6. | Not relevant information |
|  | Lutomski JE, Meaney S, Greene RA, Ryan AC, Devane D. Expert systems for fetal assessment in labour. Cochrane Database Syst Rev. 2015(4):N.PAG-N.PAG. | Review |
|  | Liston R, Sawchuck D, Young D. No. 197- Fetal Health Surveillance: Antepartum and Intrapartum Consensus Guideline. J Obstet Gynaecol Can.2007;29(11):S3-56. | Expired guideline |
|  | Luyben AG, Gross MM. Intrapartum fetal heart rate monitoring: do Swiss midwives implement evidence into practice? Eur J Obstet Gynecol Reprod Biol. 2001;96(2):179-82. | Not relevant information |
|  | Lynn MR. Extra! Extra! Nurse beats machine... electronic FHR monitoring may have been 'less effective than a program of periodic auscultation'. J Pediatr Nurs. 1990;5(3):223-5. | Commentary |
|  | M’Clintock AH. A memoir on the use, of auscultation in the treatment of labours. The Dublin Quarterly Journal of Medical Science. 1847;4(1):34-56. | Not relevant information |
|  | MacDonald D. Supervision of the fetus in labour--to listen or to look. Ir Med J. 1985;78(12):343-4. | Editorial |
|  | Mahomed K, Gupta BK, Matikiti L, Murape TS. A simplified form of cardiotocography for antenatal fetal assessment. Midwifery. 1992;8(4):191-4. | Not relevant, antenatal care |
|  | Mangesi L, Hofmeyr GJ, Woods DL. Assessing the preference of women for different methods of monitoring the fetal heart in labour. S Afr J Obstet Gynaecol. 2009;15(2):58-9. | Not relevant information |
|  | Martis R, Emilia O, Nurdiati DS, Brown J. Intermittent auscultation (IA) of fetal heart rate in labour for fetal well-being. Cochrane Database of Systematic Reviews. 2017;2:CD008680 | Review |
|  | Martis R. Intermittent auscultation - 'ripped away from my peaceful place'. Essentially MIDIRS. 2013;4(5):46-9. | Not relevant information |
|  | Maude R, Foureur M, Skinner J. Conscious guardianship of normal birth: The art and science of intelligent structured intermittent auscultation (ISIA) of the fetal heart for low risk women. Women & Birth. 2013;26:S12-S. | Conference abstract |
|  | Maude R, Lawson J, Foureur M. Auscultation -- The Action of Listening. New Zealand College of Midwives Journal. 2010(43):13-8. | Review |
|  | Maude RM, Skinner JP, Foureur MJ. Putting intelligent structured intermittent auscultation (ISIA) into practice. Women & Birth: Journal of the Australian College of Midwives. 2016;29(3):285-92. | Discussion article |
|  | McCrann DJ, Jr., Schifrin BS. Fetal monitoring in high-risk pregnancy. Clin Perinatol. 1974;1(2):229-52. | Not relevant information, about EFM only |
|  | McDonald D. Supervision of the fetus during labour. Ir Med J. 1989;82(3):104. | Commentary |
|  | Mdoe P, Mduma E, Kidanto H, Moshiro R, Perlman J, Ersdal H. Randomized controlled study comparing hand held doppler and pinard fetoscope (PF) for fetal heart rate (FHR) monitoring in Tanzania. International Journal of Gynecology and Obstetrics. 2015;131:E121-E2. | Abstract |
|  | Meddings F. Fetal Doppler: the panacea for fetal heart monitoring. British Journal of Midwifery. 2000;8(3):172-. | Commentary |
|  | Menticoglou SM, Harman CR. Problems in the detection of intrapartum fetal asphyxia with intermittent auscultation. Aust N Z J Obstet Gynaecol. 1999;39(2):218-22. | Not relevant information (4 cases reported) |
|  | Midirs. Fetal heart rate monitoring in labour. Bristol: Midirs; 2008. p. 5th ed. 6. | Unable to retrieve full-text |
|  | Miller DA. Fetal heart rate monitoring and the cesarean delivery rate. Contemporary OB/GYN. 2012;57(9):48-52. | Review |
|  | Miller DA. FHR monitoring as a screening test. Contemporary OB/GYN. 2012;57(11):54-9. | Review |
|  | Miller DA. Intrapartum fetal heart rate definitions and interpretation: evolving consensus. Clin Obstet Gynecol. 2011;54(1):16-21. | Not relevant information |
|  | Miller DA. Intrapartum fetal heart rate monitoring: a standardized approach to management. Clin Obstet Gynecol. 2011;54(1):22-7. | Expert opinion |
|  | Miller LA. Listen Carefully: Implementing Intermittent Auscultation Into Routine Practice. J Perinat Neonatal Nurs. 2015;29(3):197-9. | Not relevant information |
|  | Miller LA. The More Things Change, the More They Stay the Same: Thirty Years of Fetal Monitoring in Perspective. J Perinat Neonatal Nurs. 2016;30(3):255-8. | Review |
|  | Mitchell K. The effect of the labour electronic fetal monitoring admission test on operative delivery in low-risk women: a randomised controlled trial. Evidence Based Midwifery. 2008;6(1):18-26 | Not relevant information |
|  | Mitka M. Group issues revised guideline for fetal heart rate monitoring during labor. JAMA - Journal of the American Medical Association. 2009;302(9):935-6. | Commentary |
|  | M'Keever T. on the information afforded by the stethoscope. in detecting the presence of fœtal life. The Lancet. 1833;20(522):715-7. | Not relevant information |
|  | Montagu S. In defence of the Pinard. Midwifery Matters. 2008(118):3-4. | Not relevant information |
|  | Montagu S. Observations, examinations and auscultation in different positions. Midwifery Matters. 2012(132):9-11. | Not relevant information |
|  | Morrison JC, Chez BF, Davis ID, Martin RW, Roberts WE, Martin JN, Jr., et al. Intrapartum fetal heart rate assessment: monitoring by auscultation or electronic means. Am J Obstet Gynecol. 1993;168(1 Pt 1):63-6. | Not relevant information |
|  | Morton F. Listen in with mother. Midwifery Matters. 2010(125):12-4. | Review |
|  | Munro J, Ford H, Scott A, Furnival E, Andrews S, Grayson S. Action research project responding to midwives' views of different methods of fetal monitoring in labour. MIDIRS Midwifery Digest. 2002;12(4):495-8. | Not relevant information |
|  | Murphy-Black T. RCM supplement--fetal monitoring in labour. Nurs Times. 1991;87(28):58-9. | Not relevant information |
|  | NAACOG. Nursing responsibilities in implementing intrapartum fetal heart rate monitoring. NAACOG 1988;15(11):suppl 1-2. | Newsletter |
|  | National Institute for H, Care E. Intrapartum care. London: NICE. 2015. | Information folder |
|  | Nelson J. Pinards: essential or outdated? Practising Midwife. 2010;13(6):46. | Commentary |
|  | Nettalk. Using a pinard. Midwifery Matters. 2009(123):35-6. | Unable to retrieve full-text |
|  | Neuman MR. Fetal monitoring techniques. CRC Crit Rev Bioeng. 1975;2(2):132-48. | Not relevant information |
|  | Nijhuis JG, van den Berg PP. Fysische diagnostiek – auscultatie van foetale harttonen [Physical diagnostics--auscultation of fetal heart sounds]. Ned Tijdschr Geneeskd. 1999;143(9):455-8. | Review |
|  | Nochimson DJ, Cetrulo CL. Intrapartum fetal monitoring. J Fam Pract. 1974;1(3-4):4-9. | Not relevant information |
|  | Nordahl T. Jordmorstetoskopet, et nyttig arbeidsredskap eller en museumsgjenstand? (The midwife stethoscope, a usefull tool or a museum object?) Tidsskrift for jordmødre. 2004;110(8):22-3. | Commentary |
|  | Okosun H, Arulkumaran S. Intrapartum fetal surveillance. Current Obstetrics and Gynaecology. 2005;15(1):18-24. | Review |
|  | Paine LL, Payton RG, Johnson TR. Auscultated fetal heart rate accelerations. Part I. Accuracy and documentation. J Nurse Midwifery. 1986;31(2):68-72. | Not relevant, antepartum care |
|  | Palmrich AH, Reinold E. Überwachung des Fötus während der Geburt und Indikation zur Geburtsbeendigung (Monitoring of the fetus during labor and indication for delivery termination). Wien Klin Wochenschr. 1968;80(20):381-4. | Not relevant information |
|  | Parer JT. The Dublin Trial of fetal heart rate monitoring: the final word? Birth: Issues in Perinatal Care. 1986;13(2):119-21. | Not relevant information |
|  | Pasko DN, Blanchard CT, Szychowski JM, Mbah R, Welty E, Harper LM, et al. Use of a novel device (Moyo) for intrapartum fetal monitoring in 1,000 consecutive pregnancies in Cameroon, Africa. American Journal of Obstetrics and Gynecology. 2018;218 (1 Supplement 1):S524 | Not relevant information |
|  | Paterno MT, McElroy K, Regan M. Electronic Fetal Monitoring and Cesarean Birth: A Scoping Review. Birth-Issues in Perinatal Care. 2016;43(4):277-84 | Review |
|  | Paul RH. Intrapartum fetal monitoring: current status and the future. Obstet Gynecol Surv. 1973;28(6):suppl:453-9. | Review |
|  | Pinkerton JH. Fetal auscultation--some aspects of its history and evolution. Ir Med J. 1976;69(14):363-8. | Not relevant information |
|  | Pipe NGJ. Intrapartum fetal monitoring. British Journal of Clinical Equipment. 1979;4(6):216-23. | Not relevant, about EFM only |
|  | Prentice A, Lind T. Fetal heart rate monitoring during labour--too frequent intervention, too little benefit? Lancet. 1987;2(8572):1375-7. | Review |
|  | Project looks to boost skills for better fetal monitoring. Midwives. 2011(4):8. | Not scientific article |
|  | Riffle EM. Fetal Heart Rate Assessment Best Practice. International Journal of Childbirth Education. 2014;29(4):55-8. | Review |
|  | Rimbach E, Sigg W. [The frequency of the fetal heart tones during uncomplicated birth] . Zentralbl Gynakol. 1967;89(1):34-40. | Not relevant information |
|  | Robinson C MS. Ask away. Essentially MIDIRS. 2010;1(1):37. | Not scientific article |
|  | Rosser J. Continuous electronic fetal heart monitoring during labour. Practising Midwife. 1998;1(7-8):60-1. | Review |
|  | Rossignol M, Chaillet N, Boughrassa F, Moutquin JM. Interrelations between four antepartum obstetric interventions and cesarean delivery in women at low risk: a systematic review and modeling of the cascade of interventions. Birth. 2014;41(1):70-8. | Review |
|  | Rossignol M, Moutquin JM, Boughrassa F, Bedard MJ, Chaillet N, Charest C, et al. Preventable obstetrical interventions: how many caesarean sections can be prevented in Canada? Journal of Obstetrics & Gynaecology Canada: JOGC. 2013;35(5):434-43. | Not relevant information |
|  | Royal College of Midwives, 2012. Evidence based guidelines for midwifery-led care in labour. | Guidelines from RCM |
|  | Saadia Z. Rates and indicators of Continuous Electronic fetal monitoring - A study from Saudi Arabia. Int J Health Sci (Qassim). 2015;9(1):3-8. | Not relevant information |
|  | Sandin-Bojo AK, Hall-Lord ML, Axelsson O, Larsson BW. Intrapartal care in a Swedish maternity unit after a quality-improvement programme. Midwifery. 2007;23(2):113-22. | Review |
|  | Sandmire HF, DeMott RK. Auscultation of the fetal heart presents advantages over electronic monitoring. Wis Med J. 1995;94(12):661-3. | Local guideline |
|  | Sandmire HF. Whither electronic fetal monitoring? Obstet Gynecol. 1990;76(6):1130-4. | Commentary |
|  | Saugstad OD, Soll RF. Assessing Heart Rate at Birth: Auscultation is Still the Gold Standard. Neonatology. 2016;110(3):238-40. | Not relevant, post-natal care |
|  | Schifrin BS, Amsel J, Burdorf G. The accuracy of auscultatory detection of fetal cardiac decelerations: a computer simulation. Am J Obstet Gynecol. 1992;166(2):566-76. | Not relevant information |
|  | Schifrin BS. The fetal monitoring polemic. Clin Perinatol. 1982;9(2):399-408. | Commentary |
|  | Schneider H. Minimal requirements of fetal monitoring during labor and delivery. Gynakologe. 1999;32(1):13-9. | Review |
|  | Schrock A. [Value and significance of obstetric monitoring methods]. Wien Klin Wochenschr. 1988;100(5):145-53. | Not relevant information |
|  | Schwartz N, Young BK. Intrapartum fetal monitoring today. J Perinat Med. 2006;34(2):99-107. | Review |
|  | Scupholme A, McLeod AG, Robertson EG. A birth center affiliated with the tertiary care center: comparison of outcome. Obstet Gynecol. 1986;67(4):598-603. | Not relevant information |
|  | Seymour J. Fetal monitoring. Nurs Times. 1996;91(51):47-8. | Commentary |
|  | Sheth SS, Malpani AN. Inappropriate use of new technology: impact on women's health. Int J Gynaecol Obstet. 1997;58(1):159-65. | Not relevant information |
|  | Sheth Thakkar S, Lammers S, Hahn PM, Waddington A. The Use of Intermittent Auscultation in Parturients of Varying BMI Categories: Experience From a Mid-Sized Tertiary Care Obstetrical Unit. Journal of Obstetrics & Gynaecology Canada: JOGC. 2015;37(4):310-3. | Not relevant information |
|  | Sholapurkar SL. Amendments in electronic fetal monitoring and intermittent auscultation. British Journal of Midwifery. 2016;24(9):665-7. | Commentary |
|  | Sholapurkar SL. Intermittent Auscultation in Labor: Could It Be Missing Many Pathological (Late) Fetal Heart Rate Decelerations? Analytical Review and Rationale for Improvement Supported by Clinical Cases. Journal of Clinical Medicine Research. 2015;7(12):919-25. | Review |
|  | Sholapurkar SL. Intermittent auscultation of fetal heart rate during labour - a widely accepted technique for low risk pregnancies: but are the current national guidelines robust and practical? Journal of Obstetrics & Gynaecology. 2010;30(6):537-40. | Review |
|  | Sholapurkar SL. Trace display Doppler monitors: The new Doppler monitors that display fetal heart rate trace and provide archive record are set to be widely adopted. This article debates the benefits of these additional functions in midwifery practice. British Journal of Midwifery. 2017;25(5):282-3. | Commentary |
|  | Shy KK, Luthy DA, Bennett FC, Whitfield M, Larson EB, van Belle G, et al. Effects of electronic fetal-heart-rate monitoring, as compared with periodic auscultation, on the neurologic development of premature infants. N Engl J Med. 1990;322(9):588-93. | Same study as Luthy DA et al. 1987 which is included |
|  | Shy KK, Olshan AF, Hickok DE, et al. Electronic fetal monitoring during premature labor and the occurrence of perinatal mortality in very low birthweight infants. Birth. 1988;15(1):14-8. | Not relevant information |
|  | Smith H. Maintaining the passion for pinards. Practising Midwife. 2013;16(8):26, 8-9. | Commentary |
|  | Smith JF, Jr., Onstad JH. Assessment of the fetus: intermittent auscultation, electronic fetal heart rate tracing, and fetal pulse oximetry. Obstet Gynecol Clin North Am. 2005;32(2):245-54. | Review |
|  | Snelgrove-Clarke E, Davies B, Flowerdew G, Young D. Implementing a Fetal Health Surveillance Guideline in Clinical Practice: A Pragmatic Randomized Controlled Trial of Action Learning. Worldviews Evid Based Nurs. 2015;12(5):281-8. | Not relevant information |
|  | Snelgrove-Clarke EE. The Effects of Action Learning on Nurses' Use of a Fetal Health Surveillance Guideline with Low-Risk Labouring Women: McGill University (Canada); 2010. | Not relevant information (Dissertation) |
|  | Snydal SH. Methods of fetal heart rate monitoring during labor: a selective review of the literature. J Nurse Midwifery. 1988;33(1):4-14. | Review |
|  | Sprague AE, Oppenheimer L, McCabe L, Brownlee J, Graham ID, Davies B, et al. The Ottawa Hospital's Clinical Practice Guideline for the Second Stage of Labour. Journal of Obstetrics & Gynaecology Canada: JOGC. 2006;28(9):769-79. | Regional guidelines |
|  | Steer PJ. Fetal distress. Current Obstetrics and Gynaecology. 2002;12(1):15-21. | Review |
|  | Strong TH, Jr., Jarles DL. Intrapartum auscultation of the fetal heart rate. Am J Obstet Gynecol. 1993;168(3 Pt 1):935-6. | Not relevant information |
|  | Supplee RB, Vezeau TM. Continuous electronic fetal monitoring: does it belong in low-risk births? MCN: The American Journal of Maternal Child Nursing. 1996;21(6):301-6. | Review |
|  | Sureau C. Historical perspectives: forgotten past, unpredictable future. Baillieres Clinical Obstetrics & Gynaecology. 1996;10(2):167-84. | Review |
|  | Tallon RW. Technology assessment. Electronic fetal monitoring. Nurs Manage. 1996;27(6):49-51. | Review |
|  | Teh WT, Tong S. Intrapartum fetal monitoring. Yesterday, today and tomorrow. O & G. 2009;11(4):36-7. | Review |
|  | Thacker SB, Stroup DF, Peterson HB. Efficacy and safety of intrapartum electronic fetal monitoring: an update. Obstet Gynecol. 1995;86(4 Pt 1):613-20. | Review |
|  | Thacker SB. The efficacy of intrapartum electronic fetal monitoring. Am J Obstet Gynecol. 1987;156(1):24-30. | Review |
|  | Thomas J, Paranjothy S, Kelly T, Kavanagh J. Cardiotocography v Doppler auscultation. Guidelines highlight gaps in research evidence. BMJ. 2002;324(7335):482-5. | Letter to the editor |
|  | Thompson H. Promoting normal birth: Appropriate use of intrapartum cardiotocography. British Journal of Midwifery. 2011;19(10):625-9. | Commentary |
|  | Tillett J. Expert opinion: perinatal. Intermittent auscultation of the fetal heartbeat: can nurses change the culture of technology? J Perinat Neonatal Nurs. 2007;21(2):80-2. | Expert opinion |
|  | Tillett J. Intermittent auscultation of the fetal heartbeat: can nurses change the culture of technology? J Perinat Neonatal Nurs. 2007;21(2):80-2. | Commentary |
|  | Torpin R. Modification of Hillis-DeLee obstetric stethoscope. Am J Obstet Gynecol. 1938;36(6):1073. | Historical review |
|  | Torres J, De Vries R, Low LK. Consumer information on fetal heart rate monitoring during labor: a content analysis: a content analysis. J Perinat Neonatal Nurs. 2014;28(2):135-43. | Not relevant information |
|  | Tranquilli AL. Fetal heart rate in the second stage of labor: recording, reading, interpreting and acting. J Matern Fetal Neonatal Med. 2012;25(12):2551-4. | Review |
|  | Tucker JM, Hauth JC. Intrapartum assessment of fetal well-being. Clin Obstet Gynecol. 1990;33(3):515-25. | Review |
|  | Ubhi J. From Pinard to cardiotocography: modern fetal heart monitoring at home and in hospital. Prof Care Mother Child. 1995;5(4):96-8. | Not relevant, antenatal care |
|  | Ugwumadu A. Are we (mis)guided by current guidelines on intrapartum fetal heart rate monitoring? Case for a more physiological approach to interpretation. BJOG. 2014;121(9):1063-70. | Not relevant information |
|  | van Geijn HP. Intrapartum fetal heart rate monitoring. International Congress Series2005. p. 332-7. | Review |
|  | Vintzileos AM, Nochimson DJ, Guzman ER, Knuppel RA, Lake M, Schifrin BS. Intrapartum electronic fetal heart rate monitoring versus intermittent auscultation: a meta-analysis. Obstet Gynecol. 1995;85(1):149-55. | Review |
|  | Vintzileos AM, Nochimson DJ, Guzman ER, Knuppel RA, Lake M, Schifrin BS. Intrapartum electronic fetal heart-rate monitoring versus intermittent auscultation - a metaanalysis. Obstet Gynecol. 1995;85(1):149-55. | Review |
|  | Walker DS, Shunkwiler S, Supanich J, Williamsen J, Yensch A. Labor and delivery nurses' attitudes toward intermittent fetal monitoring. J Midwifery Womens Health. 2001;46(6):374-80. | Not relevant information |
|  | Westerhuis MEMH, Strasser SM, Moons KGM, Mol BWJ, Visser GHA, Kwee A. Foetale bewaking intra partum: van stethoscoop naar ST-analyse van het ecg (Intrapartum fetal monitoring: From stethoscope to ST analysis of the ECG). Ned Tijdschr Geneeskd. 2009;153(26):1278-83. | Review |
|  | Westin B, Huldt L. Fetal heart rate variation during uncomplicated labour. Acta Obstet Gynecol Scand. 1971;50(3):197-202. | Not relevant information |
|  | Wickham S. Pinard wisdom. Tips and tricks from midwives ( Part 2). Practising Midwife. 2002;5(10):35. | Review |
|  | Wickham S. Pinard wisdom. Tips and tricks from midwives (Part 1). Practising Midwife. 2002;5(9):21. | Review |
|  | Wickham S. ReView: cardiotocography intermittent auscultation of fetal hart on admission to labour ward for assessment of fetal wellbeing. Essentially MIDIRS. 2012;3(6):27-31. | Commentary |
|  | Wisner K. Intermittent auscultation in low-risk labor. MCN, American Journal of Maternal Child Nursing. 2015;40(1):58. | Commentary |
|  | Wolff F. Standards in fetal monitoring.  [German]. Z Geburtshilfe Neonatol. 1997;201(1):1-5. | Not relevant information |
|  | Wood SH. Should women be given a choice about fetal assessment in labor? MCN, American Journal of Maternal Child Nursing. 2003;28(5):292-8; quiz 9-300. | Not relevant information |
|  | Woods D. Appropriate technology and education for improved intrapartum care in under-resourced countries. S Afr J Obstet Gynaecol. 2009;15(3):78-9. | Not relevant information |
|  | Worku AG, Yalew AW, Afework MF. Availability and components of maternity services according to providers and users perspectives in North Gondar, Northwest Ethiopia. Reprod Health. 2013;10:43. | Not relevant information |
|  | Wrightson J. Cardiotocography versus intermittent auscultation. Using pinnards and Dopplers in low risk women. Practising Midwife. 2002;5(7):35-9. | Review |
|  | Zwerling B, Hoffmann SW, Savitsky LM, Caughey AB. Cost-effectiveness of continuous intrapartum electronic fetal monitoring vs intermittent auscultation in low-risk term pregnancies. Am J Obstet Gynecol. 2017;216 (1 Supplement 1):S385-S6. | Not relevant information |
