# Supplementary material for: Intermittent auscultation fetal monitoring during labour: A systematic scoping review to identify methods, effects, and accuracy
Source: PLoS One. 2019 Jul 10;14(7):e0219573. doi: 10.1371/journal.pone.0219573 (PMC6619817; doi:10.1371/journal.pone.0219573)
Supplement: S3 Table — A detailed description of devices used; the frequency, timing, and duration of IA; definitions of normal and abnormal FHRs; and additional observations performed together with auscultation. (DOCX) [file pone.0219573.s003.docx]

**S3 Table. Detailed descriptions of devices and modes of performing IA from the included articles**

| **Smith V, Begley C, Newell J, Higgins S, Murphy DJ, White MJ, Morrisin JJ, Canny S, O’Donovan D, Devane D. Admission cardiotocography versus intermittent auscultation of the fetal heart in low-risk pregnancy during evaluation for possible labour admission - a multicentre randomised trial: the ADCAR trial. BJOG: An International Journal of Obstetrics & Gynaecology. 2019;126:114-21**  **Ireland**  **RCT: Admission CTG vs auscultation at admission** | | | |
| --- | --- | --- | --- |
| Device(s) used for IA | Pinard or Doppler device, Doppler device most common | | |
| Frequency of auscultation 1^st^ stage | At least every 15 minute | | |
| Frequency of auscultation 2^nd^ stage | At least every 5 minute | | |
| Timing of auscultation | After a contraction | | |
| Duration of auscultation | At least 60 seconds | | |
| Description of FHR | Not described | | |
| Definition normal FHR | Not described | | |
| Definition abnormal FHR | Baseline FHR <110 bpm or >160 bpm or any deceleration | | |
| Rhythm | Not described | | |
| Assessment uterine contractions | Not described | | |
| Assessment fetal movements | Not described | | |
| Other | - | | |
| **Kamala BA, Ersdal HL, Dalen I, Abeid MS, Ngarina MM, Perlman JM, et al. Implementation of a novel continuous fetal Doppler (Moyo) improves quality of intrapartum fetal heart rate monitoring in a resource-limited tertiary hospital in Tanzania: An observational study. PLoS ONE. 2018;13(10):e0205698.**  **Tanzania**  **Pre- and post-intervention study** | | | |
| Device(s) used for IA | Pinard | | |
| Frequency of auscultation 1^st^ stage | 30 minutes | | |
| Frequency of auscultation 2^nd^ stage | 15 minutes | | |
| Timing of auscultation | Immediately after a contraction | | |
| Duration of auscultation | one complete minute | | |
| Description of FHR | Not described | | |
| Definition normal FHR | Not described | | |
| Definition abnormal FHR | FHR <120 or >160 | | |
| Rhythm | Not described | | |
| Assessment uterine contractions | Not described | | |
| Assessment fetal movements | Not described | | |
| Other | The study states that “The Pinard is unable to delineate either decelerations or accelerations” (p 5/14) | | |
| **Maude RM, Skinner JP, Foureur MJ. Intelligent Structured Intermittent Auscultation (ISIA): evaluation of a decision-making framework for fetal heart monitoring of low-risk women. BMC Pregnancy & Childbirth. 2014;14:184.**  **NZ**  **Mixed method intervention study**  **Maude R, Foureur M. Intrapartum fetal heart rate monitoring: using audit methodology to identify areas for research and practice improvement. New Zealand College of Midwives Journal. 2009(40):24-30.**  **NZ**  **Retrospective audit** | | | |
| Device(s) used for IA | Pinard  Hand-held Doppler device | | |
| Frequency of auscultation 1^st^ stage | 15-30 minutes | | |
| Frequency of auscultation 2^nd^ stage | 5 minutes or after each contraction | | |
| Timing of auscultation | Between contractions and during fetal movements to assess baseline FHR and immediately after contractions to detect decelerations. | | |
| Duration of auscultation | 30-60 seconds, 60 seconds optimal | | |
| Description of FHR | Single figure | | |
| Definition normal FHR | Average FHR 110-160, presence of FHR increases of at least 15 bpm (accelerations), absence of FHR decreases (decelerations), rhythm regular, in combination with normal uterine activity and tone. | | |
| Definition abnormal FHR | Average FHR <110 or >160, absence of FHR increases (accelerations), gradual or abrupt FHR decrease (decelerations), rhythm irregular, excessive uterine activity or increased tenderness or tone. | | |
| Rhythm | Regular or irregular. | | |
| Assessment uterine contractions | Determine onset, duration, frequency and intensity of contractions. Note any uterine irritability or tenderness and uterine resting tone between contractions | | |
| Assessment fetal movements | Ask the woman about the pattern of recent fetal movement, palpate fetal movements together with the woman, record each time a fetal movement was felt. Count FHR during fetal movements (for FHR increases) | | |
| Other | Palpate maternal pulse to differentiate from FHR.  One-to-one care must be possible. | | |
| **Rathore AM, Ramji S, Devi CB, Saini S, Manaktala U, Batra S. Fetal scalp stimulation test: an adjunct to intermittent auscultation in non-reassuring fetal status during labor. J Obstet Gynaecol Res. 2011;37(7):819-24.**  **India, New Dehli**  **Prospective observational study, to evaluate fetal scalp stimulation test as an adjunct to IA** | | | |
| Device(s) used for IA | “Stethoscope” (not specified) | | |
| Frequency of auscultation 1^st^ stage | 15 minutes | | |
| Frequency of auscultation 2^nd^ stage | 5 minutes | | |
| Timing of auscultation | After a contraction | | |
| Duration of auscultation | 30 seconds | | |
| Description of FHR | Not described | | |
| Definition normal FHR | Not described | | |
| Definition abnormal FHR | Not described | | |
| Rhythm | Not described | | |
| Assessment uterine contractions | Not described | | |
| Assessment fetal movements | Not described | | |
| Other | - | | |
| Soltani MA. Integrating a hand held computer and stethoscope into a fetal monitor. Med. 2009;14:3.  Iran  Development of a new device for IA, a hand-held computer or personal digital assistent | | | |
| Device(s) used for IA | Personal Digital Assistant, an electronic stethoscope (stethoscope attached to a hand-held computer). | | |
| Frequency of auscultation 1^st^ stage | Not described | | |
| Frequency of auscultation 2^nd^ stage | Not described | | |
| Timing of auscultation | Not described | | |
| Duration of auscultation | Not described | | |
| Description of FHR | Not described | | |
| Definition normal FHR | Not described | | |
| Definition abnormal FHR | Not described | | |
| Rhythm | Not described | | |
| Assessment uterine contractions | Not described | | |
| Assessment fetal movements | Not described | | |
| Other | A new electronic device for fetal heart auscultation is presented, where a stethoscope is attached to a hand-held computer. Two models are shown, one with a stethoscope attached to a cell phone, and another with a Pinard attached to an MP3-player.  An electronic device will not emit any energy into the woman’s body. | | |
| **Madaan M, Trivedi SS. Intrapartum electronic fetal monitoring vs. intermittent auscultation in postcesarean pregnancies. International Journal of Gynaecology & Obstetrics. 2006;94(2):123-5.**  **India**  **RCT: EFM vs. IA in women with postcaesarean pregnancies** | | | |
| Device(s) used for IA | Not described | | |
| Frequency of auscultation 1^st^ stage | 15 minutes | | |
| Frequency of auscultation 2^nd^ stage | 5 minutes | | |
| Timing of auscultation | During and immediately after a contraction | | |
| Duration of auscultation | 60 seconds | | |
| Description of FHR | Not described | | |
| Definition normal FHR | Not described | | |
| Definition abnormal FHR | Persistent fetal tachycardia or bradycardia; irregular FHR; and FHR during and immediately after contraction repeatedly below 100 bpm. | | |
| Rhythm | Not described | | |
| Assessment uterine contractions | By palpation | | |
| Assessment fetal movements | Not described | | |
| Other | - | | |
| **Impey L, Reynolds M, MacQuillan K, Gates S, Murphy J, Sheil O. Admission cardiotocography: a randomised controlled trial. Lancet. 2003;361(9356):465-70.**  **Ireland**  **RCT: Admission CTG vs auscultation at admission** | | | |
| Device(s) used for IA | Not described | | |
| Frequency of auscultation 1^st^ stage | 15 min | | |
| Frequency of auscultation 2^nd^ stage | 5 min | | |
| Timing of auscultation | After a contraction | | |
| Duration of auscultation | 60 seconds | | |
| Description of FHR | Not described | | |
| Definition normal FHR | Not described | | |
| Definition abnormal FHR | Not described | | |
| Rhythm | Not described | | |
| Assessment uterine contractions | Not described | | |
| Assessment fetal movements | Not described | | |
| Other | - | | |
| **Mires G, Williams F, Howie P. Randomised controlled trial of cardiotocography versus Doppler auscultation of fetal heart at admission in labour in low risk obstetric population. BMJ. 2001;322(7300):1457-60.**  **RCT: Admission CTG vs auscultation at admission** | | | |
| Device(s) used for IA | Handheld Doppler device | | |
| Frequency of auscultation 1^st^ stage | Not described | | |
| Frequency of auscultation 2^nd^ stage | Not described | | |
| Timing of auscultation | During and immediately after at least one contraction | | |
| Duration of auscultation | Not described | | |
| Description of FHR | Not described | | |
| Definition normal FHR | Not described | | |
| Definition abnormal FHR | Not described | | |
| Rhythm | Not described | | |
| Assessment uterine contractions | Not described | | |
| Assessment fetal movements | Not described | | |
| Other | - | | |
| **Gilles MT, Norman M, Dawes V, Gee V, Rouse I, Newnham J. Intermittent auscultation for the intrapartum assessment of fetal well-being in Western Australia. Australian & New Zealand Journal of Obstetrics & Gynaecology. 1997;37(2):143-8.**  **Australia**  **Survey the use of IA throughout maternity units in Western Australia** | | | |
| Device(s) used for IA | Not described | | |
| Frequency of auscultation 1^st^ stage | 30 minutes in low risk women; 15-30 minutes in women with higher risk | | |
| Frequency of auscultation 2^nd^ stage | 15 minutes in low risk women; 5-15 minutes and after most contractions in women with higher risk | | |
| Timing of auscultation | Not described | | |
| Duration of auscultation | Not described | | |
| Description of FHR | Not described | | |
| Definition normal FHR | Not described | | |
| Definition abnormal FHR | If the fetal heart rate is >160 bpm over 3 consecutive contractions (in the absence of a fever), <100 bpm after 3 consecutive contractions, or there is persisting bradycardia of more than 2 minutes duration. | | |
| Rhythm | Not described | | |
| Assessment uterine contractions | Not described | | |
| Assessment fetal movements | Not described | | |
| Other | - | | |
| **Vintzileos AM, Antsaklis A, Varvarigos I, Papas C, Sofatzis I, Montgomery JT. A randomized trial of intrapartum electronic fetal heart rate monitoring versus intermittent auscultation. Obstet Gynecol. 1993;81(6):899-907.**  **Greece**  **RCT: EFM vs. IA** | | | |
| Device(s) used for IA | Doppler device (Sonicaid Limited D206, Oxford, England) | | |
| Frequency of auscultation 1^st^ stage | 15 minutes | | |
| Frequency of auscultation 2^nd^ stage | 5 minutes | | |
| Timing of auscultation | Between, during and immediately after a contraction | | |
| Duration of auscultation | 60 seconds – at least 30 seconds after a contraction | | |
| Description of FHR | Not described | | |
| Definition normal FHR | See under | | |
| Definition abnormal FHR | The presence of one or more of the following: 1) FHR during and immediately after a contraction repeatedly below 100 beats per min, even if there was a recovery to 120-160 before the next deceleration (moderate decelerations when the FHR was between 80-99 beats per min and severe when the FHR was less than 100 beats per min); 2) persistent baseline rate (between contractions) of more than 160 or less than 100 bpm | | |
| Rhythm | Not described | | |
| Assessment uterine contractions | Evaluated by palpation | | |
| Assessment fetal movements | Not described | | |
| Other | - | | |
| **Luthy DA, Shy KK, van Belle G, Larson EB, Hughes JP, Benedetti TJ, et al. A randomized trial of electronic fetal monitoring in preterm labor. Obstet Gynecol. 1987;69(5):687-95.**  **USA and Canada (Washington stte and BC)**  **RCT: EFM vs. IA in preterm single infants** | | | |
| Device(s) used for IA | DeLee fetoscope or Doppler device | | |
| Frequency of auscultation 1^st^ stage | 15 minutes | | |
| Frequency of auscultation 2^nd^ stage | 5 minutes | | |
| Timing of auscultation | Immediately after a contraction  Baseline FHR obtained between contractions | | |
| Duration of auscultation | At least 30 seconds | | |
| Description of FHR | Not described | | |
| Definition normal FHR | (Auscultated FHRs were classified as reassuring or ominous.)  Reassuring: FHR 120-160 throughout the first and second stages of labour. | | |
| Definition abnormal FHR | Ominous: FHR less than 100 persisting more than 30 sec after 3 or more consecutive contractions, or a baseline FHR > 180 for more than 15 min, or < 100 more than 60 sec. | | |
| Rhythm | Not described | | |
| Assessment uterine contractions | By palpation | | |
| Assessment fetal movements | Not described | | |
| Other | One-to-one care | | |
| **Neldam S, Osler M, Hansen PK, Nim J, Smith SF, Hertel J. Intrapartum fetal heart rate monitoring in a combined low- and high-risk population: a controlled clinical trial. European Journal of Obstetrics, Gynecology, & Reproductive Biology. 1986;23(1-2):1-11.**  **Denmark**  **RCT: EFM vs. IA** | | | |
| Device(s) used for IA | “Stethoscope” | | |
| Frequency of auscultation 1^st^ stage | Up to 5 cm cervical dilatation: 30 minutes  From 5 cm dilatation: 15 minutes | | |
| Frequency of auscultation 2^nd^ stage | After each contraction or every 5 minutes | | |
| Timing of auscultation | 1^st^ stage: not described  2^nd^ stage: after a contraction | | |
| Duration of auscultation | 1^st^ stage: at least 15 seconds  2^nd^ stage 30 seconds | | |
| Description of FHR | Not described | | |
| Definition normal FHR | Normal baseline 120-160 | | |
| Definition abnormal FHR | FHR baseline < 120 or > 160. FHR <100 after three or more consecutive contractions. | | |
| Rhythm | Not described | | |
| Assessment uterine contractions | Not described | | |
| Assessment fetal movements | Not described | | |
| Other | - | | |
| **MacDonald D, Grant A, Sheridan-Pereira M, Boylan P, Chalmers I. The Dublin randomized controlled trial of intrapartum fetal heart rate monitoring. American Journal of Obstetrics & Gynecology. 1985;152(5):524-39.**  **Ireland**  **EFM vs. IA** | | | |
| Device(s) used for IA | Pinard | | |
| Frequency of auscultation 1^st^ stage | At least every 15 minutes | | |
| Frequency of auscultation 2^nd^ stage | Every interval between contractions | | |
| Timing of auscultation | 1^st^ stage: following a contraction  2^nd^ stage: during every interval | | |
| Duration of auscultation | 60 seconds | | |
| Description of FHR | Not described | | |
| Definition normal FHR | - | | |
| Definition abnormal FHR | FHR (baseline) >160 or <100 during three contractions | | |
| Rhythm | Not described | | |
| Assessment uterine contractions | Not described | | |
| Assessment fetal movements | Not described | | |
| Other | - | | |
| **Applegate J, Haverkamp AD, Orleans M, Taylor C. Electronic fetal monitoring: implications for obstetrical nursing. Nurs Res. 1979;28(6):369-71.**  **USA, Colorado**  **RCT, EFM vs. IA**  **Haverkamp AD, Thompson HE, McFee JG, Cetrulo C. The evaluation of continuous fetal heart rate monitoring in high-risk pregnancy. American Journal of Obstetrics & Gynecology. 1976;125(3):310-20.**  **Same study as above** | | | |
| Device(s) used for IA | Not described | | |
| Frequency of auscultation 1^st^ stage | 15 min | | |
| Frequency of auscultation 2^nd^ stage | 5 min | | |
| Timing of auscultation | After a contraction | | |
| Duration of auscultation | 30 seconds | | |
| Description of FHR | Not described | | |
| Definition normal FHR | FHR baseline 120-160 bpm. | | |
| Definition abnormal FHR | If FHR >120 or >160, auscultation after each contraction and even during contractions | | |
| Rhythm | Not described | | |
| Assessment uterine contractions | Not described | | |
| Assessment fetal movements | Not described | | |
| Other | - | | |
| **Haverkamp AD, Orleans M, Langendoerfer S, McFee J, Murphy J, Thompson HE. A controlled trial of the differential effects of intrapartum fetal monitoring. American Journal of Obstetrics & Gynecology. 1979;134(4):399-412.**  **USA, Colorado**  **RCT: 3 arms, IA, CTG alone and CTG+FBS** | | | |
| Device(s) used for IA | Not described | | |
| Frequency of auscultation 1^st^ stage | 15 minutes | | |
| Frequency of auscultation 2^nd^ stage | 5 minutes | | |
| Timing of auscultation | After a contraction | | |
| Duration of auscultation | 30 seconds | | |
| Description of FHR | Not described | | |
| Definition normal FHR | Not described | | |
| Definition abnormal FHR | Fetal distress described as FHR>100 bpm after 3 or more consecutive contractions despite corrective measures- | | |
| Rhythm | Not described | | |
| Assessment uterine contractions | Not described | | |
| Assessment fetal movements | Not described | | |
| Other | - | | |
| **Kelso IM, Parsons RJ, Lawrence GF, Arora SS, Edmonds DK, Cooke ID. An assessment of continuous fetal heart rate monitoring in labor. A randomized trial. American Journal of Obstetrics & Gynecology. 1978;131(5):526-32.**  **England**  **RCT: EFM vs. IA** | | | |
| Device(s) used for IA | Pinard; Doppler device (Sonicaid D205) if difficult to hear | | |
| Frequency of auscultation 1^st^ stage | 15 minutes, or more if indicated | | |
| Frequency of auscultation 2^nd^ stage | Not described | | |
| Timing of auscultation | During or immediately after a contraction | | |
| Duration of auscultation | 60 seconds | | |
| Description of FHR | Not described | | |
| Definition normal FHR | FHR 120-160 | | |
| Definition abnormal FHR | Not described | | |
| Rhythm | Not described | | |
| Assessment uterine contractions | Not described | | |
| Assessment fetal movements | Not described | | |
| Other | - | | |
| **Articles describing devices and modes of performing, and assessing the effect of different modes of IA** | | | |
| **Kamala BA, Wangwe PJ, Dalen I, Mduma E, Perlman JM, Ersdal HL. Intrapartum fetal heart rate monitoring using a handheld Doppler versus Pinard stethoscope: a randomized controlled study in Dar es Salaam. Int J Womens Health 2018(10):341-8.**  **Tanzania**  **RCT: Pinard vs. Freeplay handheld wind-up Doppler** | | | |
| Device(s) used for IA | Pinard fetoscope  Wind-up hand-held Doppler device (Power-free Education Technology) | | |
| Frequency of auscultation 1^st^ stage | 30 minutes | | |
| Frequency of auscultation 2^nd^ stage | 5-15 minutes | | |
| Timing of auscultation | The last 10 minutes of every half hour, particularly before, during and immediately after a contraction | | |
| Duration of auscultation | Duration of each counting not described | | |
| Description of FHR | Not described | | |
| Definition normal FHR | Not described | | |
| Definition abnormal FHR | <120 or >160 bpm | | |
| Rhythm | Not described | | |
| Assessment uterine contractions | Not described | | |
| Assessment fetal movements | Not described | | |
| Other | Effects of Doppler device vs. Pinard:  IA using Doppler device was associated with an increased detection of abnormal FHR compared to Pinard. There were no differences in neonatal outcomes or Caesarean section rates between the groups. | | |
| **Mdoe PF, Ersdal HL, Mduma ER, Perlman JM, Moshiro R, Wangwe PT, et al. Intermittent fetal heart rate monitoring using a fetoscope or hand held Doppler in rural Tanzania: a randomized controlled trial. BMC pregnancy and childbirth. 2018;18(1):134.**  **Tanzania**  **RCT: Pinard vs. Freeplay handheld wind-up Doppler** | | | |
| Device(s) used for IA | Pinard fetoscope  Wind-up hand-held Doppler device (Power-free Education Technology) | | |
| Frequency of auscultation 1^st^ stage | Not recorded; midwives were expected to auscultate every 30 minute | | |
| Frequency of auscultation 2^nd^ stage | Not recorded; midwives were expected to auscultate every 5-15 minute | | |
| Timing of auscultation | Not described | | |
| Duration of auscultation | Not described | | |
| Description of FHR | Not described | | |
| Definition normal FHR | 120-160 bpm | | |
| Definition abnormal FHR | <120 or >160 bpm, or absent | | |
| Rhythm | Not described | | |
| Assessment uterine contractions | Not described | | |
| Assessment fetal movements | Not described | | |
| Other | Effects of Doppler device vs. Pinard:  There were no differences between the groups regarding detection of abnormal FHR, neonatal outcomes or Caesarean section rates. | | |
| **Byaruhanga R, Bassani DG, Jagau A, Muwanguzi P, Montgomery AL, Lawn JE. Use of wind-up fetal Doppler versus Pinard for fetal heart rate intermittent monitoring in labour: a randomised clinical trial. BMJ Open. 2015;5(1):e006867.**  **Uganda**  **RCT: doppler vs. Pinard** | | | |
| Device(s) used for IA | Pinard fetoscope  Wind-up hand-held Doppler device (Power-free Education Technology) | | |
| Frequency of auscultation 1^st^ stage | 30 minutes | | |
| Frequency of auscultation 2^nd^ stage | 15 minutes before pushing  5 minutes during pushing | | |
| Timing of auscultation | Immediately after a contraction | | |
| Duration of auscultation | 60 seconds | | |
| Description of FHR | As a single number, in the unit of beats per minute. Absence/presence of decelerations were recorded. | | |
| Definition normal FHR | 110-160 bpm | | |
| Definition abnormal FHR | Abrupt variable deceleration; lasting >2 min with slow return to baseline or in the presence of tachycardia.  Late deceleration; repetetive gradual decrease in the FHR and return to baseline, starting after the onset of the contraction and return to baseline after the end of the contraction. Prolonged deceleration; decrease from baseline of > 15bpm lasting for 2-10 min | | |
| Rhythm | Regular or irregular | | |
| Assessment uterine contractions | Not described | | |
| Assessment fetal movements | Not described | | |
| Other | The maternal radial pulse simultaneously palpated to differentiate from FHR  Effects of Doppler device vs. Pinard:  IA using Doppler device was associated with an increased detection of abnormal FHR compared to Pinard. There were no differences in neonatal outcomes or Caesarean section rates. | | |
| **Mahomed K, Nyoni R, Mulambo T, Kasule J, Jacobus E. Randomised controlled trial of intrapartum fetal heart rate monitoring. BMJ. 1994;308(6927):497-500.**  **Zimbabwe**  **RCT: 4 arms, IA by external CTG, Doppler ultrasound by research mw, Pinard by research mw, routine monitoring** | | | |
| Device(s) used for IA | Huntleigh Pocket Ultrasound Doppler  Pinard | | |
| Frequency of auscultation 1^st^ stage | 30 minutes (1^st^ and 2^nd^ stage not separated) | | |
| Frequency of auscultation 2^nd^ stage | 30 minutes (1^st^ and 2^nd^ stage not separated) | | |
| Timing of auscultation | The last 10 minutes of every half hour, particularly during and immediately after a contraction. | | |
| Duration of auscultation | Not specified for each auscultation | | |
| Description of FHR | Not described | | |
| Definition normal FHR | Not described | | |
| Definition abnormal FHR | Not clearly described, but decelerations lasting >30 sec and persistent late decelerations were indications for caesarean section. | | |
| Rhythm | Not described | | |
| Assessment uterine contractions | Not described | | |
| Assessment fetal movements | Not described | | |
| Other | - | | |
| **Articles assessing the accuracy of IA** | | | |
| **Simpson N, Oppenheimer LW, Siren A, Bland E, McDonald O, McDonald D, et al. Accuracy of strategies for monitoring fetal heart rate in labor. American journal of perinatology. 1999;16(4):167-73.**  **Canada, Ottawa**  **Investigated if the accuracy of auscultation could be improved with a heart rate meter** | | | |
| Aim of study | To investigate if the accuracy of auscultation could be improved with the use of a heart rate meter | | |
| Methods | Six FHR traces representing a spectrum of rate and periodic changes were selected from a database. The six tracings were of 3 minutes duration and included a contraction. They were assessed as counting alone where the contraction was shown with a moving bar on the screen, as counting with the aid of a heart rate meter in addition (FHR was displayed on the screen, updated every 2 seconds), and as a CTG-trace without sound. 15 experienced nurses and 15 obstetric residents were asked to assess baseline rate, baseline variability, periodic changes and record the trace as reassuring or non-reassuring. | | |
| Results | Counting alone was associated with underestimation of FHR in 4/6 traces. The assessments were most correct when the CTG tracing was displayed. When the FHR assessments were performed by the aid of a heart rate meter, the assessments were significantly more accurate and it reduced intra-observer variation. | | |
| Device(s) used for IA | No device (digitalized FHR traces with sound), laboratory simulation study | | |
| Frequency of auscultation 1^st^ stage | Not relevant | | |
| Frequency of auscultation 2^nd^ stage | Not relevant | | |
| Timing of auscultation | Before, during and immediately after a contraction | | |
| Duration of auscultation | 15 seconds | | |
| Description of FHR | BPM by *4 using a multiplication table. The following were described: BPM, baseline variability (reduced (<6 bpm), normal (6-25) excessive (>25) or cannot assess. Periodic changes: not present, acceleration, deceleration with recovery by end of contraction, deceleration with recovery by end of contraction  Complete assessment: reassuring or non-reassuring. | | |
| Definition normal FHR | See above | | |
| Definition abnormal FHR | See above | | |
| Rhythm | Not described | | |
| Assessment uterine contractions | Not relevant | | |
| Assessment fetal movements | Not relevant | | |
| Other | Not a clinical study, performed in a setting where electronic fetal monitoring was the standard | | |
| **Strong TH, Jarles DL. Intrapartum auscultation of the fetal heart rate. Am J Obstet Gynecol 1992;168:935-6.**  **USA (Arizona)**  **Accuracy study** | | | |
| Aim of study | To evaluate current practice of auscultation on the detection of decelerations | | |
| Methods | A recording of an intrapartum FHR was played to an audience of 120 physicians and nurses at an obstetric conference. The recording was derived from an internal FHR monitor. The observers were instructed to estimate baseline FHR, FHR nadir and duration of the deceleration using any technique at their disposal. The group was informed that the recording included a variable deceleration | | |
| Results |  | N=120 | “Golden standard” |
|  | Baseline FHR (mean) | 140+10 bpm | 138 bpm |
|  | FHR nadir (mean) | 82+17 bpm | 90 bpm |
|  | Duration | 39+15 sec | 50 sec |
| Device(s) used for IA | No device, recording of intrapartum FHR | | |
| Frequency of auscultation 1^st^ stage | Not relevant | | |
| Frequency of auscultation 2^nd^ stage | Not relevant | | |
| Timing of auscultation | During a variable deceleration, and probably before and after | | |
| Duration of auscultation | Not described | | |
| Description of FHR | Baseline, nadir during deceleration, duration of deceleration | | |
| Definition normal FHR | Not described | | |
| Definition abnormal FHR | Not described | | |
| Rhythm | Not described | | |
| Assessment uterine contractions | Not relevant | | |
| Assessment fetal movements | Not relevant | | |
| Other | - | | |
| **Miller FC, Pearse KE, Paul RH. Fetal heart rate pattern recognition by the method of auscultation. Obstet Gynecol. 1984;64(3):332-6.**  **USA, California**  **Accuracy study, participants listened to audiotapes and described the sounds** | | | |
| Aim of study | To define precisely what characteristics of FHR and FHR patterns can be recognized by IA. | | |
| Methods | Eight classic FHR pattern were selected from a library of monitored labours recorded and stored on magnetic tape. The FHR patterns included 1) normal baseline, 2) nonuniform accelerations, 3) uniform accelerations, 4) saltatory pattern, 5) early decelerations, 6) variable decelerations, 7) late decelerations with good baseline variability and 8) late decelerations with diminished baseline variability. An audiotone was produced for each beat to simulate fetal heart tones. A segment of three minutes, including an entire contraction, was recorded with a verbal statement indicating the onset, peak and end of the contraction. 16 physicians and 16 nurses who used electronic FHR monitoring regularly, assessed the heart tones. The observers listened to the recordings and filled in a questionnaire about baseline, long-term variability, periodic changes related to contractions, and non-periodic changes not related to contractions. | | |
| Results | Baseline was correctly scored by 31/32 observers, accelerations without periodic changes (recording 2) by 23/32, non-uniform accelerations by 27/32, saltatory pattern by 8/32, early decelerations by 18/32, variable decelerations by 24/32, late decelerations with good variability by 20/32 and late decelerations with diminished variability by 26/32. | | |
| Conclusion | Baseline was most easy to score correct. Saltatory pattern was most difficult, and was often interpreted as tachycardia. One third of the observers failed to identify significant periodic patterns. | | |
| Device(s) used for IA | No device, recordings of FHR sounds | | |
| Timing of auscultation | Before, during and after a contraction | | |
| Duration of auscultation | Each segment was 3 minutes and included a contraction | | |
| Description of FHR | The observers were asked to answer questions about baseline, long-term variability, periodic changes in FHR related to contraction and non-periodic changes, not related to contractions | | |
| Definition normal FHR | Baseline 120-160, accelerations of 10-15 bpm, for 10-15 seconds | | |
| Definition abnormal FHR | Long term variability diminished = <5 bpm, <3 oscilations/min; average=5-20 bpm>3 oscilations/minute; saltatory or increased=>20 bpm >3 oscilations/minute. | | |
| Rhythm | Not described | | |
| Assessment uterine contractions | A verbal statement noting the onset, peak and the end of each contraction | | |
| Assessment fetal movements | Not described | | |
| Other | Not a clinical setting | | |
| **Day E, Maddern L, Wood C. Auscultation of foetal heart rate: an assessment of its error and significance. British Medical Journal. 1968;4(5628):422-4.**  **Australia**  **Accuracy study** | | | |
| Aim of study | To determine the accuracy and usefulness of clinical measurement of the FHR. | | |
| Methods | Clinical FHR was recorded by hospital staff (a trained midwife and a resident obstetrician) and by two medical students. The FHR was recorded electronically by a fetal scalp electrode to detect the fetal electrocardiograph. 126 observations were made on 90 women. Assessments of error of auscultation was determined by comparing the auscultations with the electronic monitoring. | | |
| Results | Three types of auscultation errors were described: random error, error biased towards normality when the FHR is fast or slow, and error based on inability to count FHR during contractions. The two medical students had fewer errors in determine the FHR compared to the midwife and the obstetrician. | | |
| Device(s) used for IA | Not described | | |
| Frequency of auscultation 1^st^ stage | - | | |
| Frequency of auscultation 2^nd^ stage | - | | |
| Timing of auscultation | Before, during and after a contraction | | |
| Duration of auscultation | Every 15 second, with 5 seconds intervals | | |
| Description of FHR | Range of FHR (130-150 bpm; <130 bpm; >150 bpm), | | |
| Definition normal FHR | FHR 120-160 bpm | | |
| Definition abnormal FHR | FHR <120 or >160 bpm | | |
| Rhythm | Not described | | |
| Assessment uterine contractions | - | | |
| Assessment fetal movements | - | | |
| Other | Auscultations performed in a clinical setting | | |
