# Supplementary material for: Intermittent auscultation fetal monitoring during labour: A systematic scoping review to identify methods, effects, and accuracy
Source: PLoS One. 2019 Jul 10;14(7):e0219573. doi: 10.1371/journal.pone.0219573 (PMC6619817; doi:10.1371/journal.pone.0219573)
Supplement: S4 Table — A detailed description of recommendations of devices; frequency, timing, and duration of IA; definitions of normal and abnormal FHRs; and additional observations performed together with auscultation. (DOCX) [file pone.0219573.s004.docx]

**S4 Table. Detailed descriptions of recommendations from the included guidelines**

| **World Health Organization, 2018**  World Health Organization. Intrapartum care for a positive childbirth experience. WHO, editor. Geneva: WHO; 2018. | |
| --- | --- |
| Recommendations: |  |
| Device(s) used for IA | A Doppler ultrasound device or a Pinard fetal stethoscope |
| Frequency of auscultation 1^st^ stage | 15-30 minutes |
| Frequency of auscultation 2^nd^ stage | 5 minutes |
| Timing of auscultation | during a contraction and continue for at least 30 seconds after the contraction |
| Duration of auscultation | At least 60 seconds |
| Description of FHR | As a single counted number in beats per min, and the presence or absence of accelerations and decelerations |
| Definition normal FHR | Normal baseline 110-160 bpm |
| Definition abnormal FHR | Not described |
| Rhythm | Not described |
| Assessment uterine contractions | Not described |
| Assessment fetal movements | Not described |
| Other observations | Not described |
| Other recommendations | Admission CTG is not recommended for low-risk women presenting in spontaneous labour.  Intermittent auscultation is recommended as monitoring for healthy pregnant women in labour.  Continuous cardiotocography is not recommended in healthy pregnant women undergoing spontaneous labour  If the FHR is not always normal, auscultation should be prolonged over at least three contractions |
| Target users | National and local public health policy-makers, implementers and managers of maternal and child health programmes, health care facility managers, non-governmental  organizations (NGOs), professional societies involved in planning and management of  maternal and child health services, health care professionals (including nurses, midwives, general medical practitioners and obstetricians), academic staff involved in training health care professionals |
| **International Federation of Gynecology and Obstetrics (FIGO), 2015**  Lewis D, Downe S, Panel FIFMEC. FIGO consensus guidelines on intrapartum fetal monitoring: Intermittent auscultation. International Journal of Gynaecology & Obstetrics. 2015;131(1):9-12. | |
| Recommendations |  |
| Device(s) used for IA | Pinard stethoscope, DeLee stethoscope or hand-held Doppler device |
| Frequency of auscultation 1^st^ stage | 15 minutes (during active phase) |
| Frequency of auscultation 2^nd^ stage | 5 minutes |
| Timing of auscultation | During a contraction and continue for at least 30 sec after the contraction |
| Duration of auscultation | 60 seconds |
| Description of FHR | The baseline FHR as a single counted number in beats per min, the presence or absence of accelerations and decelerations |
| Definition normal FHR | Normal baseline is 110-160 bpm |
| Definition abnormal FHR | Presence of repetitive or prolonged (>3 minute) decelerations |
| Rhythm | Not described |
| Assessment uterine contractions | Frequency of uterine contractions in 10 minutes |
| Assessment fetal movements | Presence or absence of fetal movements |
| Other observations | Maternal heart rate |
| Other recommendations | Intermittent auscultation should be recommended in all settings where CTG monitoring is not available.  Intermittent auscultation may be used for routine monitoring in settings where the recourses for CTG monitoring is available.  Before auscultation, a clear explanation should be given to the woman, and her consent obtained. |
| Target users | Not clearly described |
| **International Confederation of Midwives (ICM), 2017**  International Confederation of Midwives. Use of intermittent auscultation for assessment of foetal wellbeing during labour 2017. | |
| Recommendations |  |
| Device(s) used for IA | Pinard or fetoscope or Doppler ultrasound device |
| Frequency of auscultation 1^st^ stage | Not described |
| Frequency of auscultation 2^nd^ stage | Not described |
| Timing of auscultation | Not described |
| Duration of auscultation | Not described |
| Description of FHR | Not described |
| Definition normal FHR | Not described |
| Definition abnormal FHR | Not described |
| Rhythm | Not described |
| Assessment uterine contractions | Not described |
| Assessment fetal movements | Not described |
| Other observations | Not described |
| Other recommendations | Intermittent auscultation is recommended in women who are well and healthy and have a normal (physiological) labour. |
| Target users | All healthcare professionals attending labour and delivery |
| **Denmark, 2017**  Dansk Sælskab for Obstetrik og Gynækologi (Danish Association of Obstetrics and Gynaecology). Fosterovervågning under fødslen - indikationer (Intrapartum fetal monitoring - indications). 2017. | |
| Recommendations |  |
| Device(s) used for IA | Pinard or hand-held Doppler device |
| Frequency of auscultation 1^st^ stage | 15-30 minutes |
| Frequency of auscultation 2^nd^ stage | 5 minutes or after each contraction |
| Timing of auscultation | During and after each contraction |
| Duration of auscultation | At least 60 seconds, for at least 30 seconds after the contraction |
| Description of FHR | Bpm, accelerations and decelerations should be documented |
| Definition normal FHR | Normal baseline 110-150 |
| Definition abnormal FHR | Not described |
| Rhythm | Not described |
| Assessment uterine contractions | Interval and duration of contractions |
| Assessment fetal movements | Interval and duration |
| Other observations | Not described |
| Other recommendations | Intermittent auscultation is recommended for women with low risk of complications, including the following conditions: age>40 y, previous bariatric surgery, BMI>30, GBS positive culture during pregnancy, PAPP-A<0,30 MoM during first trimester, well regulated thyroid disease, induction of labour with AROM/balloon catheter and PROM>24 h.  If the FHR is not normal, auscultation should be prolonged over at least three contractions. |
| Target users | Not described |
| **Norway, 2014**  Yli B, Kessler J, Eikeland T, Henriksen T, Hjelle S, Blix E, et al. Fosterovervåking under fødsel, avnavling og syre-baseprøver fra navlesnor. In: Øian P, Jacobsen AF, Kessler J, editors. Veileder i fødselshjelp. Oslo: Norsk gynekologisk forening; 2014. | |
| Recommendations |  |
| Device(s) used for IA | Pinard or hand-held Doppler device |
| Frequency of auscultation 1^st^ stage | 15-30 minutes |
| Frequency of auscultation 2^nd^ stage | At least every 5 minutes |
| Timing of auscultation | During and after a contraction |
| Duration of auscultation | At least 60 seconds |
| Description of FHR | Not described |
| Definition normal FHR | Normal baseline 110-150 |
| Definition abnormal FHR | Not described for auscultation, only for CTG |
| Rhythm | Not described |
| Assessment uterine contractions | Not described |
| Assessment fetal movements | Not described |
| Other observations | Not described |
| Other recommendations | Admission CTG is not recommended for low risk women.  Intermittent auscultation is recommended as fetal monitoring during labour for low risk women.  Continuous CTG should be assessed after 45-60 min of active pushing in the second stage. |
| Target users | Professionals providing maternity care, pregnant and laboring women. |
| **Sweden, 2015**  Herbst A, Amer-Wahlin I, Stjernholm YV, Weichselbraun M, Domellöf M. Fosterövervakning vid aktiv förlossning (Fetal surveillance in active labour)2015. | |
| Recommendations |  |
| Device(s) used for IA | Not described |
| Frequency of auscultation 1^st^ stage | 15-30 minutes |
| Frequency of auscultation 2^nd^ stage | After each contraction |
| Timing of auscultation | Immediately after a contraction |
| Duration of auscultation | At least 60 seconds |
| Description of FHR | Not described |
| Definition normal FHR | Normal baseline 110-160 |
| Definition abnormal FHR | Not described |
| Rhythm | Not described |
| Assessment uterine contractions | Contractions should be assessed hourly |
| Assessment fetal movements | Not described |
| Other observations | Maternal heart rate |
| Other recommendations | Admission CTG is recommended in all women.  Intermittent auscultation or intermittent CTG (20-30 min CTG every second hour with intermittent auscultation between) is recommended for low risk women. Intermittent auscultation or continuous CTG is recommended during second stage of labour in low risk women.  Continuous CTG is recommended after 30 min of active pushing in low risk women. |
| Target users | Not clearly described |
| **England and Wales, 2014 (updated 2017)**  National Institute of Health and Care Excellence. Intrapartum care. Care of healthy women and their babies during childbirth. National Collaborating Centre for Women's and Children's Health; 2014. | |
| Recommendations |  |
| Device(s) used for IA | Pinard stethoscope or hand-held Doppler device |
| Frequency of auscultation 1^st^ stage | 15 minutes |
| Frequency of auscultation 2^nd^ stage | Not specified |
| Timing of auscultation | Immediately after a contraction |
| Duration of auscultation | At least 60 seconds |
| Description of FHR | As a single rate, and accelerations and decelerations recorded if heard |
| Definition normal FHR | Normal baseline 110-160 |
| Definition abnormal FHR | Rising baseline or decelerations |
| Rhythm | Not described |
| Assessment uterine contractions | Not described |
| Assessment fetal movements | Fetal movements should be recorded |
| Other observations | Palpate maternal pulse if fetal heart rate is suspected to differentiate between the two rates. Palpate maternal pulse hourly, or more often if there are concerns |
| Other recommendations | Admission CTG is not recommended for low-risk women presenting in spontaneous labour.  Intermittent auscultation is recommended for women with low risk of complications, including women with non-significant meconium if there are no other risk factors.  CTG is not recommended in low risk women.  If a rising baseline or decelerations are suspected, carry out auscultation more frequently, e g after three consecutive contractions. |
| Target users | Those who work in or use the National Health Service (NHS) in England and Wales, in particular: midwives, obstetricians, obstetric anaesthetists, neonatologists, maternity support workers and any healthcare professional involved in care of women during labour and birth in any setting  Those responsible for commissioning and planning healthcare services, including primary care trust and local health board commissioners, Wales commissioners, and public health and trust managers  Pregnant women, their families, birth supporters and other carers. |
| **Canada, 2007, reaffirmed 2018**  Liston R, Sawchuck D, Young D. No. 197b-Fetal Health Surveillance: Intrapartum Consensus Guideline. J Obstet Gynaecol Can. 2018;40(4):e298-e322. | |
| Recommendations |  |
| Device(s) used for IA | Not described |
| Frequency of auscultation 1^st^ stage | 15-30 minutes |
| Frequency of auscultation 2^nd^ stage | 5 minutes |
| Timing of auscultation | Baseline FHR should be assessed between contractions |
| Duration of auscultation | For 60 seconds, at least 30 seconds |
| Description of FHR | Not described |
| Definition normal FHR | Normal baseline 110-160 |
| Definition abnormal FHR | Tachycardia: FHR > 160 bpm for > 10 min. Bradycardia: FHR < 110 bpm for > 10 min |
| Rhythm | Dysrhythmia is when there is irregular heart rate not associated with uterine activity |
| Assessment uterine contractions | Palpation of contractions |
| Assessment fetal movements | Not described |
| Other observations | Maternal pulse should be checked during auscultation. |
| Other recommendations | Admission CTG is not recommended.  Intermittent auscultation is recommended for women with low risk for complications. |
| Target users | All health professionals who provide antepartum and intrapartum care in Canada |
| **Australia and New Zealand, 2014**  Royal Australian and New Zealand College of Obstetrics and Gynaecology (RANZCOG). Intrapartum fetal surveillance. Clinical guideline - Third Edition 2014. Victoria: Royal Australian and New Zealand College of Obstetrics and Gynaecology (RANZCOG); 2014 December. | |
| Recommendations |  |
| Device(s) used for IA | Not described |
| Frequency of auscultation 1^st^ stage | Every 15-30 min (during active phase) |
| Frequency of auscultation 2^nd^ stage | After each contraction or at least every 5 min. |
| Timing of auscultation | Each auscultation period should commence toward the end of a contraction and continue for at least 30-60 sec after the contraction has finished. |
| Duration of auscultation | At least 60 seconds |
| Description of FHR | Not described for IA, only for CTG |
| Definition normal FHR | Normal baseline 110-160 |
| Definition abnormal FHR | Not described for IA |
| Rhythm | Not described |
| Assessment uterine contractions | Not described |
| Assessment fetal movements | Not described |
| Other observations | Fetal and maternal pulse should be differentiated |
| Other recommendations | Intermittent auscultation is recommended as an appropriate method for intrapartum fetal monitoring in women without recognized risk factors |
| Target users | Health care professionals providing intrapartum care to pregnant women in established labour in Australia and New Zealand. |
| **USA, American College of Nurse-Midwives, 2015**  American College of Nurse Midwives. Intermittent Auscultation for Intrapartum Fetal Heart Rate Surveillance: American College of Nurse-Midwives. Journal of Midwifery & Women's Health. 2015;60(5):626-32. | |
| Recommendations |  |
| Device(s) used for IA | Fetoscope or hand-held Doppler ultrasound |
| Frequency of auscultation 1^st^ stage | 15-30 minutes |
| Frequency of auscultation 2^nd^ stage | 5 minutes |
| Timing of auscultation | From peak of contraction and for a short period of time after. To assess baseline, auscultation should be performed between contractions and when the fetus is not moving |
| Duration of auscultation | At least 15-60 seconds to assess baseline FHR |
| Description of FHR | Counted FHR, rhytm, and presence or absence of accelerations and decelerations |
| Definition normal FHR | Category I is: normal baseline, 110-160, regular rhythm and absence of FHR decreases or decelerations from the baseline |
| Definition abnormal FHR | Category II include any of the following: irregular rhythm, presence of FHR decreases or decelerations from the baseline, tachycardia (>160>10 min) or bradycardia (<110>10 min) |
| Rhythm | Regular or irregular |
| Assessment uterine contractions | Uterine activity pattern |
| Assessment fetal movements | Yes |
| Other observations | Not described |
| Other recommendations | Intermittent auscultation is the preferred method for monitoring the FHR during labor for women at term who at the onset of labor are at low risk for developing fetal academia.  The frequency of auscultation should be individualized based upon the contraction pattern, level of maternal activity, and institution interventions that may affect FHR (vag.exam., rupture of membrans).  Multiple-count methods appear to be more accurate and reliable than single-count methods for evaluation of periodic changes. |
| Target users | Not described |
| **USA, Association of Women’s Health, Obstetric and Neonatal Nurses, 2015**  Association of Women's Health OaNN. Fetal Heart Monitoring. Nursing for Women's Health. 2015;19(6):557-60. | |
| Recommendations |  |
| Device(s) used for IA |  |
| Frequency of auscultation 1^st^ stage | At least hourly during latent stage of labour, every 15-30 minutes during active first stage of labour |
| Frequency of auscultation 2^nd^ stage | Every 15 minutes during passive fetal descent and every 5-15 min during active pushing |
| Timing of auscultation | Not described |
| Duration of auscultation | Not described |
| Description of FHR | Not described |
| Definition normal FHR | Not described |
| Definition abnormal FHR | Not described |
| Rhythm | Not described |
| Assessment uterine contractions | Not described |
| Assessment fetal movements | Not described |
| Other observations | Not described |
| Other recommendations | Not described |
| Target users | RNs, certified nurse-midwives (CNMs), certified midwives (Ms), other advanced practice nurses such as nurse practitioners and clinical nurse specialists, physicians, and physician assistants |
