# Supplementary material for: Intermittent auscultation fetal monitoring during labour: A systematic scoping review to identify methods, effects, and accuracy
Source: PLoS One. 2019 Jul 10;14(7):e0219573. doi: 10.1371/journal.pone.0219573 (PMC6619817; doi:10.1371/journal.pone.0219573)
Supplement: S1 Fig — Figures suggesting decision making on fetal heart monitoring at admission or first contact and during active labour. (DOCX) [file pone.0219573.s005.docx]

**S1 Figure. Decision-making framework for fetal heart monitoring of low-risk women**

**
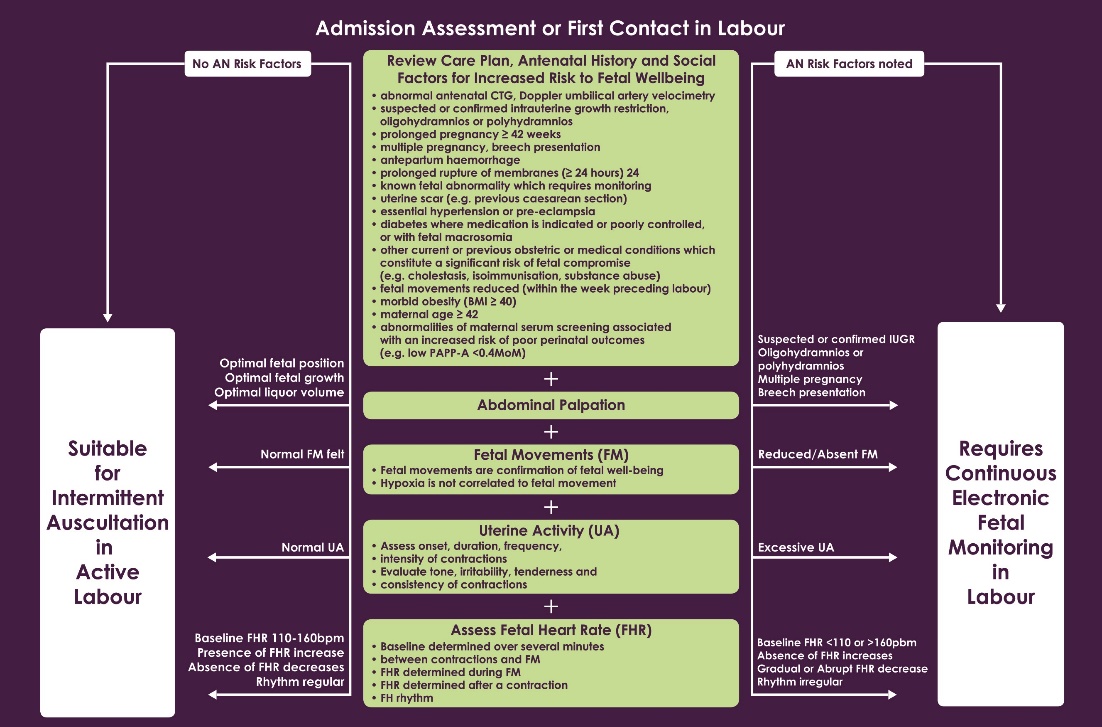
**

Maude RM, Skinner JP, Foureur MJ. Intelligent Structured Intermittent Auscultation (ISIA): evaluation of a decision-making framework for fetal heart monitoring of low-risk women. BMC Pregnancy & Childbirth 2014;14:184.

Fig 2 Intelligent Structured Intermittent Auscultation (ISIA) Informed Decision-making framework for Admission Assessment or First Contact in Labour.

Reprinted with kind permission from the publisher.

**
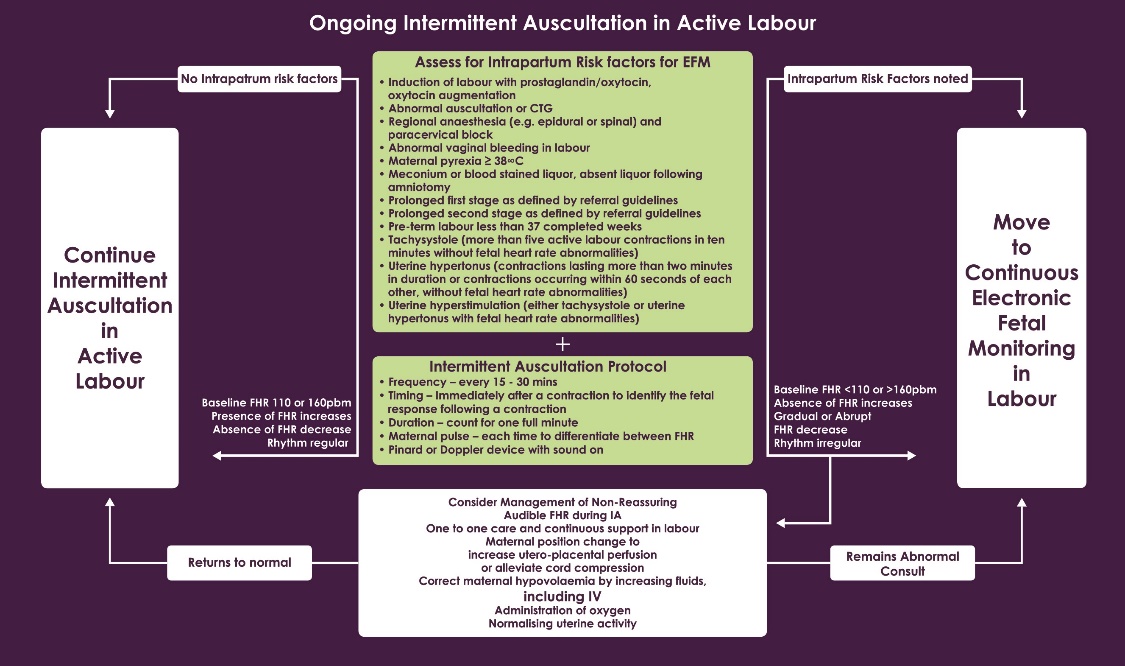
**

Maude RM.,Skinner JP, Foureur MJ. Intelligent Structured Intermittent Auscultation (ISIA): evaluation of a decision-making framework for fetal heart monitoring of low-risk women. BMC Pregnancy & Childbirth 2014;14:184.

Fig3 Intelligent Structured Intermittent Auscultation (ISIA) Informed Decision-making framework for Ongoing Fetal Heart Rate Monitoring in Active Labour.

Reprinted with kind permission from the publisher.
