# Supplementary material for: Intermittent auscultation fetal monitoring during labour: A systematic scoping review to identify methods, effects, and accuracy
Source: PLoS One. 2019 Jul 10;14(7):e0219573. doi: 10.1371/journal.pone.0219573 (PMC6619817; doi:10.1371/journal.pone.0219573)
Supplement: S1 Text — A detailed description of the methods and results of meta-analyses and GRADE assessment of the body of evidence. Table 1. Risk of bias assessment Table 2. Meta-analyses with forest plots and sensitivity analyses Table 3. GRADE assessment of the overall quality of evidence (“Summary of findings”). (DOCX) [file pone.0219573.s006.docx]

**S1 Text. Effects of IA performed with a Doppler device vs. a Pinard stethoscope.**

**Study selection and data extraction methods**

The selection criteria were as follows:

- *Population:* Women in labour
- *Intervention:* Handheld Doppler device for fetal monitoring
- *Comparator:* Pinard fetoscope for fetal monitoring
- *Outcomes:* Primary neonatal outcome: Apgar score <7 at five minutes; primary maternal outcome: Caesarean section. Secondary neonatal outcomes: Composite neonatal outcome (fresh stillbirths, neonatal death within 24 hours, admissions to Neonatal Intensive Care Unit); Stillbirth and neonatal death. Secondary maternal outcomes: Assisted vaginal delivery; Detection of abnormal FHR.
- *Study design:* Randomised controlled trial

Two of the reviewers (EB, LMR) extracted data from each study using a predesigned chart.

**Assessments and synthesis**

The studies that met the inclusion criteria were critically appraised using the Risk of Bias Tool (1). Two reviewers (EB, LMR) assessed the studies independently.

Numbers of babies or women with the outcome of interest were extracted from all studies. Outcomes were analysed by calculating the pooled risk ratio (RR) with 95 % confidence intervals (CI) and a random-effect model (2).

We did GRADE assessment of overall quality of the evidence in a “summary of findings” table. The quality of the evidence was assessed as high, moderate, low or very low (3).

**Description of the included studies**

The four includes studies were performed in Zimbabwe (4), Uganda (5) and Tanzania (6, 7). One of the studies (4) was a four-arm trial where the interventions were fetal monitoring by 1) intermittent CTG, 2) handheld Doppler device by a research midwife, 3) Pinard by a research midwife and 4) Pinard by the midwife in charge. As monitoring with intermittent CTG was beyond the scope of the present review, we did not include arm 1 in the meta-analyses. Arm 2 was included in the Doppler device group, and arms 3 and 4 in the Pinard group. We included a total of 8436 women and their babies.

We assessed the overall risk of bias as unclear in two studies, and low in two (Table S6-1).

| **Table 1 Risk of bias assessment** | | | | |
| --- | --- | --- | --- | --- |
| Paper | Mahomed 1994 (4) | Byaruhanga 2015 (5) | Kamala 2018 (6) | Mdoe 2018 (7) |
| Adequate sequence generation | Low risk of bias | Low risk of bias | Low risk of bias | Low risk of bias |
| Allocation concealment | Unclear risk of bias | Low risk of bias | Low risk of bias | Unclear risk of bias |
| Blinding of participants and personnel | High risk of bias  (Not possible) | High risk of bias  (Not possible) | High risk of bias  (Not possible) | High risk of bias  (Not possible) |
| Blinding of outcome assessor | Unclear risk of bias | Unclear risk of bias | Unclear risk of bias | Unclear risk of bias |
| Incomplete outcome data addressed | Low risk of bias | Low risk of bias | Low risk of bias | Low risk of bias |
| Free of selective reporting | Unclear risk of bias | Low risk of bias | Unclear risk of bias | Low risk of bias |
| Free of other bias* | Unclear risk of bias | Low risk of bias | Unclear risk of bias | Low risk of bias |
| Total quality judgement | Unclear risk of bias | Low risk of bias | Unclear risk of bias | Low risk of bias |

*Other bias: possible performance bias, groups not comparable at baseline

Three of the studies were published between 2015-2018, included women with low or relatively low risk for complications and used the same Doppler device (FreePlay wind-up handheld Doppler, Power-free Education and Technology, Cape Town, South Africa) (5-7). The fourth study (4) were published in 1994, included women with high risk for complications and used Huntleigh Doppler ultrasound monitors. We therefore conducted sensitivity analyses excluding the fourth study (Table S6-2).

**Effects of Doppler device vs. Pinard stethoscope**

In women randomised to IA by different devices, abnormal FHR was detected more often than in the Doppler device group than in the Pinard group (RR 1.77; 95% CI 1.29-2.43). However, this did not affect the clinical outcomes, as there were no significant differences in any of the other outcomes.

The sensitivity analyses resulted in lower heterogeneity across the studies, but did not affect results significantly.

| **Table S2 Meta-analyses with forest plots and sensitivity analyses** |
| --- |
| Apgar score < 7 at 5 minutes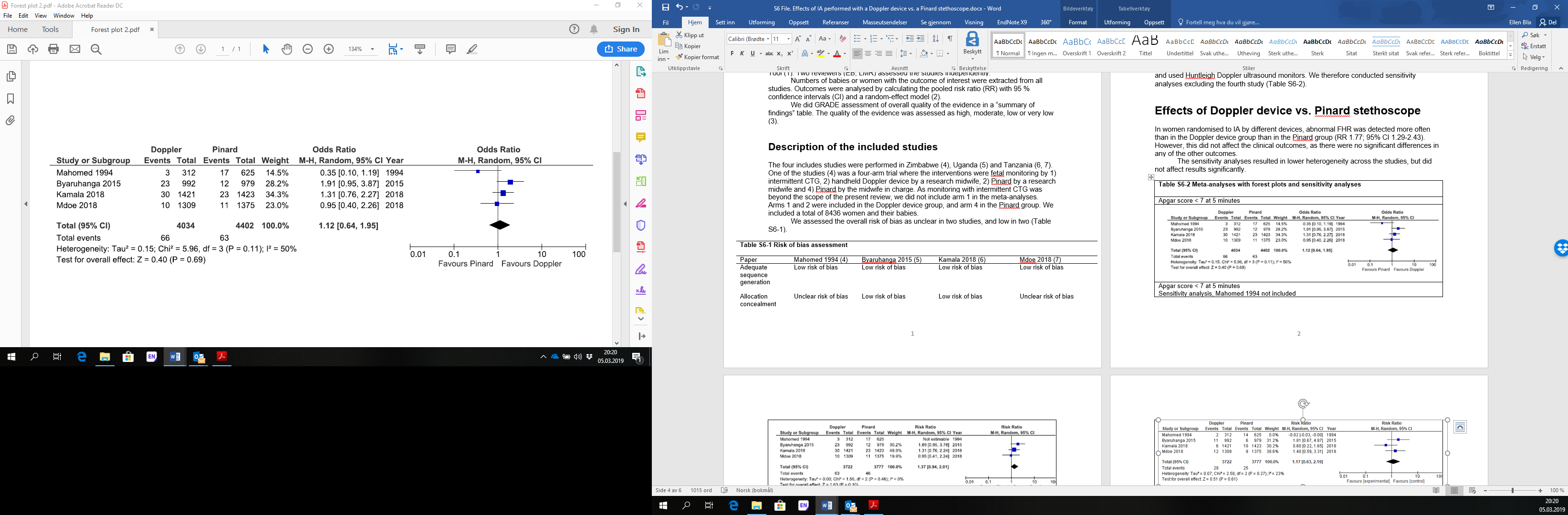 |
|  |
| Apgar score < 7 at 5 minutes  Sensitivity analysis, Mahomed 1994 not included |
| 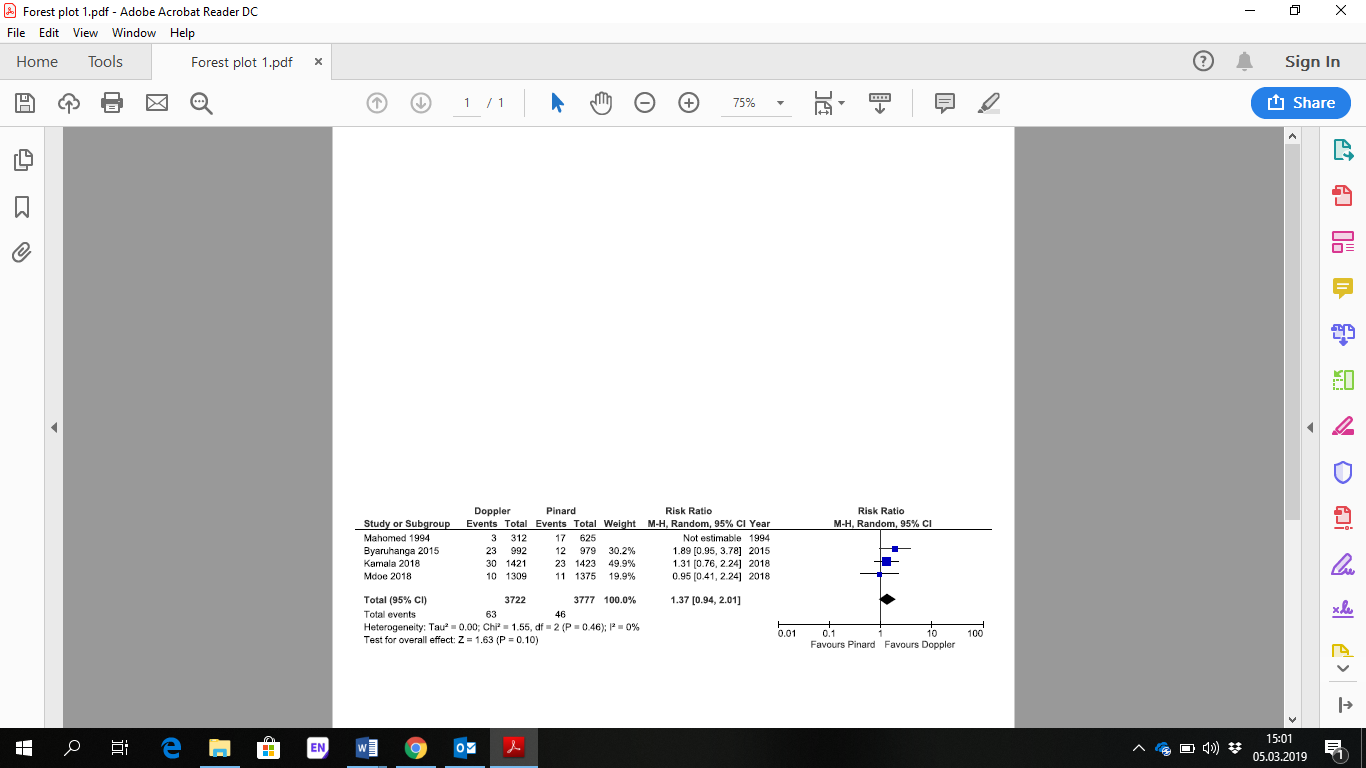 |
| Caesarean section |
| 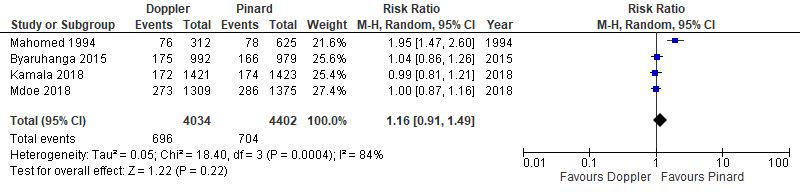 |
| Caesarean section  Sensitivity analysis, Mahomed 1994 not included |
| 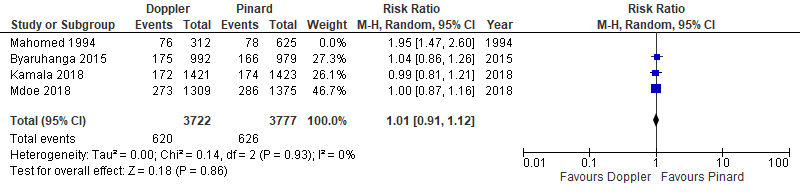 |
| Composite neonatal outcome |
| 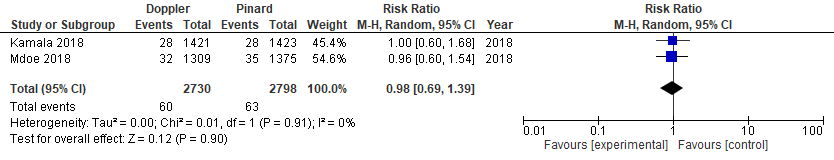 |
| Stillbirth and early neonatal death |
| 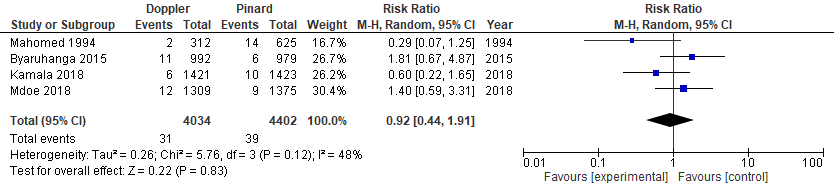 |
| Stillbirth and early neonatal death  Sensitivity analysis, Mahomed 1994 not included |
| 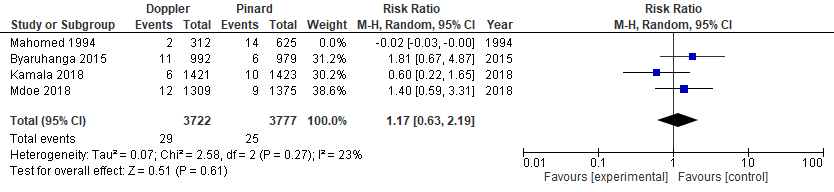 |
| Operative vaginal delivery |
| 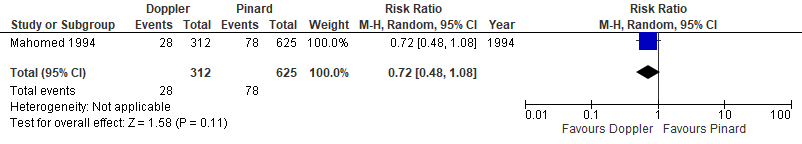 |
| Detection of abnormal FHR |
| 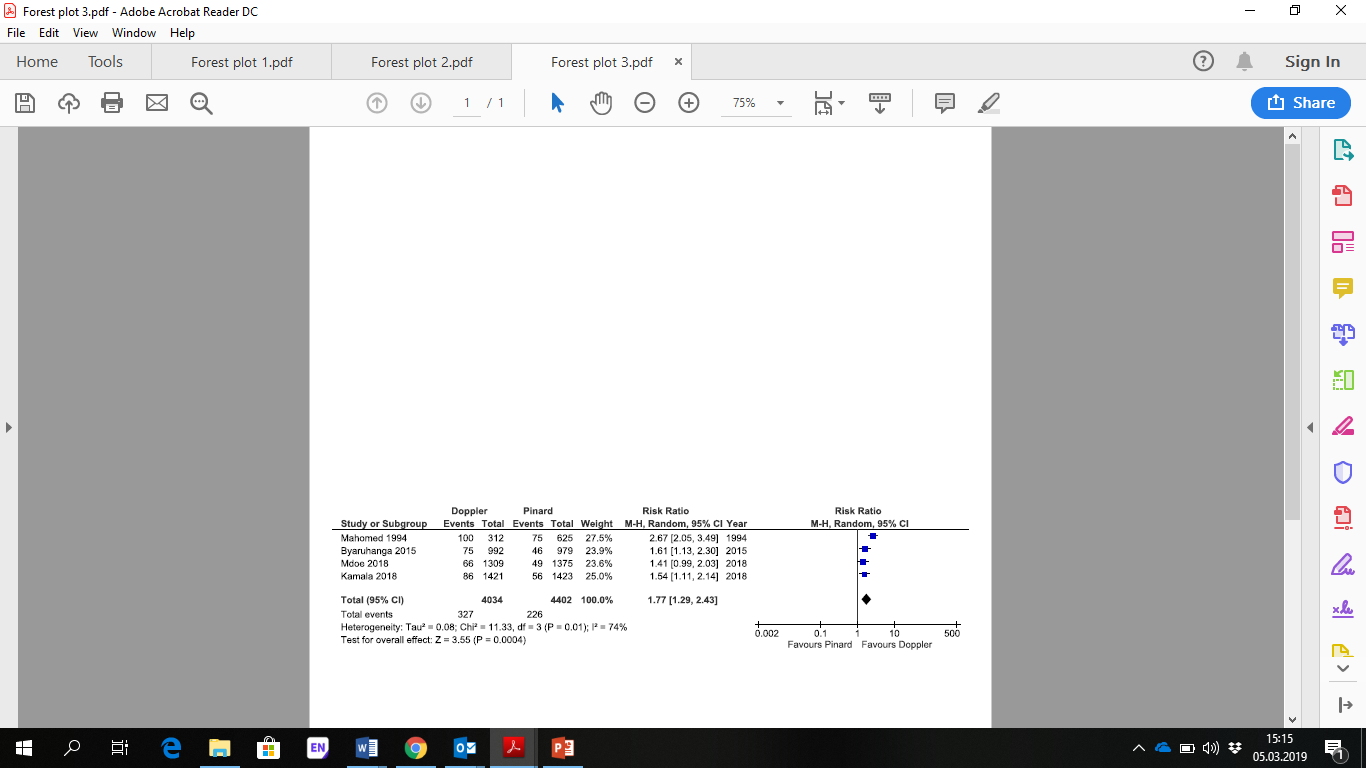 |
| Detection of abnormal FHR  Sensitivity analysis, Mahomed 1994 not included |
| 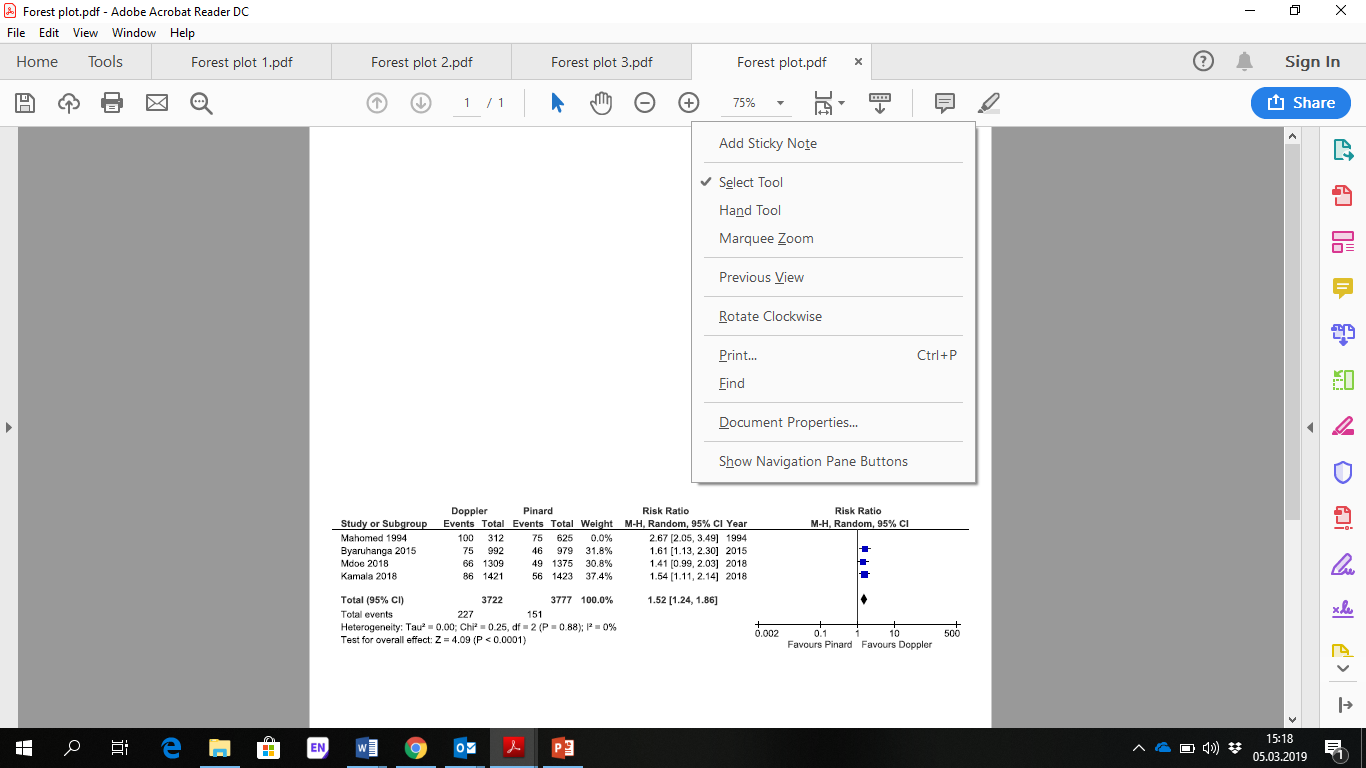 |

**GRADE assessment of the overall quality of evidence**

The GRADE assessment of the overall quality of evidence found low confidence in the effect estimates, except for stillbirth and neonatal birth, which were assessed as moderate (Table S6-3).

Table 3 GRADE assessment of the overall quality of evidence (“Summary of findings”)

| 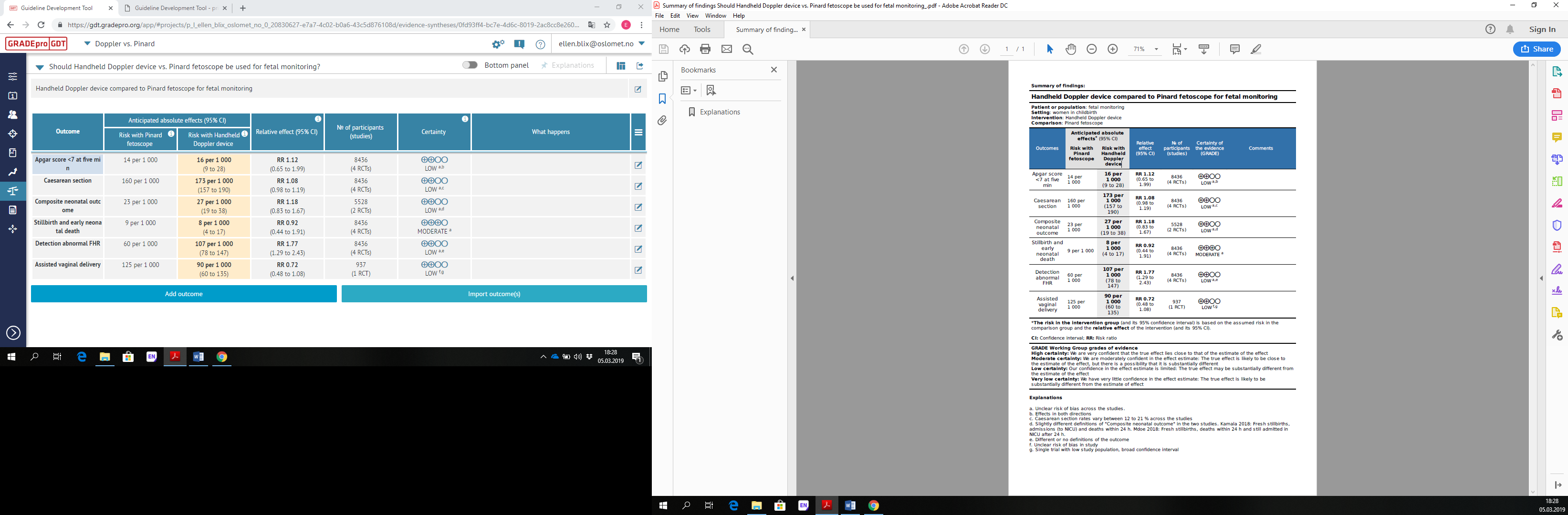 |
| --- |

1. Higgins J, Altman D, Sterne J. Assessing risk of bias in included studies. In: Higgins JPT, Green S. Cochrane handbook for systematic reviews of interventions (Version 6): The Cochrane Collaboration; 2018. Available from <http://handbook.cochrane.org>

2. Higgins JPT DJ, Altman DG. Special topics in statistics. In: Higgins JPT, Green S. Cochrane handbook for systematic reviews of interventions (Version 6): The Cochrane Collaboration; 2018. Available from <http://handbook.cochrane.org>

3. Schünemann H BJ, Guyatt G, Oxman A. GRADE Handbook: The GRADE Working Group; 2013. Available from: <https://gdt.gradepro.org/app/handbook/handbook.html>.

4. Mahomed K, Nyoni R, Mulambo T, Kasule J, Jacobus E. Randomised controlled trial of intrapartum fetal heart rate monitoring. BMJ. 1994; 308: 497-500. PMID: 8136665

5. Byaruhanga R, Bassani DG, Jagau A, Muwanguzi P, Montgomery AL, Lawn JE. Use of wind-up fetal Doppler versus Pinard for fetal heart rate intermittent monitoring in labour: a randomised clinical trial. BMJ Open. 2015; 5: e006867. DOI: [10.1136/bmjopen-2014-006867](https://doi.org/10.1136/bmjopen-2014-006867) PMID: 25636792

6. Kamala BA, Wangwe PJ, Dalen I, Mduma E, Perlman JM, Ersdal HL. Intrapartum fetal heart rate monitoring using a handheld Doppler versus Pinard stethoscope: a randomized controlled study in Dar es Salaam. Int J Womens Health. 2018; 10: 341-8. DOI: [10.2147/IJWH.S160675](https://doi.org/10.2147/IJWH.S160675) PMID: 30022861

7. Mdoe PF, Ersdal HL, Mduma ER, Perlman JM, Moshiro R, Wangwe PT, et al. Intermittent fetal heart rate monitoring using a fetoscope or hand held Doppler in rural Tanzania: a randomized controlled trial. BMC pregnancy and childbirth. 2018; 18: 134. DOI: [10.1186/s12884-018-1746-9](https://doi.org/10.1186/s12884-018-1746-9) PMID: 29728142
