# Supplementary material for: Intermittent auscultation fetal monitoring during labour: A systematic scoping review to identify methods, effects, and accuracy
Source: PLoS One. 2019 Jul 10;14(7):e0219573. doi: 10.1371/journal.pone.0219573 (PMC6619817; doi:10.1371/journal.pone.0219573)
Supplement: S2 Text — The file contains the full protocol. As Prospero does not allow publication of systematic scoping review protocols, we published the protocol at Open Science Framework, 04.03.2017. DOI 10.17605/OSF.IO/KFT6K | ARK c7605/osf.io/kft6k. (DOCX) [file pone.0219573.s007.docx]

**S2 Text. Protocol**

**Published 04.03.2017 at Open Science Framework**

**Intermittent auscultation (IA) as fetal monitoring during labour: a systematic scoping review to identify methods of IA and their effects**

Ellen Blix^1^, Anne Kaasen^1^, Aase Serine Devold Pay^2^, Elisabeth Hals^3^, Sezer Kisa^1^, Elisabeth Karlsen^1^, Ellen Aagaard Nøhr^4^, Ank de Jonge^5^, Helena Lindgren^6,^ Soo Downe^7^, Maralyn Foureur^8^ and Robyn Maude^9^.

*^1^Faculty of Health Sciences, Oslo and Akershus University College, Oslo, Norway; ^2^Department of Hospital Services, Norwegian Directorate of Health, Oslo, Norway; ^3^Department of Obstetrics and Gynaecology, Innlandet Hospital Trust, Lillehammer, Norway; ^4^Institute of Clinical Research, University of Southern Denmark, Odense, Denmark; ^5^Department of Midwifery Science, AVAG and the Amsterdam Publich Health research institute, VU University Medical Center, Amsterdam, The Netherlands; ^6^Department of Women’s and Children’s Health, Karolinska Institute, Stockholm, Sweden; ^7^Soo Downe, School of Community Health and Midwifery, University of Central Lancashire, Preston, UK; ^8^Faculty of Health, University of Technology, Sydney, Australia; ^9^Graduate School of Nursing, Midwifery and Health, Victoria University, Wellington, NZ.*

**Introduction**

The aim of fetal monitoring during labour is to monitor the health of the fetus, to identify those that could be at risk of neonatal and long-term injury, to reassure labouring women and staff that all is well in most cases, and to intervene in a timely manner where deviations from normal are observed. Though a range of techniques can be used for fetal monitoring in labour, including maternal perception, the current standard is to assess the fetal heart rate in conjunction with uterine contractions. There are two main modalities for fetal heart rate monitoring – Intermittent auscultation (IA) and continuous electronic monitoring by using cardiotocography (CTG). There is international consensus that IA should be used for monitoring women with uncomplicated pregnancies (low risk) during labour and birth. Though there is no evidence for improved outcomes for routine use of CTG in complicated labours (high risk), authoritative guidelines recommend that this is the optimal approach to use in these situations (1, 2).

IA is the technique of listening to and counting the fetal heart rate for short periods of time during active labour, in the early stages of labour and more frequently after the diagnosis of the expulsive phase of labour. IA is usually performed by using a Pinard stethoscope or a hand-held Doppler device, and the uterine contractions are palpated by hand.

The Pinard is a hollow tube often made of wood or metal. The size of the Pinard varies across different countries ranging from 15 cm to 30-50 cm. It amplifies sounds associated with the opening and closing of the ventricular valves in the fetal heart, via bone conduction, with each fetal cardiac cycle. With this type of device, the midwife can hear the actual fetal heart sounds, including any abnormal heart beat rhythms. The advantages of the Pinard are that it is inexpensive, available in all settings, no consumables are needed and the fetus is not exposed to unnecessary ultrasound waves. Disadvantages of the Pinard are that it is difficult to use in some maternal positions, may be difficult in obese women and that it probably requires (re)training and time to become skilled. The woman and her partner cannot hear the fetal heartbeats, and there is also no external output that can be shared, transmitted or stored.

The Doppler device is a small hand-held ultrasound transducer that uses the “Doppler effect” to provide an audible simulation of the fetal heartbeats. The advantages of the Doppler device are that it can be used in various maternal positions, including in the water, and that the real-time audio sound is shared with everyone in the room at the same time. Disadvantages are that it needs consumables, is more expensive and exposes the fetus to ultrasound waves. Interpretation of the Doppler recording requires (re)training and time to become skilled, and there is no external output that can be shared, transmitted,or stored. To our knowledge, there is very little research comparing the effect of the Pinard and the continuous wave Doppler device (3).

The CTG was introduced to clinical practice in the 1960s. The aim of the new technology was to reduce perinatal mortality and morbidity due to hypoxia during labour, especially cerebral palsy (CP) (4). CTG is a complex technology where the fetal heart rate and uterine contractions are monitored continuously and printed on paper or shown on a screen. The CTG soon became widespread in the Western world, without any evidence of its effectiveness to prevent morbidity and mortality. However, it is widely promoted by obstetricians and other maternity care staff and providers who believe in its beneficial effect (4, 5). The advantages are that the CTG recording is available for external scrutiny, can be transmitted electronically to and from remote locations, and can be stored. The disadvantages are that, unless wireless technology is used, women are connected to the CTG machine, and so are less likely to be mobile in labour, and interpretation of the CTG tracing requires (re)training and time to become skilled. The machines and consumables are expensive and require sustainable and accessible electrical power sources.

Randomised controlled trials (RCTs) comparing IA and CTG performed in the 70-, 80- and 90s have shown that CTG was associated with lower risk of neonatal seizures in babies born following prolonged labours and/or labours augmented with oxytocin (medication to speed up the labour process). However, this was not associated with long term damage, such as cerebral palsy (6). CTG was associated with an increased risk for a caesarean section and operative vaginal delivery and restricted the mobility of women in labour (7, 8).

Based on the best available evidence international and national guidelines recommend IA in healthy women and babies, and CTG in women with complications (1, 2, 9, 10). Despite of this, CTG is used far more often than recommended. In women monitored by IA, the Doppler device is used more often than the Pinard. There is no scientific evidence for the ideal timing, frequency and durations of IA.

The skills in performing IA – especially by using the Pinard – have deteriorated during the last decades. Fetal monitoring with the Pinard was used in all births across the world until about 40 years ago, and both midwives and obstetricians were skilled in using it. When the use of CTG and Doppler became widespread, midwives and obstetricians have become less skilled around IA (11). The Pinard is still in some use among midwives, but probably not among obstetricians. As IA monitoring leads to fewer unnecessary interventions and is a safe option for the majority of women and babies in labour, who are healthy, it is recommended in these cases, (9, 10). At the same time, there is no evidence and little guidance for the best way of performing IA.

Aims

1. To systematically map and categorise different methods of IA (tools used and how IA is performed, interpreted and managed)
2. To systematically map any effects or accuracy where these have been measured

A review of this kind has the potential to give an overview of the field, identify and describe methods and practices for performing IA, map the evidence (or lack of evidence) for different methods and identify research gaps. A preliminary search for existing scoping reviews on the topic has been conducted. We did not find any scoping reviews or protocols.

**Methods**

As our aims are to produce a map of all different methods of performing IA during childbirth and their effects, advantages and disadvantages, a scoping review is the most suitable method. Scoping reviews allow researchers to examine all types of research literature and grey literature within their chosen area and apply a systematic approach to mapping the literature, methods, evidence, theories and research gaps within a specified area (12).

**Inclusion criteria**

In scoping reviews, the inclusion criteria should be based on clearly identifiable populations, concepts and contexts (PCC) (12).

- Population
  - Women in labour
  - Primi- and multiparous women
  - Healthy women and babies and those with complications
- Concept
  - All forms of IA
  - Devices
  - Modes of counting fetal heart rate
  - Intervals of monitoring
- Context
  - 1) Scientific articles:
  - There will be no language or geographic limitations in the searches for scientific articles.
  - 2) Guidelines:
  - Because of timing and cost, we will limit the searches to international guidelines and national guidelines from UK, Australia, New Zealand, USA, Canada, the Scandinavian countries and the Netherlands.

**Framework for performing the scoping review**

This scoping review will be guided by the five-stage framework described by Arksey and O’Malley (13) and The Joanna Briggs Institute (12).

**Stage 1: Identifying the research question**

IA is the recommended mode of fetal monitoring in low risk childbirth in the majority of countries in the Western world. There is a range of different techniques and protocols and methods for performing IA. There is little research on what frequency, timing and durations are the most effective.

***Research question***

What different techniques and protocols for performing IA in healthy women and babies are described in the literature, what are the outcomes of the use of these methods, and how have these methods been evaluated?

***Study objectives***

- To identify different methods for performing IA
  - Devices
  - How frequently the fetal heart rate is auscultated in one hour period
  - The duration of auscultation, in seconds or minutes
  - How it is counted (e.g. for a full minute or more, 5 secs*12, 6 secs*10, 15 secs*4, 5+5+5 secs)
  - When it is counted in relation to contractions; before, during, how long after
  - How are normal and abnormal heart rates defined in terms of baseline, variability, accelerations and decelerations and how this varies according to the time in labour (latent or active dilatation, second stage)
  - Whether and how contractions are identified in relation to IA
  - How IA is recorded in the woman’s records/casenotes
  - Who is performing the IA
- To identify studies evaluating the effects, predictive values, advantages and disadvantages of the different methods
- To identify gaps in the medical/midwifery literature around IA in healthy women and babies, and areas for future research.

**Stage 2: Identifying relevant studies/literature**

We will develop a search strategy together with a research librarian. The search strategy will follow a three-step Process recommended by the Joanna Briggs institute (12). The first step is an initial search of two databases relevant to the topic (MEDLINE and Cinahl). The second step is an analysis of index terms and text words contained in the titles, abstracts and index lists of the retrieved articles (EB, AK, EH, AaDP). Across all included databases (MEDLINE, Cinahl, Embase, SveMed+, Maternity & Infant care, The Cochrane Library, Web of Science, Lilacs, AJOL and Scopus), new searches based on all identified index terms and text words will be performed. Third step, will be tracing references using relevant articles to identify other key articles, both by scanning bibliographies and by tracing citing articles (Fig. 1).

**Stage 3: Study selection**

The study selection will be conducted in two steps. First, two of the reviewers will independently screen all titles and abstracts to determine eligibility based on the inclusion criteria. The titles will be screened and labelled as “included”, “excluded” and “uncertain”. All articles labelled “included” and “uncertain” by at least one of the reviewers will be considered in the second step. In the second step, pairs of two reviewers (AK, EB, EH, AaDP) will read the full text and apply the inclusion criteria. In case of disagreements, a third reviewer (RM) will be involved to reach consensus.

Formal assessment of the quality of the literature will not be performed as the aim of a scoping review is to present a map of what is already done and what evidence exists rather than finding the best available evidence.

**Stage 4: Charting the data**

In scoping reviews, the data extraction process is called charting the results. The charting process will generate a descriptive summary of the results which is in line with the aims and research questions of the review.

We have developed a data charting form that will be used for extracting information and data from the studies (Fig, 1). Data to be extracted will include: type and year of publication, authors, where the research was conducted, objectives, research methods, details on population, concept and context and details about a priori themes. Additional themes may emerge during the review process, and the data charting form includes a category to describe other themes that might be of interest. For guidelines, there are categories for specific practice recommendations and what they were based on.

If necessary, the data charting form will be further refined and updated during the review process.

**Stage 5: Collating, summarising and reporting the results**

The purpose of the scoping review is to summarise and describe the results across studies and other literature, and not to synthesise specific results (12-14). We will use a modified PRISMA flow chart (15) to present the research process (Fig. 1). We will present a numerical overview of the amount, type and distribution of included studies, guidelines and textbooks. The results may be classified due to the concepts described above. The results will comprise a descriptive or thematic summary of the findings.

Conclusion

A scoping review may be complex and time consuming. Planning the review by a pre-defined protocol is important as it pre-defines objectives and methods, and allows transparency of the process. The review will have relevance to researchers, clinicians, policymakers and pregnant women. It may identify gaps in the literature and guide future research. It may also lead to a better knowledge base for teaching IA to midwifery students, midwives, obstetricians and other involved in care around childbirth.

The research team includes midwives from different countries with both long clinical practice and research experience, and a research librarian.

End of study: 31^st^ December 2017.

1. Lewis D, Downe S, Panel FIFMEC. FIGO consensus guidelines on intrapartum fetal monitoring: Intermittent auscultation. Int J Gynaecol Obstet. 2015;131:9-12.

2. Ayres-de-Campos D, Spong CY, Chandraharan E, Panel FIFMEC. FIGO consensus guidelines on intrapartum fetal monitoring: Cardiotocography. Int J Gynaecol Obstet. 2015;131:13-24.

3. Mahomed K, Nyoni R, Mulambo T, Kasule J, Jacobus E. Randomised controlled trial of intrapartum fetal heart rate monitoring. BMJ. 1994;308:497-500.

4. Banta HD, Thacker SB. Electronic fetal monitoring. Lessons from a formative case of health technology assessment. Int J Technol Assess Health Care. 2002;18:762-70.

5. Bloom SL, Belfort M, Saade G, Eunice Kennedy Shriver National Institute of Child H, Human Development Maternal-Fetal Medicine Units N. What we have learned about intrapartum fetal monitoring trials in the MFMU Network. Semin Perinatol. 2016.

6. Grant A, O'Brien N, Joy MT, Hennessy E, MacDonald D. Cerebral palsy among children born during the Dublin randomised trial of intrapartum monitoring. Lancet. 1989;2:1233-6.

7. Alfirevic Z, Devane D, Gyte GM. Continuous cardiotocography (CTG) as a form of electronic fetal monitoring (EFM) for fetal assessment during labour. Cochrane Database Syst Rev. 2013;5:CD006066.

8. SBU. Fosterövervakning med kardiotokografi (CTG) vid förlossning. Stockholm: Statens beredning för medicinsk utvärdering, 2015 11. juni 2015. Report No. 6.

9. National Institute of Health and Care Excellence. Intrapartum care. Care of healthy women and their babies during childbirth. National Collaborating Centre for Women's and Children's Health, 2014.

10. Yli B, Kessler J, Eikeland T, Henriksen T, Hjelle S, Blix E, et al. Fosterovervåking under fødsel, avnavling og syre-baseprøver fra navlesnor. In: Øian P, Jacobsen AF, Kessler J, editors. Veileder i fødselshjelp. Oslo: Norsk gynekologisk forening; 2014.

11. Maude RM, Skinner JP, Foureur MJ. Putting intelligent structured intermittent auscultation (ISIA) into practice. Women and birth : journal of the Australian College of Midwives. 2015.

12. The Joanna Briggs Institute. Methodology for JBI Scoping Reviews. Adelaide: The Joanna Briggs Institute; 2015.

13. Arksey H, O'Malley L. Scoping studies: towards a methodological framework. International Journal of Social Research Methodology. 2005;8:19-32.

14. Levac D, Colquhoun H, O'Brien KK. Scoping studies: advancing the methodology. Implement Sci. 2010;5:69.

15. Liberati A, Altman DG, Tetzlaff J, Mulrow C, Gotzsche PC, Ioannidis JP, et al. The PRISMA statement for reporting systematic reviews and meta-analyses of studies that evaluate healthcare interventions: explanation and elaboration. BMJ. 2009;339:b2700.

Initial limited search of MEDLINE and CINAHL. Analysis of keywords text words in titles and abstracts. Prepare new searches based on all identified keywords as index terms.

(n=X)

Records identified through searches in MEDLINE, Cinahl, Embase, SveMed+, Maternity & Infant care, The Cochrane Library, Web of Science, Lilacs, AJOL and Scopus

(n=X)

Records identified through other sources (reference tracing in relevant articles, personal knowledge)

(n=x)

Records excluded (n=X)

Full-text articles excluded, with reasons

(n = X)

Included in scoping review:

Articles: (n=X)

Assessing and charting data from full text articles (n=x)

Records after duplicates removed

(n = X)

Records screened (n=X)

Fig 1. Modified Prisma flow diagram.
