# Supplementary material for: Intermittent auscultation fetal monitoring during labour: A systematic scoping review to identify methods, effects, and accuracy
Source: PLoS One. 2019 Jul 10;14(7):e0219573. doi: 10.1371/journal.pone.0219573 (PMC6619817; doi:10.1371/journal.pone.0219573)
Supplement: S3 Text — During the study, we found it appropriate to change some of our plans. All deviations from the original protocol are described in the file. (DOCX) [file pone.0219573.s008.docx]

**S3 Text. Deviations from the protocol**

The protocol states that there will be no language restrictions in the searches for scientific articles. The searches have no restrictions, as we identified a huge amount of literature, we realised that we did not have resources for translation. We therefore decided to include articles in languages any in the study group could understand: Danish, Dutch, English, French, German, Icelandic, Norwegian, Swedish and Turkish.

In the protocol, we wrote that according to the Joanna Briggs recommendations (1), we would not do quality assessment of the literature. After reading through the included guidelines, we found that the quality was varying, and decided to do formal quality assessments by using the AGREE tools (2).

We had decided to summarise and describe the results and not to synthesise specific results. As we unexpectedly identified four randomised trials (3-6), we decided to include them in a meta-analysis. We followed the methods of the Cochrane Handbook (7), including GRADE assessments of the overall quality of the evidence (8).

Liv Merete Reinar joined the author group in June 2018.

Our plan was to finish the study by the end of 2017. The study period was extended by 14 months, therefore we did a full update of the literature searches, in all relevant databases, in January 2019.

**References**

1. The Joanna Briggs Institute. Methodology for JBI Scoping Reviews. Adelaide: The Joanna Briggs Institute; 2015. Available at: <http://joannabriggs.org/assets/docs/sumari/Reviewers-Manual_Methodology-for-JBI-Scoping-Reviews_2015_v2.pdf>

2. Brouwers M, Kho ME, Browman GP, Cluzeau F, Feder G, Fervers B, et al. Appraisal of Guidelines for Research & Evaluation II (AGREE II). Can Med Assoc J. 2013; 182: E839-42. doi: [10.1503/cmaj.091714](https://dx.doi.org/10.1503%2Fcmaj.091714) PMID: [20513780](https://www.ncbi.nlm.nih.gov/pubmed/20513780)

3. Mahomed K, Nyoni R, Mulambo T, Kasule J, Jacobus E. Randomised controlled trial of intrapartum fetal heart rate monitoring. BMJ. 1994; 308: 497-500. PMID: 8136665

4. Byaruhanga R, Bassani DG, Jagau A, Muwanguzi P, Montgomery AL, Lawn JE. Use of wind-up fetal Doppler versus Pinard for fetal heart rate intermittent monitoring in labour: a randomised clinical trial. BMJ Open. 2015; 5: e006867. DOI: [10.1136/bmjopen-2014-006867](https://doi.org/10.1136/bmjopen-2014-006867) PMID: 25636792

5. Mdoe PF, Ersdal HL, Mduma ER, Perlman JM, Moshiro R, Wangwe PT, et al. Intermittent fetal heart rate monitoring using a fetoscope or hand held Doppler in rural Tanzania: a randomized controlled trial. BMC pregnancy and childbirth. 2018; 18: 134. DOI: [10.1186/s12884-018-1746-9](https://doi.org/10.1186/s12884-018-1746-9) PMID: 29728142

6. Kamala BA, Wangwe PJ, Dalen I, Mduma E, Perlman JM, Ersdal HL. Intrapartum fetal heart rate monitoring using a handheld Doppler versus Pinard stethoscope: a randomized controlled study in Dar es Salaam. Int J Womens Health. 2018; 10: 341-8. DOI: [10.2147/IJWH.S160675](https://doi.org/10.2147/IJWH.S160675) PMID: 30022861

7. Higgins JPT, Green S. Cochrane handbook for systematic reviews of interventions (Version 6): The Cochrane Collaboration; 2018. Available from <http://handbook.cochrane.org>

8. Schünemann H BJ, Guyatt G, Oxman A. GRADE Handbook: The GRADE Working Group; 2013. Available from: https://gdt.gradepro.org/app/handbook/handbook.html.
